# Targeting Essential Hypothetical Proteins of *Pseudomonas aeruginosa* PAO1 for Mining of Novel Therapeutics: An *In Silico* Approach

**DOI:** 10.1155/2023/1787485

**Published:** 2023-04-11

**Authors:** Atikur Rahman, Md. Takim Sarker, Md Ashiqul Islam, Mohammad Uzzal Hossain, Mahmudul Hasan, Tasmina Ferdous Susmi

**Affiliations:** ^1^Department of Genetic Engineering and Biotechnology, Faculty of Biological Science and Technology, Jashore University of Science and Technology, Jashore 7408, Bangladesh; ^2^Department of Chemistry and Biochemistry, University of Windsor, Canada; ^3^Bioinformatics Division, National Institute of Biotechnology, Ganakbari, Ashulia, Savar, Dhaka 1349, Bangladesh; ^4^Department of Pharmaceuticals and Industrial Biotechnology, Sylhet Agricultural University, Sylhet 3100, Bangladesh

## Abstract

As an omnipresent opportunistic bacterium, *Pseudomonas aeruginosa* PAO1 is responsible for acute and chronic infection in immunocompromised individuals. Currently, this bacterium is on WHO's red list where new antibiotics are urgently required for the treatment. Finding essential genes and essential hypothetical proteins (EHP) can be crucial in identifying novel druggable targets and therapeutics. This study is aimed at characterizing these EHPs and analyzing subcellular and physiochemical properties, PPI network, nonhomologous analysis against humans, virulence factor and novel drug target prediction, and finally structural analysis of the identified target employing around 42 robust bioinformatics tools/databases, the output of which was evaluated using the ROC analysis. The study discovered 18 EHPs from 336 essential genes, with domain and functional annotation revealing that 50% of these proteins belong to the enzyme category. The majority are cytoplasmic and cytoplasmic membrane proteins, with half being stable proteins subjected to PPIs network analysis. The network contains 261 nodes and 269 edges for 9 proteins of interest, with 11 hubs containing at least three nodes each. Finally, a pipeline builder predicts 7 proteins with novel drug targets, 5 nonhomologous proteins against human proteome, human antitargets, and human gut flora, and 3 virulent proteins. Among these, homology modeling of NP_249450 and NP_251676 was done, and the Ramachandran plot analysis revealed that more than 94% of the residues were in the preferred region. By analyzing functional attributes and virulence characteristics, the findings of this study may facilitate the development of innovative antibacterial drug targets and drugs of *Pseudomonas aeruginosa* PAO1.

## 1. Introduction

*Pseudomonas aeruginosa*, often termed as an opportunistic pathogen, is a rod-shaped, motile, Gram-negative, and nonfermenting bacteria found ubiquitously in soil and water as well as found in colonies on the animate part of plants and animals including humans [1, 2]. Isolates collected from diverse environments reported 272 species of the *Pseudomonas* genus in which *Pseudomonas aeruginosa* PA01 is one of the most commonly used laboratory strains as well as employed to generate publicly accessible genomic resources [2, 3]. *Pseudomonas aeruginosa* PA01 is the first-ever strain of its species having a completely sequenced genome from a chronic lesion isolate dated from the 1950s. The genome is 6.3 Mbp long that includes 5570 ORFs, roughly 89.4% coding regions, and 0.4% stable RNAs. This was the largest bacterial genome available during the year 2000 when sequenced. However, despite the same species, different genomic and phenotypic changes are found across isolates of *P. aeruginosa* PA01 strains stored in different laboratories worldwide [2, 4].

A broad spectrum of host targets including nematodes, insects, plants, and mammals are susceptible to infection by *P. aeruginosa* species [2]. It is found harmless in normal gut microflora but causes dangerous infections in critically ill ICU patients [5]. This trend in pathogenesis makes them opportunistic pathogens [2]. It is regarded to be within the top three causative agents for infection caused by opportunistic pathogens annually in the community as well as related to (10-15%) of hospital-acquired infections [6]. In 2015, a report from the European Antimicrobial Resistance Surveillance Network (EARS-Net) on European regions revealed that around 13.7% of strains of *P. aeruginosa* had acquired resistance to a minimum of three antimicrobial communities whereas about 5.5% of the strains were resistant against five antimicrobial groups. Every year in the USA alone, roughly 440 deaths and 51,000 infection cases are caused by *P. aeruginosa* of which over 13% result from multidrug resistant *Pseudomonas* strains. As a consequence, *P. aeruginosa* has been announced as one of the greatest threats to public health amongst the 12 bacterial families from the antibiotic-resistance priority pathogens enlisted by WHO in 2017 [7]. It is also involved with some other nosocomial infections like bloodstream infection, gastrointestinal infection, and urinary tract infection [5]. This bacterium poses a devastating impact on lung disease patients with cystic fibrosis (CF). Apart from CF, it is equally deadly for individuals having compromised immune systems like AIDS, cancer, burn lesions, and eye injuries. The situation can get even worse despite having robust antibiotic medication since *P. aeruginosa* possess a wide spectrum of resistance against antibiotics including aminoglycosides, *β*-lactams, and fluoroquinolones. Therefore, disease stress subsequently results in organ failure and eventually death [2, 5].

*Pseudomonas aeruginosa* adopts some survival strategy that helps them to resist environmental stressors and dodge host immune responses [8]. Some of these survival tools include biofilm formation, enzyme promiscuity, horizontal gene transfer, and quorum sensing [5]. It is one of the well-studied strains for investigating the bacterial biofilm formation process [9]. Three polysaccharides, alginate, Pel, and Psl, were discovered to be important for bacterial attachment and biofilm formation in *P. aeruginosa* PA01 [8]. Over 500 regulatory genes have been recorded from the *P. aeruginosa* PA01 genome investigation [10]. There is still a lot to discover for a better understanding of the intracellular signaling pathways and several other regulatory mechanisms involving many proteins that are still uncharacterized. Thus, domain analysis and functional annotation of essential hypothetical proteins (EHPs) can pave the way to identify new potential targets facilitating drug repositioning development. Since these EHPs are needed for cellular, biological, and metabolic processes, their deletion or mutation can be fatal to the species. These prospective drug targets may be crucial in the development of antimicrobial drugs [11].

In this study, an *in silico*-based approach has been adopted for the characterization of proteins with unknown functions via different algorithm-based tools and software. Besides, a network-based analysis was directed to find interaction with critically connected hub proteins that may control major molecular activities together. The pipeline builder was employed to analyze nonhomologous proteins against humans, human antitargets, and the proteome of the gut microbiota, as well as predict virulence factors and novel drug targets. Finally, using reliable software, the structural conformation of our protein of interest with potential druggability was predicted and assessed. Thus, our analysis mainly involves the identification of essential hypothetical proteins in *P. aeruginosa* PA01 and can further lead to the discovery of novel proteins of therapeutic targets.

## 2. Materials and Methods

### 2.1. Sequence Retrieval and Analysis

**Figure 1:**
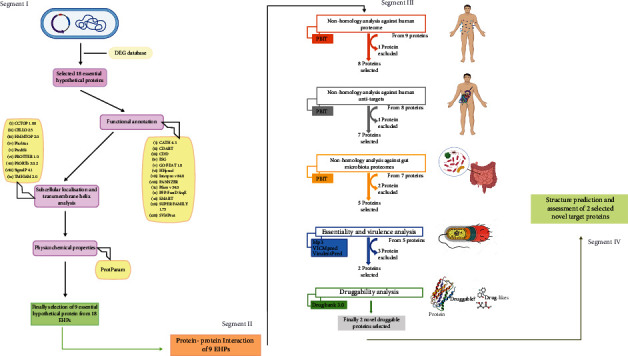
Schematic representation of the whole methodology used in our investigation. There are four segments: segment I: functional annotation and properties characterization; segment II: protein-protein interaction network; segment III: nonhomology analysis, virulence factor prediction, and druggability identification; and segment IV: structure prediction and structure validation.

The full proteome of *P. aeruginosa* PAO1 (strain ATCC 15692) was retrieved from the NCBI genome database. The bacterial complete genome contains 6.3 million base pairs and 5564 proteins [4]. The essential genes database (DEG) is then subjected to find out the essential hypothetical proteins (EHPs) from this complete proteome list by employing a series of unique keywords [12]. To begin, we looked for similar hypothetical proteins where we found 2181 proteins among these 5564 proteins. Following that, we searched for the exact matches of hypothetical proteins and exact matches of conserved hypothetical proteins and found 1540 and 625 hypothetical proteins, respectively. According to the DEG database, this bacterial proteome contains 336 essential proteins (EPs). Essential proteins are those that are inevitable and adequate for a living cell to survive under ideal circumstances. Consequently, we discovered 29 essential hypothetical proteins by manual curation whose genomes were entirely conserved among the 336 EPs. The status (reviewed or unreviewed), annotation score (1–5), structural and functional availability, and other factors were used to further validate these 29 EHPs from the NCBI and UniProt databases. Eventually, we excluded 11 proteins and targeted 18 essential hypothetical proteins whose FASTA sequences were used to facilitate further analysis throughout this study. The complete framework of our investigation is presented in Figure 1, and all the databases/software used in this study are in Table 1.

### 2.2. Segment I: Functional Annotation and Properties Characterization

#### 2.2.1. Functional Annotation and Domain Analysis of EHPs

The functional annotation of 18 *Pseudomonas aeruginosa* EHPs was unveiled by using numerous publicly accessible databases and tools. To gain more knowledge about the molecular functions and biological processes of the EHPs, we consider protein superfamily, family, conserved domain analysis, and Gene Ontology (GO) analysis. Using an online server GO FEAT, for the functional characterization by homology searching through multiple databases such as NCBI, Uniprot, and EMBL, a preliminary assessment was performed to see if any of the HPs were allocated a family and/or protein domain [13]. After preliminary evaluation, proteins conserved domains and protein functions based on domain architecture were determined by using CDART [14] from the conserved domain database (CDD) and SMART [15], respectively. For functional analysis, SUPERFAMILY 1.75 [16], Pfam 34.0 [17], SVMProt [18], CATH 4.3 [19], InterPro 84.0 [20], and HHPred [21] were used to identify the protein superfamily, functional family, domain, and essential sites based on similarity. PANNZER [22], PFP [23], and ESG [24] tools were used for high-throughput functional annotation of EHPs, which provided Gene Ontology information with *z*-scores as well as brief explanations of the annotated protein's functionality. These GO terms facilitate understanding a gene's molecular functions, physiological roles, and cellular mechanism, which refers to the location of the gene's product. We used default parameters for all databases.

#### 2.2.2. Subcellular Localization and Transmembrane Helices Analysis

Subcellular localization of a protein can help to infer much information about that protein's function. In our study, we employed several databases to annotate the subcellular localization of the selected 9 EHPs which include PSORTb [25], CELLO [26, 27], TMHMM [28], Phobius [29], HMMTOP [30], CCTOP [31], PROTTER [32], SignalP 4.1 [33], and PrediSi [34]. According to PSORTb and CELLO, the proteins were distinguished by 5 major cellular positions: cytoplasmic, inner membrane, periplasmic, outer membrane, and extracellular. To predict transmembrane helices, TMHMM, Phobius, HMMTOP, CCTOP, and PROTTER were employed. Information of transmembrane helices location is somehow beneficial for the conformation of possible 3D structures [35]. Besides, it is necessary to find out signal peptides which are the N-terminal part of a protein. Mainly, they are targeted to the endoplasmic reticulum to the secretory pathway, and it is considered the way of protein localization prediction [33]. Signal peptides were identified by using these SignalP 4.1, PROTTER and PrediSi databases.

#### 2.2.3. Analysis of Physicochemical Properties

The Expasy's ProtParam server [36] was utilized for the analysis of the physicochemical properties of 18 selected essential hypothetical proteins (EHPs) which include molecular weight, theoretical pI (isoelectric point), formula, the total number of positively and negatively charged residues, instability index, aliphatic index, and grand average of hydropathicity (GRAVY).

### 2.3. Segment II: Protein-Protein Interaction Network of 9 EHPs

#### 2.3.1. Protein-Protein Interaction Network Analysis

The function of a protein molecule often is modulated by its surrounding protein networks [37]. For this reason, it is important to discover the protein network to get an insight into the functional association of a particular protein [38]. In this study, we have used NetworkAnalyst v3.0 for network building [39]. We have inputted a list of genes containing 9 EHPs with their Uniprot IDs (Q9HXM8, Q9HWT5, Q9HVM2, Q9HVF5, Q9I5H0, Q9HZL8, Q9HYC8, Q9HXV5, and Q9HUH3) since all of these proteins were found stable through the physicochemical analysis. The generic PPI option under protein-protein interactions (PPI) was checked for further processing. The *P. aeruginosa* PA01 interactome database provided with robust computational prediction and experimentally validated data was adopted for network building. Next, the corresponding network was explored for further analysis in Cytoscape. Cytoscape is a standalone software that enables several topological parameter analyses like discovering the shortest possible path, node degree distribution, and clustering hub genes of the network [40].

### 2.4. Segment III: Nonhomology Analysis, Virulence Factor Prediction, and Druggability Identification

#### 2.4.1. Nonhomology Analysis against Human Proteome and Human Antitargets

Several features were needed for the identification of the drug target for any human disease. For this reason, to analyze nonhomology aspects, we tried the pipeline builder for the identification of target (PBIT) server for the nonhomology analysis against human proteome, against human antitargets and human gut flora proteomes [41]. Using the pipeline builder, we first identified human homologous proteins that share high sequence similarity with human proteome. The sequence similarity of the inputted 9 sequences was figured using the BLAST algorithm where the *E* − value > 0.005 and %sequence identity < 50 were set. 8 of the 9 input sequences are nonhomologous that were selected for further investigation. These homologous proteins were filtered to avoid the undesirable toxic-effects for these similarities. Filtered and selected 8 nonhomologous proteins were further employed in the pipeline to recognize nonhomologous proteins against human antitargets, proteins that contain harmful effects due to the impact of a drug named antitargets [41]. To screen out the significant similar sequence with familiar human antitargets, the PBIT database uses the BLAST algorithm where they utilize those human antitarget proteins based on different literature [41]. Again *E* − value > 0.005 and %sequence identity < 50 were set, and all nonhomologous sequences were selected.

#### 2.4.2. Nonhomology Analysis against Human Gut Flora Proteomes

PBIT also analyzes human gut flora proteomes that make it easier to find out those highly similar sequences with human gut microbiota. It is known that gut microbiota plays an important role in human health that includes immune, metabolic, and neurobehavioral characters [42]. That is why it is necessary to design such drugs whose target is nonhomologous protein sequence of the gut microbiome. As result, such drugs could not able to kill or hamper essential microbes found in the human gut. For this, the pipeline builder for identification of target (PBIT) server was again used to identify nonhomologous proteins against gut microbiota proteomes. As the third step of the pipeline builder, selected proteins were employed where *E* − value > 0.001 and %sequence identity < 50 were set. Now nonhomologous proteins were selected for the next investigation.

#### 2.4.3. Analysis of Virulence Factor

Understanding the pathogenesis mechanism through the analysis of virulence factors can be a key to the discovery of new promising therapeutic targets [38]. Therefore, we have used VICMpred [43], VirulentPred [44], and MP3 [45] for the identification of the virulence property of the 9 EHPs. We have collected the results predicted combined by 2 out of the 3 tools. All the results were collected by using the provided default options by the servers.

#### 2.4.4. Druggability Analysis and New Target Identification

Identification of a new drug target can be a new window for the discovery and development of a new drug against infectious or serious diseases [46]. Druggability analysis is the examination of a protein that has the possible capability or binding affinity towards a drug or drug-like molecules. This druggability analysis can introduce a new drug target against a drug. Here we used DrugBank, a comprehensive online database that contains information on drugs and drug targets [47]. Target identification segment was utilized for this purpose, and amino acid sequences in the FASTA format were the search index. All other BLAST parameters and filters were set as default where the expectation value was set to 0.00001.

### 2.5. Segment IV: Structure Prediction and Structure Validation

#### 2.5.1. Secondary Structure Analysis

The interactions between neighboring polypeptides mainly design a protein's secondary structure. When the elements of the secondary structure have folded together among each other, the 3D structure of the protein is formed. The databases namely SOPMA [48] and PSIPRED [49] provide the secondary structure of a protein. These databases were used to predict the structure where the protein sequence in the FASTA format was the searching index for the websites and the rest of the parameters were set as default.

#### 2.5.2. Essential Hypothetical Proteins 3D Structure Modeling

The protein 3D structure was determined based on two methods: template-based homology modeling and trRosetta methods. The three-dimensional structure of the targeted protein was generated using the SWISS-MODEL server, which uses template search and then aligns the target sequence with the template structure to create the homology model [50]. To construct the model with an accuracy equal to low-resolution X-ray crystallography, we only consider templates with ≥30% sequence identity. Then the server Robetta (https://robetta.bakerlab.org/) was employed to predict the 3D model by using the trRosetta algorithm. It is a deep learning method based on direct energy minimizations that is the most accurate process of structure building provided by this server [51]. Finally, the built structure was optimized using Galaxy Refiner, with the best-refined model based on the lowest MolProbity and highest GDT-HA value [52]. Consequently, the PyMOL 2.0 visualization software is used to visualize all of the refined structure files, which are in .pdb format.

#### 2.5.3. Protein Structure Validation Assessment

The reliability of a predicted 3D structure of a protein can be assessed by using various quality assessment tools. Here, in this study, we used SAVES version 6.0 (https://saves.mbi.ucla.edu/) which is a metaserver that runs six programs at once to check and validate protein structure during and after model refinement. This server validates the stereochemical consistency of a protein structure by performing residue by residue geometry and overall structure geometry. Furthermore, it also compares the results to good structures to see if an atomic model (3D) is compatible with its amino acid sequence (1D) by assigning a structural class based on its location and environment (alpha, beta, loop, polar, nonpolar, etc.). We run ERRAT [53], VARIFY 3D [54, 55], PROVE [56], WHATCHECK [57], PROCHECK [58], and Ramachandran plot [58] from SAVES v6.0 to determine the consistency of the constructed model.

### 2.6. Molecular Dynamics Simulation

We used an online-based user-friendly interface to make a recommendation for our predicted protein structures. Here, we used template-based homology modeling and *ab initio* modeling to predict the structure of our targeted protein candidate. We used the WebGro (https://simlab.uams.edu/) GROMACS simulation package to simulate protein in water dynamics simulation for over 50 ns. The simulation system works by the following steps such as preprocessing, energy minimization, equilibration, molecular dynamics, trajectory analysis, and result generation. The trajectory analysis was performed to measure root mean square deviations (RMSD), root mean square fluctuation (RMSF), radius of gyration (Rg), solvent-accessible surface area (SASA), and hydrogen bonds.

### 2.7. Determination of Binding Site

The binding site or active site is known as an important portion of any protein/enzyme. Mainly that particular portion is involved with attaching molecules to initiate a certain reaction. Though our target is to find a novel therapeutic target, it is necessary to find the active sites of our target proteins. To find the active sites, we utilized the website called prank web (https://prankweb.cz/) to determine all possible binding targets of our selected protein.

### 2.8. Performance Assessment of the Study

In our study, we have applied the receiver operating characteristic (ROC) analysis for validating the accuracy of our bioinformatics tools used for the functional annotation of EHPs from *P. aeruginosa* [59]. We have collected 100 arbitrary protein functions of *P. aeruginosa* along with their gene names using the same pipeline used prior to our study in Supplementary file 1 and 2. Two integer values namely “1” as a truly positive and “0” as a truly negative were assigned to classify the prediction. The confidence rating was denoted by “2,” “3,” “4,” and “5,” respectively. The higher number denotes a greater level of confidence. The input file consists of 2 columns where 1st column contains binary numbers like 1 (true positive) and 0 (true negative), and the 2nd column contains a rate of confidence ranging from 2 to 5. For the present study, six levels were considered for determining the diagnostic efficacy. The ROC analysis was used for 12 individual functional annotation tools. The data were submitted to an online-based ROC curve-generating web server in format-1 [60]. The output result includes accuracy, sensitivity, specificity, and the ROC area (Supplementary File 1 and 2). The accuracy of our adopted pipeline is 97.42% which indicates a very high and reliable result for the bioinformatics tools that we used in our study.

## 3. Results

### 3.1. Functional Annotation and Domain Analysis of EHPs

The functional annotation of the 18 EHPs was examined using 12 reliable platforms that predict protein superfamily, family, conserved domains, and Gene Ontology (GO) terms. Here, the functional annotation was assigned with high confidence as we considered only that function that was similar in three or more programs. Consequently, the functional characterization categorizes these proteins into 9 functional categories. The first category was enzymes (deaminases, dehydrogenases, helicases, transferases, DNases, oxidoreductases, kinases, etc.) where nine of the 18 EHPs are included (NP_252456.1, NP_252782.1, NP_253095.1, NP_253326.1, NP_250846.1, NP_252375.1, NP_253678.1, NP_253679.1, and NP_253685.1). The two proteins are transporter proteins (NP_253252.1 and NP_251676.1). The remaining seven proteins are in the rest seven categories that are bacterial outer-membrane protein, folate-binding protein, peptidase inhibitor protein, electron transporter protein, chromosome partition protein, ribosome maturation protein, and pathogenesis-related protein.  Table 2 enlists the 18 EHP superfamily, functional family, molecular functions, and biological functions, as well as their GO IDs and database IDs. Among these proteins, NP_249450.1 is a member of the folate-binding superfamily, with the aminomethyl transferase folate-binding domain as its functional family. Aminomethyl transferase and transaminase activity are the two molecular functions of this protein. Another protein sequence of NP_251676.1 was predicted belonging to the functional family that represents the periplasmic core domain found in a variety of ABC transporters. ATP binding, ATPase-coupled xenobiotic transmembrane transporter activity, efflux transmembrane transporter activity, and ATPase activity are some of the molecular functions of this protein. According to the GO annotation, there were 65 GO terminologies in total for the molecular function and biological process. These GO IDs can be used to retrieve Gene Ontology analysis of these 18 EHPs.

### 3.2. Subcellular Localizations of EHPs

**Figure 2:**
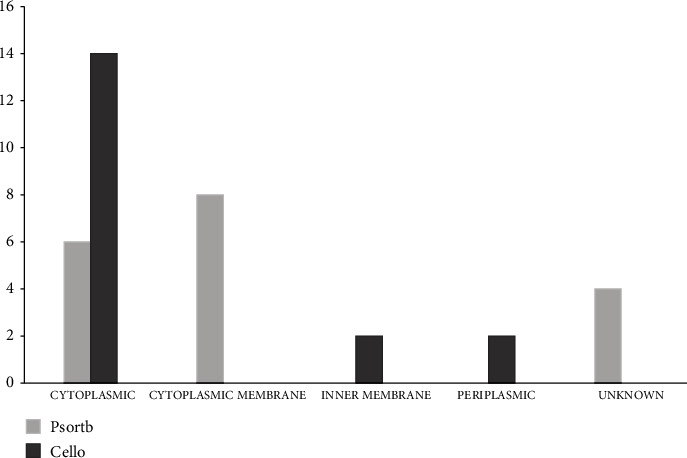
The subcellular localization of 18 EHPs is displayed by the column plot. Five categories of columns are for 5 types of subcellular localization (cytoplasmic, cytoplasmic membrane, inner membrane, periplasmic, and unknown). Here, lighter black represents the data from CELLO database, and the color grey is for the database PSORTb.

To identify the cellular localization of our 18 EHPs, the websites PSORTb and CELLO were utilized. According to the data of PSORTb, among 18 essential hypothetical proteins, 6 proteins belong to cytoplasmic protein, 8 proteins belong to the location of the cytoplasmic membrane, and the remaining 4 proteins are considered unknown. The database CELLO depicted that 14 proteins are cytoplasmic proteins, 2 proteins are considered as inner membrane proteins, and the rest 2 are periplasmic proteins. This is the generalized concept of the cellular location which is shown in Figure 2 and supplementary table 1. The existence of the transmembrane helix was also figured out, and this can help to carry out the function of a protein through transmembrane transportation. The amount of transmembrane helix was given in supplementary table 1. The presence of signal peptide was also investigated from the three websites SignalP 4.1, PROTTER, and PrediSi. Among 18 proteins, 14 proteins (NP_252456.1, NP_252782.1, NP_253095.1, NP_253326.1, NP_253368.1, NP_249450.1, NP_250846.1, NP_252171.1, NP_252375.1, NP_253374.1, NP_253455.1, NP_253678.1, NP_253679.1, and NP_253685.1) do not contain any signal peptide, and one protein (NP_250659.1) contains signal peptide unanimously, whereas the remaining proteins (NP_253252.1, NP_251676.1, and NP_253434.1) are containing signal peptides from any of a website (supplementary table 1).

### 3.3. Physicochemical Properties Analysis

We have searched for the physicochemical properties of 18 EHPs which are shown in Table 3. All the proteins had molecular weights ranging from 13335.11 to 56122.54 Dalton (Da). The highest molecular weight was observed to be 56122.54 Da for the NP_253252.1 protein, a probable lipid II flippaseMurJ [61]. The theoretical pI (isoelectric point) indicates the pH at which the charge of an amino acid of a protein remains neutral. Therefore, no movement occurs when placed in an electric field with a direct current. This parameter comes in handy as proteins are dense and stable at an isoelectric pH [62]. The theoretical pI ranged from 4.52 to 10.71. Both of these parameters (molecular weight and theoretical pI) help visualize the two-dimensional gel electrophoresis or (2-DE) and hence contribute to the scientific examinations of these hypothetical proteins [63]. The aliphatic index can be an effective indicator for determining the thermostability of some protein molecules [64]. A protein molecule with a higher aliphatic index indicates its higher range of temperature at which it gains its thermostability [65]. The aliphatic index tabulated for our protein group ranged from 83.13 to 133.96. The NP_253252.1 protein showed the maximum thermostability and NP_252456.1 with the lowest. The parameter called the instability index determines a protein whether it is stable or unstable in a test tube [66]. For our analysis, we set the cutoff value to 40 where the value below 40 indicates a protein as stable and above 40 predicts it as an unstable protein. A total of 9 proteins (NP_252456.1, NP_252782.1, NP_253252.1, NP_253326.1, NP_249450.1, NP_251676.1, NP_252171.1, NP_252375.1, and NP_253679.1) out of 18 proteins of interest were found to be stable with instability index values of 25.65, 37.46, 36.08, 38.61, 31.65, 38.17, 32.05, 29.03, and 32.97, respectively. The grand average of hydropathy (GRAVY) determines the extent of protein-water interaction which is calculated by dividing the aggregate of all the amino acids' hydropathy values by the total number of residues in the given sequence [65, 67]. The GRAVY values lay between -0.427 and 0.857. The lower the GRAVY value, the more a protein interacts with water [65]. The NP_253095.1 protein was found to be the most interactive among all these proteins having a GRAVY value of -0.427.

### 3.4. Protein-Protein Interaction Network Analysis

**Figure 3:**
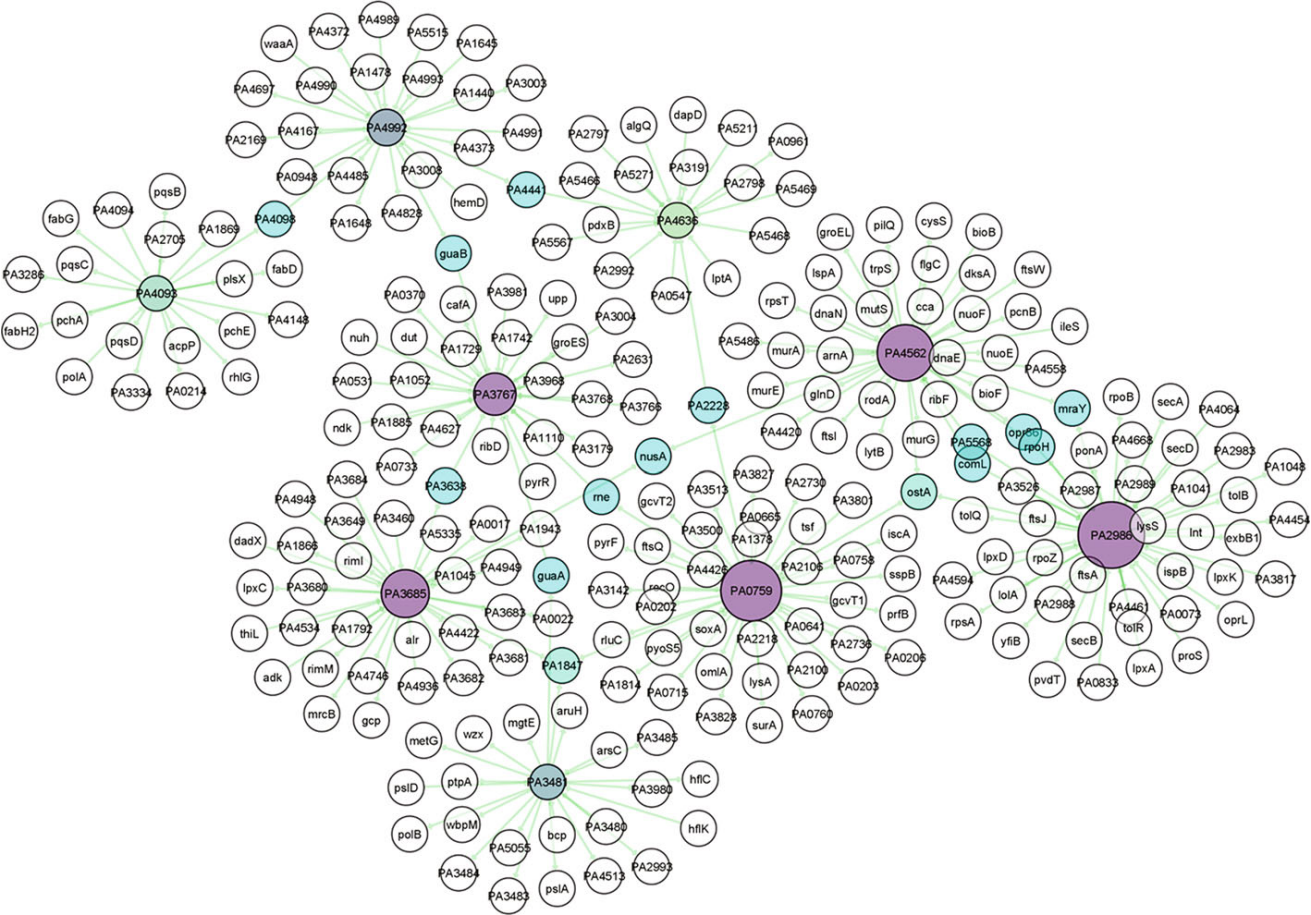
Protein-protein interaction network of 9 stable EHPs collected from *P. aeruginosa* PAO1 protein interactome database. The network has 261 nodes and 269 edges provided with 11 subnetworks (hubs) with a minimum of 3 nodes each. The nodes with lower degree values are colored green namely PA4992, PA3481, PA4093, and PA4636. The color gradually turned into deep purple by the increase of node degree values. The nodes in cyan blue mean 2 or more interactions with their corresponding subnetworks.

The PPI represents the connection among the 9 stable EHPs and their corresponding functionally relative proteins from *P. aeruginosa* PA01. The network has 261 nodes and 269 edges for 9 proteins of interest. Here, the network is provided with 11 subnetworks (hubs) with a minimum of 3 nodes each. The nodes with only 3 connections (degree) are considered islands (ostA and PA1847) (Table 4) [68]. The node degree and betweenness centrality range from 3 to 45 and 4750 to 17881.76, respectively. The interaction among the hub proteins can be seen in Figure 3. The size and color gradient of the nodes determine the degree of a protein. A node degree reveals the extent of interaction of a particular node with other nodes. The nodes with lower degree values are colored green namely PA4992 (24), PA3481 (23), PA4093 (20), and PA4636 (18). The color gradually turned into deep purple by the increase of node degree values. Nodes with enlarged size similarly denote increased node degree values such as PA2986 (45), PA0759 (41), PA4562 (38), PA3685 (32), and PA3767 (28) (Figure 3) (Table 4). The nodes in cyan blue mean 2 or more interactions with their corresponding subnetworks. Betweenness centrality is a topological measure that typically determines the number of shortest paths through nodes. The nodes with a higher degree and betweenness centrality values represent vital proteins for signal trafficking of the cellular system [68]. The function of all proteins in the network is collected from NCBI using their associated Entrez IDs and listed in the supplementary table 2.

### 3.5. Nonhomology Analysis against Human Proteome, Human Antitargets, and Human Gut Flora Proteomes

To introduce a novel target for a drug, it must be nonhomologous against human proteome, human antitargets, and human gut flora proteomes. Utilizing pipeline builder from the pipeline builder for identification of target (PBIT) server, 9 protein sequences were inputted to find out the highly similar sequence with human proteome. Among the 9 EHPs sequences, one sequence was homologous with the human proteome. Filtering that one sequence, 8 nonhomologous proteins were selected for the next pipeline analysis to find out the nonhomologous proteins against human antitargets. Among the 8 entered sequences, significant similar sequences of human antitarget proteins were screened out. This result depicted that 7 proteins are nonhomologous and one protein is homologous to the human antitarget where this one homologous protein was omitted from the study. After the filtration, selected 7 proteins were further inputted onto the pipeline builder to analyze nonhomologous proteins against human gut flora proteomes. This time, 2 proteins were screened out because of containing high sequence similarity with the proteomes of the beneficiary microbes belonging to the human gut. Then finally, the sequences of 5 nonhomologous EHPs were selected for the next parameter of finding virulence capability. The details of the nonhomology analysis are given in Table 5.

### 3.6. Virulence Factor

The virulent EHPs from *P. aeruginosa* PA01 are enlisted in Table 5. VICMpred is a support vector machine- (SVM-) based webserver that predicted all of the 9 EHPs as nonvirulent with 70.75% accuracy [43]. VirulentPred is also based on bilayer cascade SVM with fivefold increased cross-validation methods that give 81.8% prediction accuracy [44]. A total of 3 proteins namely NP_249450.1 (e-106), NP_251676.1 (e-171), and NP_253679.1 (7e-77) were predicted as virulent by VirulentPred in *P. aeruginosa* PA01 strain utilizing the similarity-based search through PSI-BLAST. Another webserver called MP3 uses an integrated SVM-HMM approach which commonly predicted NP_251676.1 as a pathogenic protein.

### 3.7. A Possible New Drug Target Identification

Along with the two virulent EHPs, other 7 sequences of EHPs were employed on the DrugBank server for the identification of potentially new drug candidates. This server showed that NP_252456.1 contains one drug target against the drug Imidazole (*E*-value: 5.62144*e* − 18; bit score: 75.485; query length: 182; alignment length: 77) and two drug targets were exhibited by the protein NP_253679.1 against the drug nicotinamide adenine dinucleotide phosphate (*E*-value: 3.79487*e* − 15; bit score: 72.4034; query length: 270; alignment length: 213) and nicotinamide adenine dinucleotide phosphate (*E*-value: 1.77261*e* − 14; bit score: 70.8626; query length: 270; alignment length: 217). The remaining 7 proteins (NP_252782.1, NP_253252.1, NP_253326.1, NP_249450.1, NP_251676.1, NP_252171.1, and NP_252375.1) were considered a fresh or new drug target by the DrugBank database. This website also revealed that our targeted two proteins named NP_249450.1 and NP_251676.1 displayed zero matches for the drug target which means they are new potential drug candidates with druggability. The overall results are shown in Table 5.

### 3.8. Analyzing Secondary Structure

**Figure 4:**
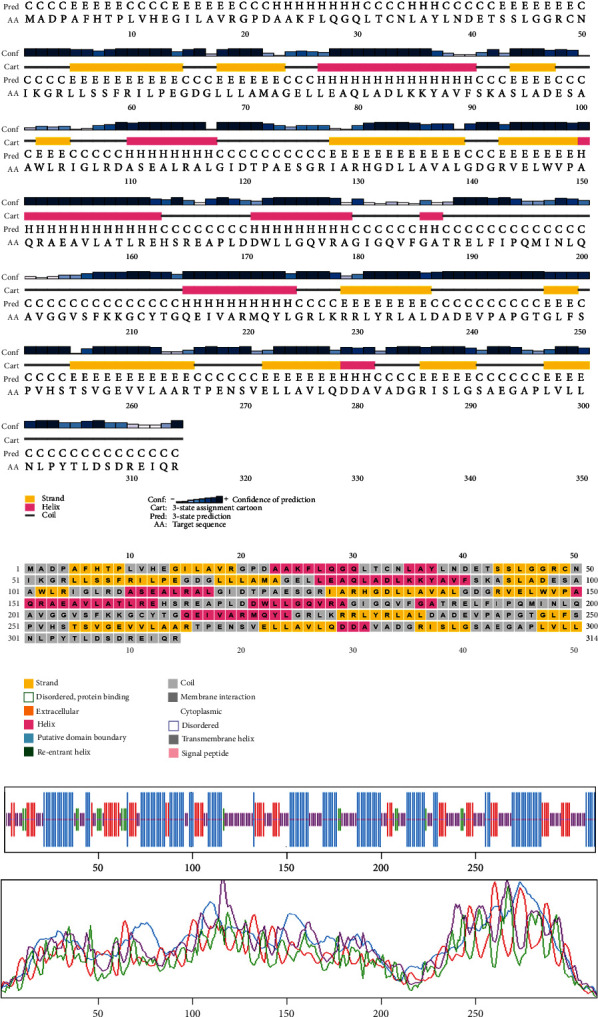
The secondary structure of NP_249450.1 from (a) PSIPRED website and (b) SOPMA websites.

**Figure 5:**
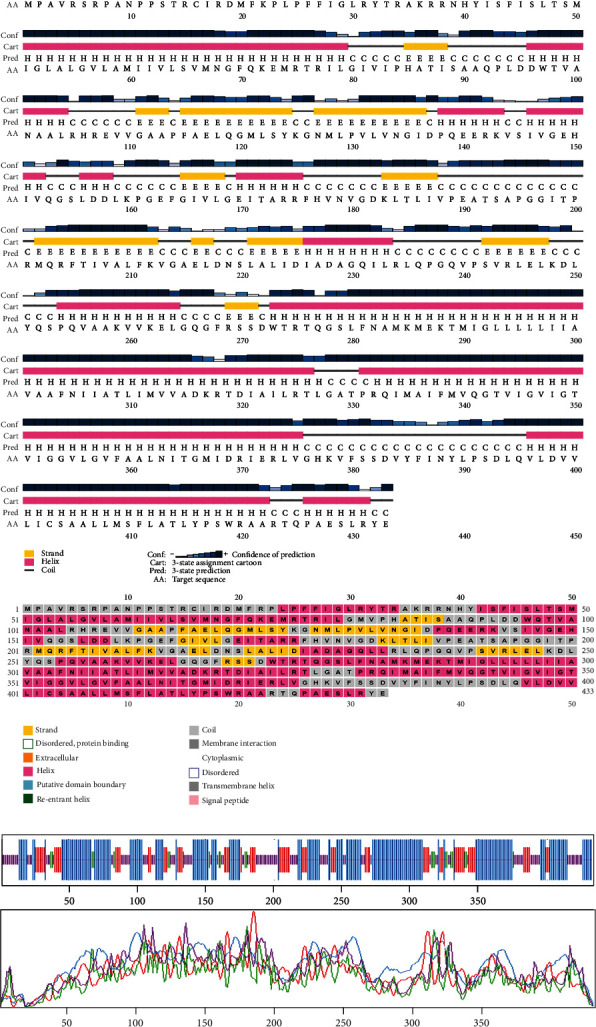
The secondary structure of NP_251676.1 from (a) the PSIPRED server and (b) SOPMA database.

Based on the findings of segment III, we selected two proteins for the next-level investigations that match all the criteria of segment III. As they are hypothetical proteins, they must lack some information. For this, to suggest them as a new drug target, we explored their secondary structure. SOPMA and PSIPRED were the web tools that were used for the secondary structure analysis. According to the SOPMA server, the secondary structure of NP_249450.1 had alpha helix (Hh): 126 (40.13%); extended strand (Ee): 57 (18.15%); beta turn (Tt): 20 (6.37%), and random coil (Cc): 111 (35.35%) where the parameters were set as window width: 17; similarity threshold: 8; and number of states: 4. The protein NP_251676.1 had alpha helix (Hh): 206 (47.58%); extended strand (Ee): 83 (19.17%); beta turn (Tt): 23 (5.31%); and random coil (Cc): 121 (27.94%) with the same parameter as before. The results from SOPMA database for both proteins are given in supplementary table 3. The PSIPRED sequence plot and PSIPRED cartoon plot were provided as a result of the PSIPRED web servers. The sequence plot and cartoon plot structure described that goldenrod (semi-yellow) color is for the extracellular strand domain, pink color is for helix, grey color is for coil, and blackish blue is for the confidence of the structure. According to this, NP_249450.1 showed more coil in its secondary structure whereas NP_251676.1 showed more helix.  Figure 4(a) is the secondary structure of NP_249450.1 from PSIPRED, and Figure 4(b) is from SOPMA websites. Besides, Figure 5(a) is the secondary structure of NP_251676.1 from PSIPRED, and Figure 5(b) is from the SOPMA database.

### 3.9. Essential Hypothetical Proteins 3D Structure Modeling

**Figure 6:**
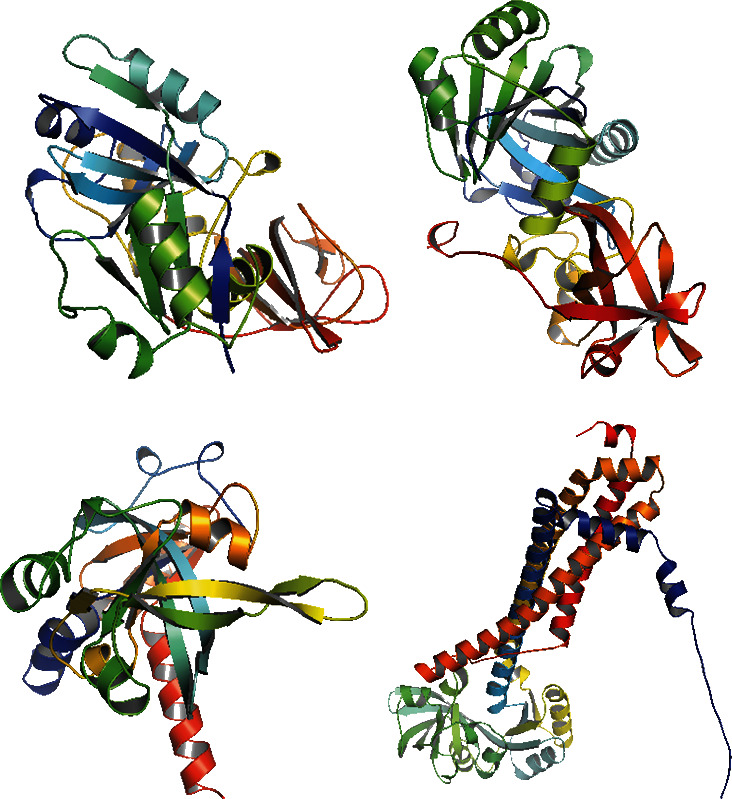
*Three-dimen*sional *homology modeling.* (a) Template-based homology modeling structure of NP_249450.1 from SWISS-MODEL. (b) *Ab initio* modeling structure of NP_249450.1 from the Robetta server. (c) Template-based homology modeling structure of NP_251676.1 from SWISS-MODEL. (d) *Ab initio* modeling structure of NP_251676.1 from the Robetta server.

Only proteins that passed all of the above-mentioned pipeline analyses were assigned a three-dimensional structural conformation. Two proteins, NP_249450.1 and NP_251676.1, were subjected to a thorough pipeline review and thus have the potential to be used as new drug targets. As a result, these two proteins were subjected to 3D structural conformation determination using two methods: template-based homology modeling from SWISS-MODEL and *ab initio* modeling using the trRosetta algorithm from the Robetta server. For template-based homology modeling, we searched for templates from SWISS-MODEL for these two proteins. 1vly.1 and 6f3z.2 were the best template for NP_249450.1 and NP_251676.1, respectively. The templates were chosen based on several parameters, including the Global Model Quality Estimation (GMQE), Qualitative Model Energy ANalysis (QMEAN), *Z*-score, sequence identity, sequence similarity, sequence coverage, and oligo-state of the chosen templates. The template 1vly.1 was actually a 1.30 Å resolution X-ray diffraction crystallography structure of a putative aminomethyltransferase (ygfz) from *E. coli*. This template shared 30.23% sequence identity with the 314 aa long NP_249450.1 protein, which spans from (4-307 aa). The template 6f3z.2, on the other hand, was a complex of *E. coli* LolA and the periplasmic domain of LolC that was also identified by X-ray diffraction crystallography at a resolution of 2.00 Å. The sequence identity was 30.73%, spanning (67-290) amino acids out of the 433 amino acids in the NP_251676.1 protein. Both of these templates had a monomer oligo-state. Finally, using 1vly.1 and 6f3z.2 templates, the structures of NP_249450.1 and NP_251676.1 EHPs were formed, as shown in Figures 6(a) and 6(c). Structure prediction by Robetta server illustrated that the provided model was built using trRefineRosetta modeling (*ab initio* modeling using the trRosettaalgorithm). Structure of NP_249450.1 showed a 0.79 score as confidence (Figure 6(b)) while NP_251676.1 showed a 0.81 score as confidence (Figure 6(d)). Consequently, the structures from SWISS-MODEL were then refined from Galaxy Refiner where model 2 for NP_249450.1 and model 5 for NP_251676.1 were downloaded after final refinement. For the NP_249450.1 and NP_251676.1 proteins, the lowest MolProbity was 1.738 (model 2) and 1.729 (model 5), respectively, while the initial score was 2.280 and 2.299. Also, the structures from Robbetta were refined from Galaxy Refiner.

### 3.10. Protein Structure Validation Assessment

**Figure 7:**
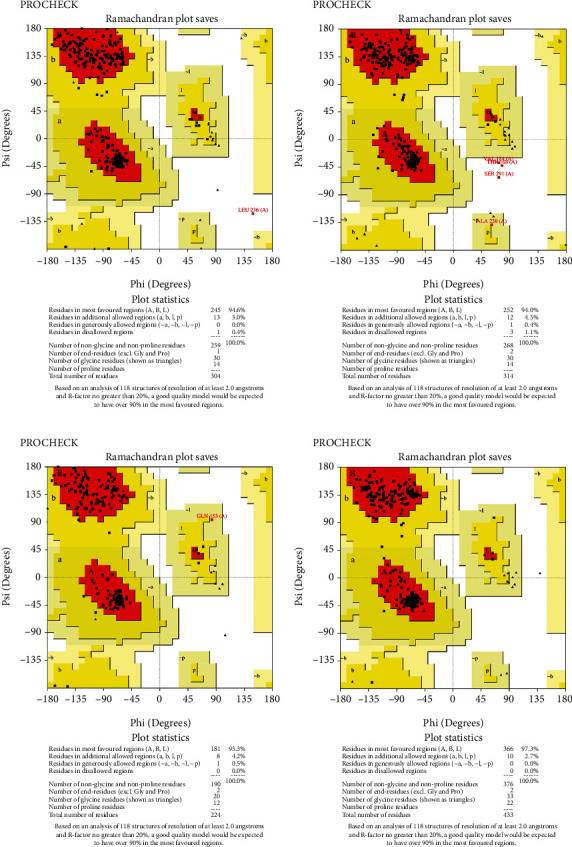
*Three-dimensional structure assessment by Ramachandran plot analysis by PROCHECK*. (a) Ramachandran plot of NP_249450.1 protein from the SWISS-MODEL structure. (b) Ramachandran plot of NP_249450.1 protein from the Robetta model. (c) Ramachandran plot of NP_251676.1 protein from the SWISS-MODEL structure. (d) Ramachandran plot of NP_251676.1 protein from the Robetta model.

The predicted protein structure was validated by the SAVES v6.0 server which runs six programs simultaneously to evaluate the quality of the build model. The ERRAT value served as the model's overall quality element. The overall quality factor for the NP_249450.1 protein structure from SWISS-MODEL and Robetta, respectively, was 87.6325% and 92.459%. It was 93.3649% and 98.063% for NP_251676.1 from these two servers, respectively. In supplementary figure 1, bar plots depict the overall quality factor from ERRAT. VARIFY3D conducts an analysis in which a structure passes if at least 80% of the amino acids in the 3D/1D profile have a score of ≥0.2. Three of the four structures passed this parameter (two from SWISS-MODEL and one from Robetta), while one structure failed for NP_251676.1 from Robetta. WHATCHECK included a color box with a number within it that reflects 46 different criteria, with the green, yellow, and maroon colors representing OK, warning, and error, respectively. The overall summary report is OK for all four structures.  Table 6 included a comprehensive report on the consistency of the four structures that we retrieved from the SAVES v6.0 server. On the contrary, structures from SWISS-MODEL failed to pass the PROVE parameters, while structures from Robetta were placed in warning categories due to atomicB-factors, and the protein atoms having absolute *Z* − scores > 3. Ramachandran plot analysis from the PROCHECK program also demonstrated that more than 94% of residues were in the most favored region for all four structures from both SWISS-MODEL and Robetta. It was 97.3% for NP_251676.1 protein from Robetta, with 0.0% residues in the disallowed region. The Ramachandran plot analysis unveiled that the generated structures of the proteins represent an excellent degree of validity and reliability, which is depicted in Figure 7.

### 3.11. Molecular Dynamics Simulation

Both NP_249450.1 and NP_251676.1 protein structures predicted from the SWISS-MODEL depict that the RMSD value initially follows an upward trend until 5 ns and then stabilizes to 50 ns without major fluctuations. Therefore, these structures have a stable profile. On the other hand, structures predicted by *ab initio* method do not follow a constant trend throughout the 50 ns trajectory. They follow an upward trend, and the RMSD value is not consistent within this 50 ns trajectory. The RMSF measures the average deviation of amino acid residues over a specific time course. It typically measures individual residue flexibility. The structures have a lower residue flexibility as they have a maximum RMSF value < 0.25 nm. The SASA is also analyzed from the simulation trajectories, and it represents that, initially, structures predicted by the *ab initio* method have a higher SASA trend, whereas structures predicted by homology modeling have a lower SASA value than the other model which presents higher stability of the model. Moreover, the Rg value which is a determinant of protein mobility and rigidness was also analyzed, and it demonstrates that there is less fluctuation in proteins predicted by homology modeling compared to the structures of the *ab initio* model. Protein structures of homology modeling follow a constant trend over the 50 ns trajectory. Furthermore, we have also determined the number of hydrogen bonds that is an important determinant of a stable structure complex. The protein structures predicted by homology modeling follow a stable hydrogen bonding over the 50 ns trajectory without no significant fluctuations. Overall, NP_249450.1 and NP_251676.1 protein structures predicted from the SWISS-MODEL have a consistent and stable profile over the 50 ns simulation trajectory than the other model. The molecular dynamic simulation result is given in supplementary figure 2 and 3.

### 3.12. Active Site Identification

**Figure 8:**
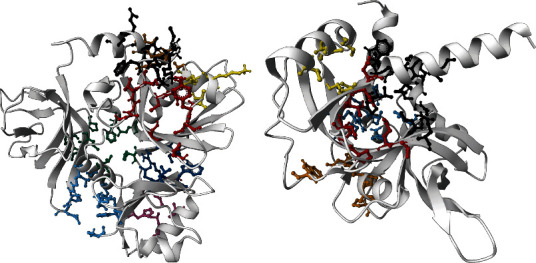
Active site prediction by Prank Web. (a) NP_249450.1 contained 8 binding pockets and different color showed different pockets (black for pocket 1; red: pocket 2; yellow: pocket 3; brown: pocket 4; blue: pocket 5; green: pocket 6; deep blue: pocket 7; and pink color for pocket 8). (b) NP_251676.1 showed 5 probable pockets which were designated by different colors (black for pocket 1; red: pocket 2; yellow: pocket 3; brown: pocket 4; and blue: pocket 5).

For the prediction of active sites, we selected the SWISS-MODEL structures for both proteins on basis of the evaluation of the simulation result. Prank Web provides existing pockets of protein with a pocket score and probability score. According to the database, the SWISS-MODEL structure of NP_249450.1 contained 8 pockets: binding pocket 1 (pocket score: 4.74, probability score: 0.220, AA count: 8); pocket 2 (pocket score: 3.15, probability score: 0.110, AA count: 11); pocket 3 (pocket score: 2.58, probability score: 0.075, AA count: 8); pocket 4 (pocket score: 2.38, probability score: 0.063, AA count: 8); pocket 5 (pocket score: 2.16, probability score: 0.050, AA count: 9); pocket 6 (pocket score: 1.42, probability score: 0.018, AA count: 10); pocket 7 (pocket score: 1.00, probability score: 0.007, AA count: 7); and pocket 8 (pocket score: 0.98, probability score: 0.006, AA count: 7). On the other hand, the SWISS-MODEL structure of NP_251676.1 possessed 5 binding pockets: binding pocket 1 (pocket score: 4.21, probability score: 0.184, AA count: 13); pocket 2 (pocket score: 3.16, probability score: 0.110, AA count: 11); pocket 3 (pocket score: 1.53, probability score: 0.022, AA count: 7); pocket 4 (pocket score: 1.39, probability score: 0.017, AA count: 7); and pocket 5 (pocket score: 1.21, probability score: 0.012, AA count: 9). Both the proteins with binding residue are shown in Figure 8.

## 4. Discussion

*Pseudomonas aeruginosa* PA01 is an omnipresent pathogenic bacterium that can cause acute and chronic infection in humans by contaminating environmental water and food, daily food spoilage, and infections. It is a rising concern for its increasing resistance against a broad range of antimicrobials. The biofilm-forming ability and evolution of antibiotic tolerance shapes *pseudomonas* isolate highly resistant against imipenem (95.3%), trimethoprim-sulfamethoxazole (69.8%), aztreonam (60.5%), chloramphenicol (45.3%), and meropenem (27.9%) [69]. Factors like chromosomal mutations and transferring of resistant genes through horizontal gene transfer contribute to its broad-spectrum drug resistance property [70]. Thus, it is necessary to introduce a new drug target when multidrug resistance for any disease or problem is noticed. *In silico* process has a great advantage for the identification of new drug targets in that situation within a very short time. Consequently, to combat the ever-increasing danger of antibiotic resistance, identifying novel drug targets is a dire necessity. A drug target should have some properties before it is considered a new target which includes being nonhomologous to human proteome, human antitargets, human gut microbiota, virulence capability, and druggability. For this reason, we scrutinized the properties of our targeted essential hypothetical proteins where analyzing these EHPs from multidrug resistance bacteria can lead to the identification of new potential therapeutic solutions.

We searched for essential hypothetical proteins (EHPs) among the 336 essential proteins of this bacterial strain to meet this need. Essential genes/proteins are those that are vital for a pathogen's survival and thereby analyzing their functions and metabolic pathways, and crucial information that may be central to life can be retrieved. In this research, we discovered 18 EHPs for the first time that may provide valuable information about the pathogenesis, molecular mechanisms, and functions of these bacteria. Functional annotation is a prerequisite in understanding the pathogen metabolic pathways and the products that they synthesize for their survival in adverse conditions. Moreover, domain analysis, which is a basic, distinctive, and stable unit of a protein structure that is fiercely conserved during the evolutionary process, is crucial for further investigation [71]. Moreover, the function of a protein is directly or indirectly related to subcellular localization [72]. The physicochemical properties of a protein depict a chemical assessment that shows the identity of chemical nature and physical hazards and to understands or predicts molecular attributes. The combined analysis of the physicochemical properties helps to characterize the proteins annotated as hypothetical proteins from the genome of an opportunistic pathogen like *P. aeruginosa* PAO1 (Table 3) [73].

In this study, the PPI network has provided congruent meaningful insights into the protein's function. Here, we have looked for potential relativity to our predicted function of EHPs and their connectivity with proteins involved with functionally important activities. The protein PA2986 (NP_251676.1) related to the MacB-like periplasmic core domain represents a connection with 45 proteins of which 8 are hypothetical proteins (HPs). A notable number of proteins grouped with PA2986 are involved in protein translocation activities such as translocation protein TolQ, TolR, and TolB. Tol proteins show activity in Gram-negative bacteria by providing stability to the outer membrane [74]. Moreover, the ABC transporter ATP-binding protein (PA0073) is related to it as it functions by utilizing TolC exit duct by shifting substrates to extracellular space from the periplasm [75]. This finding supports the idea of PA2986 being a member of the MacB-like periplasmic core domain. Another important protein for bacterial survival lysS, a lysine tRNA ligase was found to interact with PA2986 which is a mutant in some Gram-negative bacteria conferring resistance against the OP0595, diazabicyclooctane b-lactamase inhibitor (an antibiotic) [76]. We have found penicillin-binding protein 1 called ponA protein in this group of networks. Alteration in the ponA protein (penicillin-binding protein 1A) has a significant role in harnessing chromosomally mediated resistance against penicillin in *N. gonorrhoeae* [77]. Similarly, other considerable proteins like outer-membrane lipoprotein carrier protein lolA, transporter ExbB, and penicillin-binding protein 1A (ponA) interacted with PA2986 Figure 3.

PA0759 (NP_249450.1) has got the 2nd largest degree value having 41 nodes (Table 4) in connection of which 17 are HPs. The highest betweenness centrality value of 17881.76 determines its significance towards cell signaling pathways as in the case of directed or regulated networks. Betweenness centrality is considered a much robust essentiality indicator than degree value [78]. Genes in this hub include proteins having prime roles in translational regulation and cellular metabolic activities such as glycine cleavage system protein T2 [79], translation elongation factor (tsf) [80], and ribosomal large subunit pseudouridine synthase C (rluC) [81], respectively. Moreover, the RecO protein in this network is a replication-repairing protein from the RecF recombination repair pathway that facilitates both DNA strand annealing and DNA recombination in complex with RecA protein found in high radiation tolerant bacteria *Deinococcus radiodurans* [82]. This property may also contribute to better survival efficacy for *P. aeruginosa* PA01 in extreme conditions.

The PA4562 (NP_253252.1) protein is a probable member of the Lipid II flippase MurJ family which is used for the genesis of lipid II on both inner and outer leaflets and ultimately produces peptidoglycan in almost every bacterial species. Peptidoglycan is the primary protective foundation for shielding against environmental hazards and is involved in cell wall organizations [83]. Proteins related with morphological importance in bacteria such as flagellar basal body rod protein (FlgC) [84], rod shape-determining protein (rodA) [85], and type 4 fimbrial biogenesis outer-membrane protein (PilQ) [86] are present in a connection with the PA4562 proteins that strengthen our prediction regarding this protein function.

The proteins PA3685 (NP_252375.1) and PA3767 (NP_252456.1) are adjoined with 12 and 6 HPs, respectively. Both of these proteins are largely involved with enzymes of different molecular functions—tRNA N6-adenosine threonyl carbamoyl transferase (gcp) is a universal structural modifier found at position 37 of tRNAs that provides the anticodon loop with greater binding efficiency to ribosomes *in vitro* in *E. coli* [87]. The protein UDP-2,3-diacyl glucosamine hydrolase (PA1792) is hypothesized to be catalyzing lipid-A biogenesis in *E. coli* bacteria [88]. Lipid-A is a saccharolipid that modulates lipopolysaccharide (LPS) anchorage on the outer leaflet of the outer membrane in Gram-negative bacteria which is an essential component for the bacteria shielding from antibiotics and sustaining its viability [89]. Besides, other proteins having enzymatic properties include thiamine monophosphate kinase (thiL), ATP-dependent DNA helicase DinG (PA1045), riboflavin-specific deaminase/reductase (ribD), amidotransferase(PA1742), and acetyltransferase (PA2631) Figure 3.

The majority of the proteins connected with PA4992 (NP_253679.1) from aldo/keto reductase family are uncharacterized proteins. The PA4167 protein has a contributing role as a source of carbon and energy for a large number of bacteria [90]. The hub proteins PA4093 (NP_252782.1) and PA4992 (NP_253679.1) are interconnected through the intermediate protein PA4098, a probable short-chain dehydrogenase enzyme [91].

The mgtE is an Mg transporter that represents a connection with the PA3481 (NP_252171.1) which can be an opportunistic inhibitor for the type III secretion system (T3SS). T3SS is a formidable toxin injected by *P. aeruginosa* that can ultimately cause cell death into its host. The mgtE interrupts the T3SS transcription regulation system by provoking rsmYZ gene transcription and hence inhibits T3SS protein expression [92]. Interestingly, another analogous protein, DNA polymerase II (polB), is associated with this same hub protein PA3481. polB functions as a crucial candidate for repressing the translation process of master T3SS regulator ExsA. ExsA operates a major role in maintaining the regulatory cascade of T3SS. Thus, affecting ExsA expression can prohibit T3SS toxin secretion process. Furthermore, Chakravarty et al. found that T3SS transcription is attenuated when polB is overexpressed. Therefore, polB may act as a promising target for therapeutic interventions [93]. Besides, proteins responsible for exopolysaccharide biosynthesis and biofilm formation namely pslA [94] and pslD [95] are both members of the psl operon. The presence of such virulent protein types in this protein hub suggests PA3481 as a crucial protein involved in multiple virulence pathways in *P. aeruginosa*. The PA4636 (NP_253326.1) protein harbors some of the virulent proteins like lptA and algQ that are required for the biogenesis of lipid bilayer in the outer membrane in *P. aeruginosa* [96] and facilitates in developing a chronic infection in cystic fibrosis [97].

Some notable mutual interactions also have been observed between two hub proteins like PA2986 and PA4562 where interrelated proteins include mraY, a potential target for antibiotic development that is a crucial element for the bacterial cell wall synthesis [98], and opr86, an outer-membrane protein found previously in all Gram-negative bacteria. Likewise, it is suggested as a potential drug target with a significant therapeutic potential against *P. aeruginosa* in earlier studies [99]; rpoH, a 32 kDa heat shock protein in *E. coli*, can also take part as a complementary for sigma factor during the increasing temperature in the environment as well as while starving [100]; PA5568 possesses an inner membrane translocation subunit protein YidC which facilitates proteins to be passed onto inner membranes without the help of Sec translocase complex proteins [101]; ComL is a lipoprotein that facilitates the DNA transformation process in *N. gonorrhoeae* [102]. Lastly, organic solvent tolerance protein OstA holds interaction simultaneously with the top 3 hub genes of maximum node connection. Concurrently, OstA is a protein of high molecular significance as it is found in almost all Gram-negative bacteria and is involved in the bacterial envelope biogenesis process. A study by Chiu et al. found that OstA deficiency in *Helicobacter pylori* causes sensitivity to organic solvents, impaired membrane permeability, and vulnerability to antibiotics [103]. The function of the proteins in this network shows relational integrity with our predicted HPs. Knowing the protein's function in a protein-protein interaction network can facilitate the process of discovering the proteins with unknown functions [104].

Herein, we analyzed these properties of our targeted essential hypothetical proteins where PBIT servers direct categorized all the nonhomology features (Table 5). We have selected NP_249450.1 and NP_251676.1, respectively, for being virulent determined by two of our tools with strong confidence scores. Targeting these virulent factors can limit the pathogenicity of *P. aeruginosa*. Even antivirulence drugs insist a pathogen towards a weaker selection for resistance in them compared to antibiotics [11]. Therefore, understanding the virulence factors and their role in pathogenesis can lead us to a new potential therapeutic solution. Besides, druggability analysis also confirmed that NP_249450.1 and NP_251676.1 can be a new and potential drug target.

Structure prediction and quality assessment of the predicted structure are also parallelly important to evaluate the molecular and biological functions of a protein in cells for in-depth analysis and drug target identification [105]. Before structure prediction, the information of Alpha helix (Hh), extended strand (Ee), beta turn (Tt), or random coil (Cc) helps to establish the secondary structure. That is why the secondary structure was annotated to complete the structure related to all the information of our selected two proteins (Figures 4 and 5, and supplementary table 3). Thus, we further predicted the 3D structure of two EHPs (NP_249450.1 and NP_251676.1) and assessed the quality of these structures to decipher their unique conformation. The 3D structure and Ramachandran plot are depicted in Figures 6 and 7, respectively. The predicted structure's quality assessment parameters are listed in Table 6, and the overall quality factor is shown in Supplementary Figure 1. Our predicted structure is accurate and reliable, according to the Ramachandran plot analysis, since more than 90% of residues are considered the cutoff value and our findings surpass that range by more than 94%.

Moreover, from the analysis of molecular dynamic simulation, NP_249450.1 and NP_251676.1 protein structures predicted from the SWISS-MODEL have a consistent and stable profile over the 50 ns simulation trajectory than the other model (Supplementary Figure 2 and 3). Based on the simulation result, active sites were identified. Besides, for the establishment of the target proteins as a therapeutic target, active sites needed to be predicted to find out where a ligand compound can probably bind and initiate respective reactions. The structures obtained from the SWISS-MODEL were selected for the active site prediction where NP_249450.1 and NP_251676.1 showed 8 and 5 probable binding pockets, respectively (Figure 8). Therefore, this structural and functional information will open a new window for further identification of potential drug candidates that can halt the surge of this pathogenic bacterium from becoming resistant.

## 5. Conclusion

Unveiling the functional characterization of pathogenic microorganisms is of great importance in biological processes and medical science. Essential proteins and essential hypothetical proteins are versatile macromolecules that can be crucial in inferring new treatment strategies towards these pathogenic bacteria. For the functional characterization of EHPs, we used an *in silico* approach in combination with different bioinformatics databases/tools, with ROC analysis indicating that these tools are highly reliable for the functional characterization of *P. aeruginosa* PA01. We attributed function to 18 EHPs and analyzed the subcellular localization and physiochemical properties of these proteins. Afterwards, a PPI network analysis was carried out on 9 stable EHPs and their functionally related proteins from this bacterium. Further, host nonhomologous analysis predicts 5 pathogen-specific proteins, three of which have virulent factors that could be used as novel therapeutic targets. Finally, the structural conformation of two EHPs (NP_249450.1 and NP_251676.1) was determined, and the accuracy of the predicted model was evaluated, indicating that this model is highly accurate. Our findings will pave the way for new antibacterial drugs and treatment strategies to be developed by focusing on these novel drug targets.

## Figures and Tables

**Table 1:** Bioinformatics resources used in the study.

Serial No.	Server/database	Version	Using reason	Link	References
Functional annotation					
1	DEG	15.2	Finding essential HPs	http://tubic.tju.edu.cn/deg/	[12]
2	GO FEAT	1.0	For functional annotation	http://computationalbiology.ufpa.br/gofeat/	[13]
3	CDART		Protein homology search domain architecture	https://www.ncbi.nlm.nih.gov/Structure/lexington/lexington.cgi	[14]
4	SMART		Identification and annotation of protein domains	http://smart.embl-heidelberg.de/	[15]
5	SUPERFAMILY	1.75	For functional annotation	https://supfam.mrc-lmb.cam.ac.uk/SUPERFAMILY/	[16]
6	Pfam	34.0	Determine protein families	http://pfam.xfam.org/	[17]
7	SVMProt		Protein functional family prediction	http://bidd.group/cgi-bin/svmprot/svmprot.cgi	[18]
8	CATH	4.3	Protein domains into superfamily	http://www.cathdb.info/	[19]
9	InterPro	84.0	Classification of protein families	https://www.ebi.ac.uk/interpro/	[20]
10	HHPred		Sequence similarity searching, prediction of sequence features, and sequence classification.	https://toolkit.tuebingen.mpg.de/tools/hhpred	[21]
11	PANNZER		Functional annotation of uncharacterized proteins	http://ekhidna2.biocenter.helsinki.fi/sanspanz/	[22]
12	PFP		Automated protein function Gene Ontology prediction	https://kiharalab.org/web/pfp.php	[23]
13	ESG		Protein function prediction	https://kiharalab.org/web/esg.php	[24]
Subcellular localization					
14	Psortb	3.0.2	Subcellular localization	https://www.psort.org/psortb/	[25]
15	CELLO	v.2.5	Subcellular localization	http://cello.life.nctu.edu.tw/	[26, 27]
16	TMHMM	v. 2.0	Prediction of transmembrane helices in proteins	http://www.cbs.dtu.dk/services/TMHMM-2.0/	[28]
17	Phobius		Prediction of transmembrane helices in proteins	https://phobius.sbc.su.se/index.html	[29]
18	HMMTOP	2.0	Prediction of transmembrane helices in proteins	http://www.enzim.hu/hmmtop/index.php	[30]
19	CCTOP	1.00	Prediction of transmembrane helices in proteins	http://cctop.enzim.ttk.mta.hu/	[31]
20	PROTTER	1.0	Predicts the presence and location of signal peptide cleavage sites in amino acid sequences and prediction of transmembrane helices in proteins	https://wlab.ethz.ch/protter/start/	[32]
21	SignalP 4.1	4.1	Predicts the presence and location of signal peptide cleavage sites in amino acid sequences	http://www.cbs.dtu.dk/services/SignalP-4.1/	[33]
22	PrediSi		Prediction of signal peptides	http://www.predisi.de/	[34]
Physicochemical properties					
23	ProtParam		Computation of various physical and chemical parameters for a given protein	https://web.expasy.org/protparam/	[36]
Protein-protein interaction					
24	NetworkAnalyst	v3.0	PPI construction and visualization	https://www.networkanalyst.ca/NetworkAnalyst/uploads/ListUploadView.xhtml	[39]
Nonhomology analysis					
25	PBIT		Pipeline building for nonhomology analysis	http://www.pbit.bicnirrh.res.in/	[41]
Virulence factor analysis					
26	VICMpred		Functional classification of proteins of bacteria into virulence factors	https://webs.iiitd.edu.in/raghava/vicmpred/index.html	[43]
27	VirulentPred		VirulentPred is a bacterial virulent protein prediction method	http://bioinfo.icgeb.res.in/virulent/	[44]
28	MP3		Predict pathogenic proteins in both genomic and metagenomic datasets	http://metagenomics.iiserb.ac.in/mp3/tutorial.php	[45]
Druggability analysis					
29	DrugBank	5.0	Identification of information on drugs and drug targets	https://go.drugbank.com/	[47]
Secondary structure analysis					
30	SOPMA		Secondary structure prediction	https://npsa-prabi.ibcp.fr/cgi-bin/npsa_automat.pl?page=/NPSA/npsa_sopma.html	[48]
31	PSIPRED	4.0	Secondary structure prediction	http://bioinf.cs.ucl.ac.uk/psipred/	[49]
3D structure analysis					
32	SWISS-MODEL		Protein 3D structure determination	https://swissmodel.expasy.org/	[50]
33	Robetta		Protein 3D structure determination	https://http://robetta.bakerlab.org/	
34	Galaxy refine		Refinement of protein structure	http://galaxy.seoklab.org/cgi-bin/submit.cgi?type=REFINE	[52]
35	PyMOL software	2.0	Structure visualization	https://pymol.org/2/	
Validation check					
36	ERRAT		3D structure validation	https://saves.mbi.ucla.edu/	[53]
37	VARIFY 3D		3D structure validation	https://saves.mbi.ucla.edu/	[54, 55]
38	PROVE		3D structure validation	https://saves.mbi.ucla.edu/	[56]
39	WHATCHECK		3D structure validation	https://saves.mbi.ucla.edu/	[57]
40	PROCHECK		3D structure validation	https://saves.mbi.ucla.edu/	[58]
41	Ramachandran plot		3D structure validation	http://services.mbi.ucla.edu/SAVES/Ramachandran/	[58]
42	ROC analysis		This web page calculates a receiver operating characteristic (ROC) curve from data	http://www.rad.jhmi.edu/jeng/javarad/roc/JROCFITi.html	

**Table 2:** Functional annotations of 18 essential hypothetical proteins.

Serial No.	RefSeq	Superfamily	Family	Gene Ontology		GO ID/database integration
				Biological process	Molecular function	
1	NP_252456.1	Cytidine deaminase-like	Deoxycytidylate deaminase-like	(1) tRNA wobble adenosine to inosine editing	(1) Hydrolase activity (2) Zinc ion binding (3) Catalytic activity (4) tRNA-specific adenosine 34-deaminase activity	(GO:0002100) (GO:0016787) (GO:0008270) (GO:0003824) (GO:0052717) Uniprot (W1MGT3) Interpro (W1MGT3) Interpro (IPR016192) Interpro (IPR002125) Interpro (IPR016193) Interpro (IPR028883) Pfam (PF14437) NCBI (532131853) EMBL (ATNK01000135)
2	NP_252782.1	Hotdog thioesterase/thiol ester dehydratase-isomerase	Thioesterase	(1) Histidine biosynthetic process	(1) Histidinol dehydrogenase activity (2) Zinc ion binding (3) NAD binding	(GO: 0000105) (GO: 0004399) (GO: 0008270) (GO: 0051287) Uniprot (A0A448BY09) Interpro (A0A448BY09) Interpro (IPR029069) Interpro (IPR006683) Pfam (PF03061) EMBL (LR134300)
3	NP_253095.1	Uncharacterized protein	Dna[CI] antecedent, DciA	(1) Protein dephosphorylation	(1) Zinc ion binding (2) Protein tyrosine/serine/threonine phosphatase activity	(GO: 0006470) (GO: 0008270) (GO: 0008138) Uniprot (Q9HW03) Interpro (Q9HW03) KEGG (pae: PA4405) KEGG GM (pae: PA4405) Interpro (IPR007922) Pfam (PF05258) NCBI(489212117) EMBL (AE004091)
4	NP_253252.1	MATE_like	Lipid II flippaseMurJ, polysaccharide biosynthesis C-terminal domain	(1) Cell wall organization (2) Peptidoglycan biosynthetic process (3) Regulation of cell shape	(1) Lipid-linked peptidoglycan transporter activity	(GO: 0071555) (GO: 0009252) (GO: 0008360) (GO: 0015648) Uniprot (W1MQM4) Interpro (W1MQM4) Interpro (IPR004268) Pfam (PF03023) NCBI (532135099) EMBL (ATNK01000069)
5	NP_253326.1	Glycerol-3-phosphate (1)-acyltransferase	Glycerol-3-phosphate (1)-acyltransferase	(1) D-Galacturonate catabolic process (2) D-Glucuronate catabolic process	(1) Transferase activity, transferring acyl groups	Uniprot (Q9HVF5) Interpro (Q9HVF5) KEGG (pae: PA4636) KEGG GM (pae: PA4636) Interpro (IPR002123) Pfam (PF01553) NCBI (489205664) EMBL (AE004091)
6	NP_253368.1	TonB-dependent receptor family	Energy transducer TonB	(1) Viral process	(1) GTP binding	(GO: 0016032) (GO: 0005525) Uniprot (Q9HVB6) Interpro (Q9HVB6) KEGG (pae: PA4679) KEGG GM (pae: PA4679) NCBI (489212281) EMBL (AE004091)
7	NP_249450.1	Folate-binding	Aminomethyl transferase folate-binding domain	(1) Iron-sulfur cluster assembly (2) Glycine decarboxylation via glycine cleavage system	(1) Aminomethyl transferase activity (2) Transaminase activity	(GO: 0019464) (GO: 0004047) (GO: 0008483) Superfamily (GO: 0016226) Uniprot (Q9I5H0) Interpro (Q9I5H0) KEGG (pae: PA0759) KEGG GM(pae:PA0759) Interpro (IPR029043) Interpro (IPR017703) NCBI (489205124) EMBL (AE004091)
8	NP_250659.1	Inhibitor_I78	Peptidase inhibitor I78 family	(1) Cell adhesion (2) Homophilic cell adhesion via plasma membrane adhesion molecules	(1) Calcium ion binding (2) Serine-type endopeptidase inhibitor activity	(GO: 0007155) (GO: 0007156) (GO: 0005509) (GO: 0004867) SMART Uniprot (Q9I2D5) Interpro (Q9I2D5) KEGG (pae: PA1969) KEGG GM (pae:PA1969) Interpro (IPR021719) Pfam (PF11720) NCBI (489210309) EMBL (AE004091)
9	NP_250846.1	DNase I-like	Endonuclease/exonuclease/phosphatase	N/A	(1) Endonuclease activity (2) Exonuclease activity	SMART (GO: 0004519) (GO: 0004527) Uniprot (A0A6N0KLP9) Interpro (A0A6N0KLP9) Interpro (IPR036691) Interpro (IPR005135) Pfam (PF03372) EMBL (CP054572)
10	NP_251676.1	LolE	MacB-like periplasmic core domain, lipoprotein-releasing ABC transporter permease	(1) Lipoprotein localization to outer membrane (2) Lipoprotein transport (3) Protein localization to outer membrane	(1) ATP binding (2) ATPase-coupled xenobiotic transmembrane transporter activity (3) Efflux transmembrane transporter activity (4) ATPase activity	SMART (GO: 0044874) (GO: 0042953) (GO: 0089705) (GO: 0005524) (GO: 0008559) (GO: 0015562) (GO: 0016887) Uniprot (Q9HZL8) Interpro (Q9HZL8) KEGG (pae:PA2986) KEGG GM (pae:PA2986) Interpro (IPR003838) Interpro (IPR011925) Interpro (IPR025857) Pfam (PF02687) Pfam (PF12704) NCBI (489210993) EMBL (AE004091)
11	NP_252171.1	Fe-S cluster assembly (FSCA) domain-like	Iron-sulfur cluster assembly protein	(1) Iron-sulfur cluster assembly	(1) ATPase activity (2) ATP binding (3) Iron-sulfur cluster binding (4) Metal ion binding	SMART (GO: 0016226) (GO: 0016887) (GO: 0005524) (GO: 0051536) (GO: 0046872) Uniprot (A0A3S4MTX6) Interpro (A0A3S4MTX6) Interpro (IPR034904) Interpro (IPR002744) Interpro (IPR019591) Interpro (IPR000808) Interpro (IPR027417) Interpro (IPR033756) Pfam (PF01883) Pfam (PF10609) EMBL (LR134300)
12	NP_252375.1	Carbam_trans_N (carbamoyltransferase N-terminus)	tRNA N6-adenosine threonyl carbamoyltransferase	(1) tRNA threonyl carbamoyl adenosine modification	(1) Metalloendopeptidase activity (2) Iron ion binding (3) N(6)-L-Threonyl carbamoyl adenine synthase activity	SMART (GO: 0002949) (GO: 0004222) (GO: 0005506) (GO: 0061711) Uniprot (Q9HXV5) Interpro (Q9HXV5) KEGG (pae:PA3685) KEGG GM (pae:PA3685) Interpro (IPR043129) Interpro (IPR000905) Interpro (IPR022496) Pfam (PF00814) NCBI (887492937) EMBL (AE004091)
13	NP_253374.1	MukE (MukE is part of the MukBEF condensin complex)	Bacterial condensin subunit MukE	(1) Cell cycle (2) Cell division (3) DNA replication (4) Chromosome segregation (5) Chromosome condensation	(1) GTP binding (2) GTPase activity (3) Translation elongation factor activity (4) ATP binding	(GO: 0007049) (GO:0051301) (GO:0006260) (GO:0007059) (GO:0030261) (GO:0005525) (GO:0003924) (GO:0003746) (GO:0005524) Uniprot (A0A448BSU5) Interpro (A0A448BSU5) Interpro (IPR042038) EMBL (LR134300)
14	NP_253434.1	(1) RimP N-terminal domain (2) RimP C-terminal SH3 domain (also known as yhbC)	RimP N-terminal domain, RimP C-terminal SH3 domain	(1) Ribosomal small subunit biogenesis	N/A	SMART (GO:0042274) Uniprot (A0A3S4MTG9) Interpro (A0A3S4MTG9) Interpro (IPR003728) Interpro (IPR028998) Interpro (IPR036847) Interpro (IPR028989) Interpro (IPR035956) Pfam (PF02576) Pfam (PF17384) EMBL (LR134300)
15	NP_253455.1	Bet v1-like	Polyketide cyclase/dehydrase and lipid transport	(1) Ubiquinone biosynthetic process (2) Cellular respiration	(1) Ubiquinone binding	(GO: 0006744) (GO: 0045333) (GO: 0048039) Superfamily 1.75 SMART IPR005031 Uniprot (A0A448BT54) Interpro (A0A448BT54) Interpro (IPR005031) Interpro (IPR023393) Pfam (PF03364) EMBL (LR134300)
16	NP_253678.1	FAD/NAD(P)-binding domain	FAD-dependent oxidoreductase	(1) Oxidation-reduction process	(1) Oxidoreductase activity	SMART (GO: 0055114) (GO: 0016491)
17	NP_253679.1	NAD(P)-linked oxidoreductase/ Aldo/keto reductase (AKR) superfamily	Aldo/keto reductase family	(1) Daunorubicin metabolic process (2) Doxorubicin metabolic process	(1) Oxidoreductase activity (2) D-Threo-aldose 1-dehydrogenase activity	SMART (GO:0044597) (GO:0044598) (GO:0047834) Uniprot (A0A3S4Q0Y1) Interpro (A0A3S4Q0Y1) Interpro(IPR023210) Interpro (IPR036812) Pfam (PF00248) EMBL (LR134300)
18	NP_253685.1	Protein kinase-like (PK-like)	Phosphotransferase enzyme family	(1) Protein phosphorylation	(1) ATP binding (2) Protein serine/threonine kinase activity	Pfam (GO:0006468) (GO:0005524) (GO:0004674) Uniprot (A0A3S5E573) Interpro (A0A3S5E573) Interpro (IPR011009) EMBL (LR134300)

**Table 3:** Physicochemical properties of 18 essential hypothetical proteins.

Serial No.	RefSeq	Molecular weight (Dalton)	Theoretical pI	Formula	Total number of negatively charged residues (Asp + Glu)	Total number of positively charged residues (Arg + Lys)	Instability index (II)	Aliphatic index	Grand average of hydropathicity (GRAVY)
1	NP_252456.1	19937.91	9.12	C_869_H_1409_N_265_O_255_S_9_	22	26	25.65 (stable)	83.13	-0.257
2	NP_252782.1	14871.24	7.93	C_658_H_1078_N_188_O_193_S_5_	15	16	37.46 (stable)	100.29	0.162
3	NP_253095.1	15057.34	10.71	C_657_H_1080_N_210_O_188_S_4_	12	21	57.65 (unstable)	93.28	-0.427
4	NP_253252.1	56122.54	10.03	C_2643_H_4201_N_651_O_651_S_19_	21	40	36.08 (stable)	133.96	0.857
5	NP_253326.1	43779.82	6.85	C_1955_H_3058_N_554_O_569_S_11_	51	50	38.61 (stable)	87.02	-0.376
6	NP_253368.1	24873.64	5.30	C_1111_H_1779_N_317_O_319_S_6_	28	25	73.37 (unstable)	91.89	-0.119
7	NP_249450.1	33667.59	5.37	C_1492_H_2415_N_425_O_446_S_7_	39	32	31.65 (stable)	108.22	0.057
8	NP_250659.1	13335.11	8.98	C_567_H_936_N_178_O_181_S_6_	12	15	53.34 (unstable)	83.54	-0.085
9	NP_250846.1	27693.92	9.90	C_1245_H_1962_N_380_O_330_S_5_	23	30	52.29 (unstable)	100.69	-0.229
10	NP_251676.1	47387.94	9.69	C_2139_H_3484_N_582_O_587_S_20_	34	44	38.17 (stable)	114.85	0.365
11	NP_252171.1	38888.77	5.26	C_1711_H_2780_N_482_O_517_S_16_	40	31	32.05 (stable)	102.34	0.090
12	NP_252375.1	24180.71	5.02	C_1081_H_1707_N_303_O_313_S_7_	27	19	29.03 (stable)	102.48	0.166
13	NP_253374.1	26354.58	4.52	C_1166_H_1811_N_315_O_366_S_8_	43	19	53.53 (unstable)	89.96	-0.366
14	NP_253434.1	17171.46	4.59	C_763_H_1208_N_206_O_236_S_4_	27	15	57.54 (unstable)	105.07	-0.197
15	NP_253455.1	16000.46	6.72	C_720_H_1130_N_190_O_208_S_7_	14	14	43.00 (unstable)	88.12	-0.037
16	NP_253678.1	42109.34	7.73	C_1866_H_3011_N_551_O_541_S_9_	47	48	48.61 (unstable)	98.72	-0.130
17	NP_253679.1	29030.07	6.00	C_1281_H_2067_N_373_O_386_S_5_	36	31	32.97 (stable)	101.22	-0.101
18	NP_253685.1	24985.76	9.60	C_1112_H_1791_N_337_O_311_S_4_	27	34	45.71 (unstable)	104.77	-0.365

**Table 4:** List of proteins with their reference sequence, Uniprot ID, protein name, node degree value, and betweenness centrality.

Serial No.	RefSeq	UniProt ID	Protein name	Degree	Betweenness centrality
1	NP_251676.1	Q9HZL8	PA2986	45	9681.83
2	NP_249450.1	Q9I5H0	PA0759	41	17881.76
3	NP_253252.1	Q9HVM2	PA4562	38	9315.74
4	NP_252375.1	Q9HXV5	PA3685	32	8742.58
5	NP_252456.1	Q9HXM8	PA3767	28	11489.25
6	NP_253679.1	Q9HUH3	PA4992	24	10103.17
7	NP_252171.1	Q9HYC8	PA3481	23	5329.08
8	NP_252782.1	Q9HWT5	PA4093	20	4750.0
9	NP_253326.1	Q9HVF5	PA4636	18	6466.58
10	NP_249286.1	Q9I5U2	ostA	3	10848.52
11	NP_250538.1	Q9I2P8	PA1847	3	5698.26

**Table 5:** Aspects of the proteins like nonhomology to human proteins and proteins of human gut flora, virulence of the pathogen, druggability for the 9 EHPs.

serial No.	Protein	Nonhomology analysis against human proteome	Nonhomology analysis against human antitargets	Nonhomology analysis against gut microbiota proteomes	Virulence analysis	Druggability analysis
1	NP_252456.1	Nonhomologous	Nonhomologous	Nonhomologous	Nonvirulent	Old target
2	NP_252782.1	Nonhomologous	Nonhomologous	Nonhomologous	Nonvirulent	Novel target
3	NP_253252.1	Nonhomologous	Nonhomologous	Homologous	Nonvirulent	Novel target
4	NP_253326.1	Nonhomologous	Nonhomologous	Nonhomologous	Nonvirulent	Novel target
5	NP_249450.1	Nonhomologous	Nonhomologous	Nonhomologous	Virulent	Novel target
6	NP_251676.1	Nonhomologous	Nonhomologous	Nonhomologous	Virulent	Novel target
7	NP_252171.1	Homologous	Nonhomologous	Nonhomologous	Nonvirulent	Novel target
8	NP_252375.1	Nonhomologous	Nonhomologous	Homologous	Nonvirulent	Novel target
9	NP_253679.1	Nonhomologous	Nonhomologous	Nonhomologous	Nonvirulent	Old target

**Table 6:** Three-dimensional structure validation of the predicted two hypothetical proteins from the SAVES v6.0 server.

Protein name	Saves result					
	ERRAT	VARIFY 3D	PROVE	WHATCHECK	PROCHECK	Ramachandran plot (% residue in the most favored region)
NP_249450.1 (SWISS-MODEL)	Overall quality factor 87.6325	97.70% of the residues have averaged 3D-1D score ≥ 0.2 Pass	Buried outlier protein atoms total From 1 model: 6.1% Fail	1234567891011 12131415161718 19202122232425 26272829303132 33343536373839 40414243444546	Out of 8 evaluations Errors: 3 Warning: 2 Pass: 3	94.6%
NP_249450.1 (Robetta)	Overall quality factor 92.459	94.90% of the residues have averaged 3D-1D score ≥ 0.2 Pass	Buried outlier protein atoms total From 1 model: 4.1% Warning	1234567891011 12131415161718 19202122232425 26272829303132 33343536373839 40414243444546	Out of 8 evaluations Errors: 3 Warning: 2 Pass: 3	94.0%
NP_251676.1 (SWISS-MODEL)	Overall quality factor 93.3649	82.59% of the residues have averaged 3D-1D score ≥ 0.2 Pass	Buried outlier protein atoms total From 1 model: 5.4% Fail	1234567891011 12131415161718 19202122232425 26272829303132 33343536373839 40414243444546	Out of 8 evaluations Errors: 2 Warning: 4 Pass: 2	95.3%
NP_251676.1 (Robetta)	Overall quality factor 98.063	66.97% of the residues have averaged 3D-1D score ≥ 0.2 Fail	Buried outlier protein atoms total From 1 model: 4.2% Warning	1234567891011 12131415161718 19202122232425 26272829303132 33343536373839 40414243444546	Out of 8 evaluations Errors: 2 Warning: 1 Pass: 5	97.3%

## Data Availability

The data used to support the findings of this study are included within the article.

## Supplementary Materials

Supplementary 1
*Overall quality factor of ERRAT value from SAVES v6.0 server.* (a) The quality factor is 87.6325% for the NP_249450.1 protein structure from SWISS-MODEL, (b) the quality factor is 93.3649% for NP_251676.1 protein structure from SWISS-MODEL, (c) the quality factor is 92.459% for the NP_249450.1 protein structure from Robetta, and (d) the quality factor is 98.063% for NP_251676.1 protein structure from Robetta.

Supplementary 2 Analysis of molecular dynamics simulation for the structures obtained from SWISS-MODEL (i) NP_249450.1 and (ii) NP_251676.1. (a) The RMSD values; (b) RMSF values; (c) the Radius of gyration (Rg) profile; (d) the hydrogen bond; and (e) SASA descriptors.

Supplementary 3 Analysis of molecular dynamics simulation for the structures obtained from Robetta (i) NP_249450.1 and (ii) NP_251676.1. (a) The RMSD values; (b) RMSF values; (c) the Radius of gyration (Rg) profile; (d) the hydrogen bond; and (e) SASA descriptors.

Supplementary 4 Table S1: subcellular localization and transmembrane topology. Table S2: list of functions of the proteins found in PPI network. Table S3: properties of secondary structure from SOPMA database.

Supplementary 5 ROC analysis.
