# Supplementary material for: Targeting Essential Hypothetical Proteins of Pseudomonas aeruginosa PAO1 for Mining of Novel Therapeutics: An In Silico Approach
Source: Biomed Res Int. 2023 Apr 11;2023:1787485. doi: 10.1155/2023/1787485 (PMC10119676; doi:10.1155/2023/1787485)
Supplement: Supplementary 1 — Overall quality factor of ERRAT value from SAVES v6.0 server. (a) The quality factor is 87.6325% for the NP_249450.1 protein structure from SWISS-MODEL, (b) the quality factor is 93.3649% for NP_251676.1 protein structure from SWISS-MODEL, (c) the quality factor is 92.459% for the NP_249450.1 protein structure from Robetta, and (d) the quality factor is 98.063% for NP_251676.1 protein structure from Robetta. [file 1787485.f1.pdf]

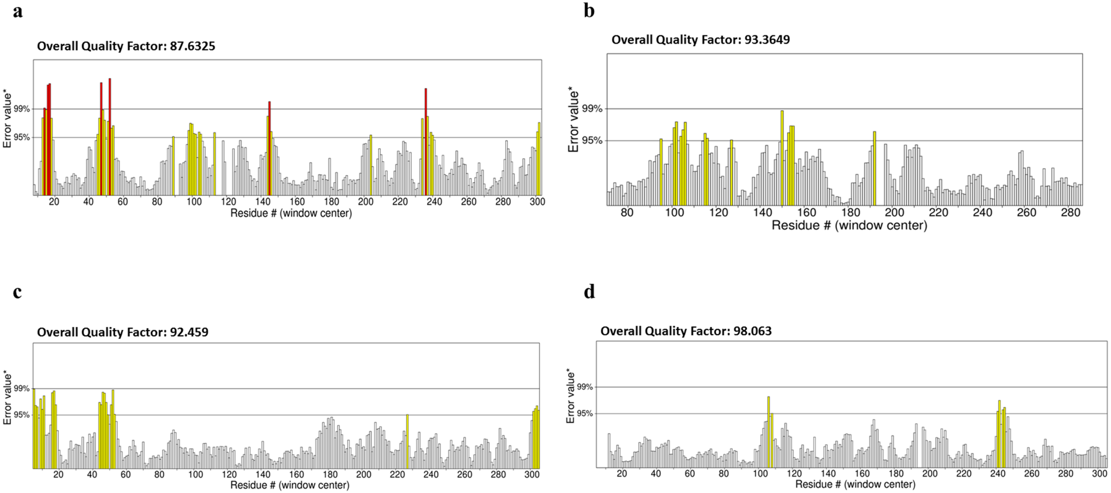
a
Overall Quality Factor: 87.6325
Error value*
99%
95%
20 40 60 80 100 120 140 160 180 200 220 240 260 280 300
Residue # (window center)
b
Overall Quality Factor: 93.3649
Error value*
99%
95%
80 100 120 140 160 180 200 220 240 260 280
Residue # (window center)
c
Overall Quality Factor: 92.459
Error value*
99%
95%
20 40 60 80 100 120 140 160 180 200 220 240 260 280 300
Residue # (window center)
d
Overall Quality Factor: 98.063
Error value*
99%
95%
20 40 60 80 100 120 140 160 180 200 220 240 260 280 300
Residue # (window center)
