# Supplementary material for: Targeting Essential Hypothetical Proteins of Pseudomonas aeruginosa PAO1 for Mining of Novel Therapeutics: An In Silico Approach
Source: Biomed Res Int. 2023 Apr 11;2023:1787485. doi: 10.1155/2023/1787485 (PMC10119676; doi:10.1155/2023/1787485)
Supplement: Supplementary 2 — Analysis of molecular dynamics simulation for the structures obtained from SWISS-MODEL (i) NP_249450.1 and (ii) NP_251676.1. (a) The RMSD values; (b) RMSF values; (c) the Radius of gyration (Rg) profile; (d) the hydrogen bond; and (e) SASA descriptors. [file 1787485.f2.pdf]

i

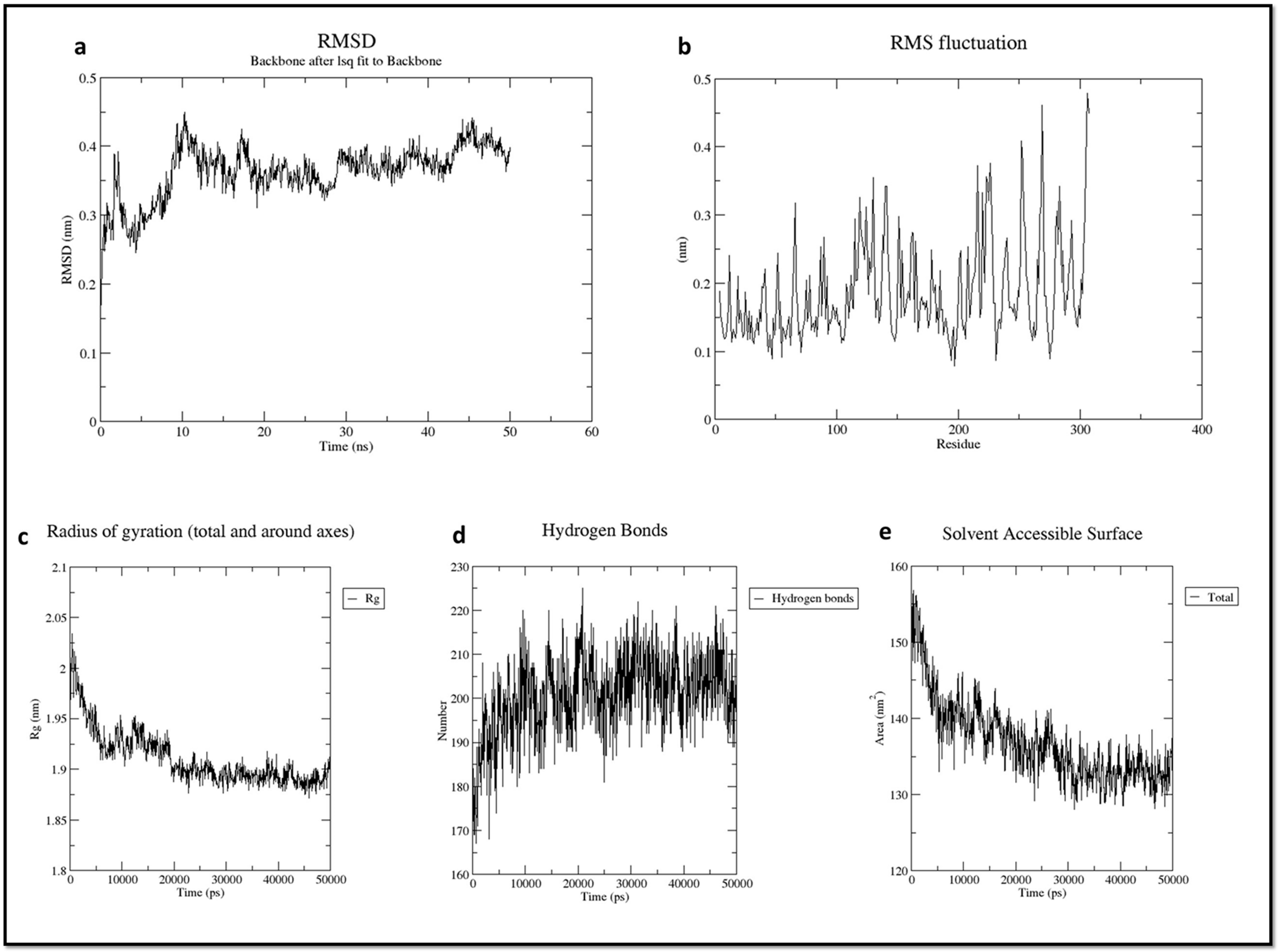
a
RMSD
Backbone after lsq fit to Backbone
RMSD (nm)
Time (ns)
b
RMS fluctuation
(nm)
Residue
c
Radius of gyration (total and around axes)
Rg
Rg (nm)
Time (ps)
d
Hydrogen Bonds
Hydrogen bonds
Number
Time (ps)
e
Solvent Accessible Surface
Total
Area (nm^2)
Time (ps)

ii

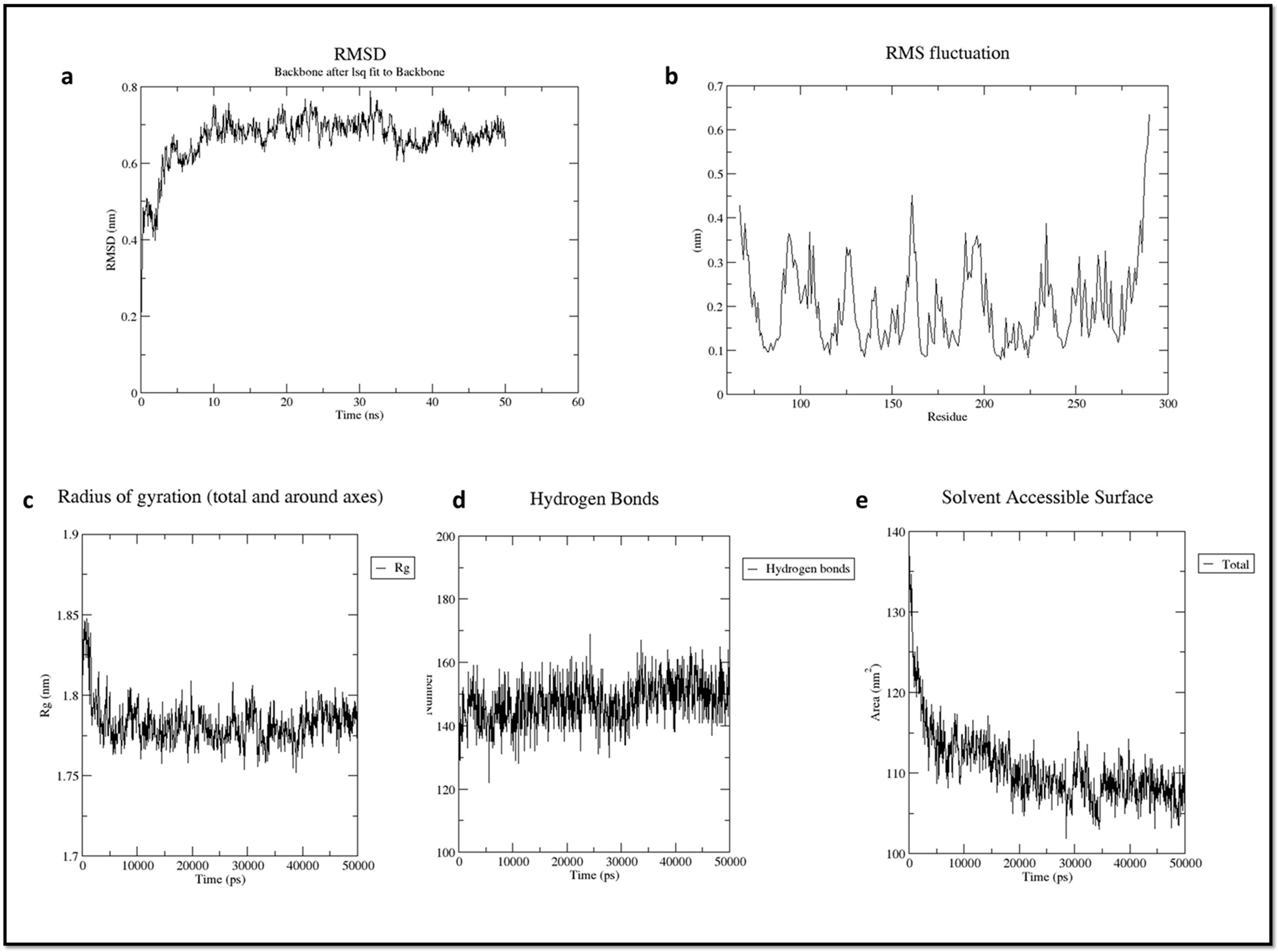
a
RMSD
Backbone after lsq fit to Backbone
RMSD (nm)
Time (ns)
b
RMS fluctuation
(nm)
Residue
c
Radius of gyration (total and around axes)
Rg
Rg (nm)
Time (ps)
d
Hydrogen Bonds
Hydrogen bonds
Number
Time (ps)
e
Solvent Accessible Surface
Total
Area (nm^2)
Time (ps)
