# Supplementary figures and images for: Targeting Essential Hypothetical Proteins of Pseudomonas aeruginosa PAO1 for Mining of Novel Therapeutics: An In Silico Approach
Source: Biomed Res Int. 2023 Apr 11;2023:1787485. doi: 10.1155/2023/1787485 (PMC10119676; doi:10.1155/2023/1787485)

i

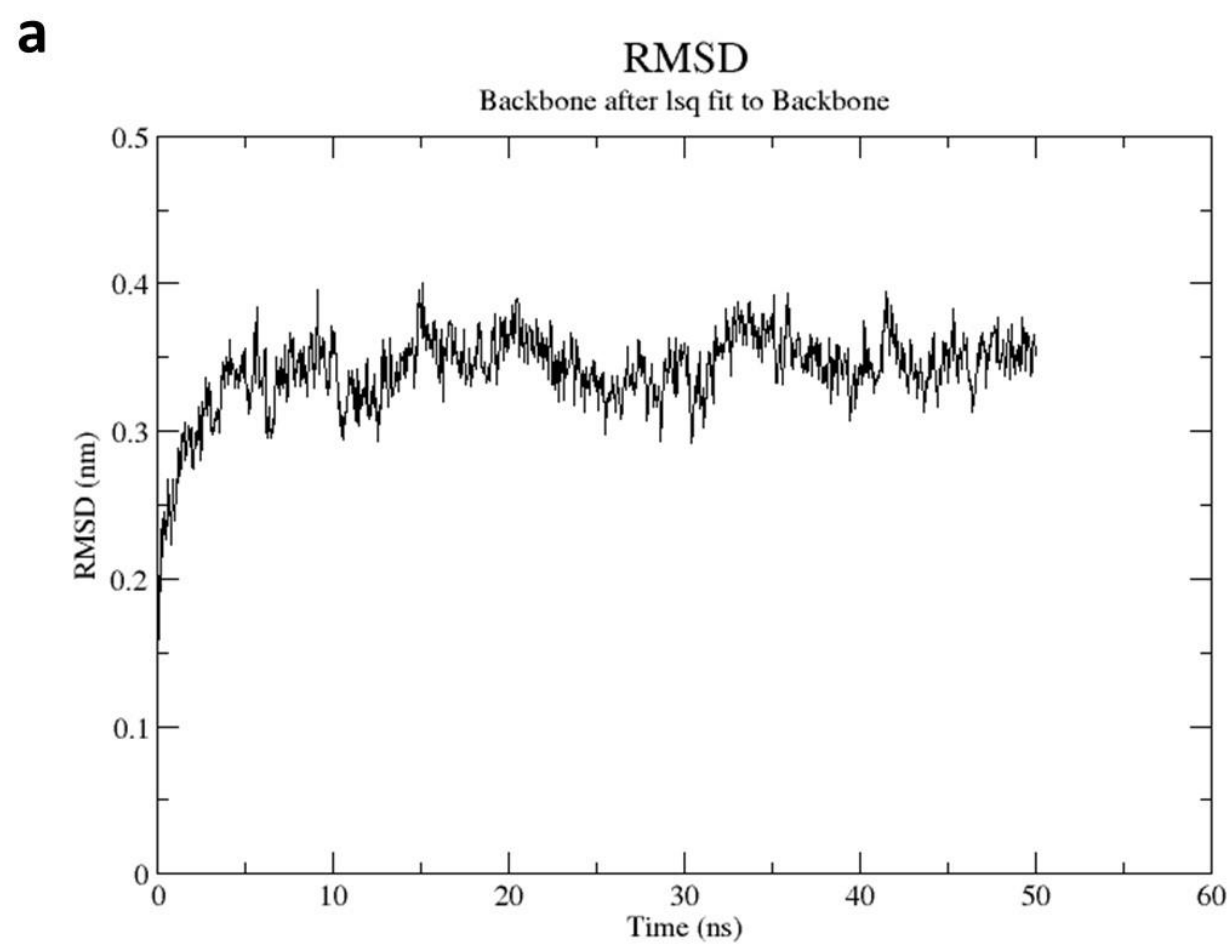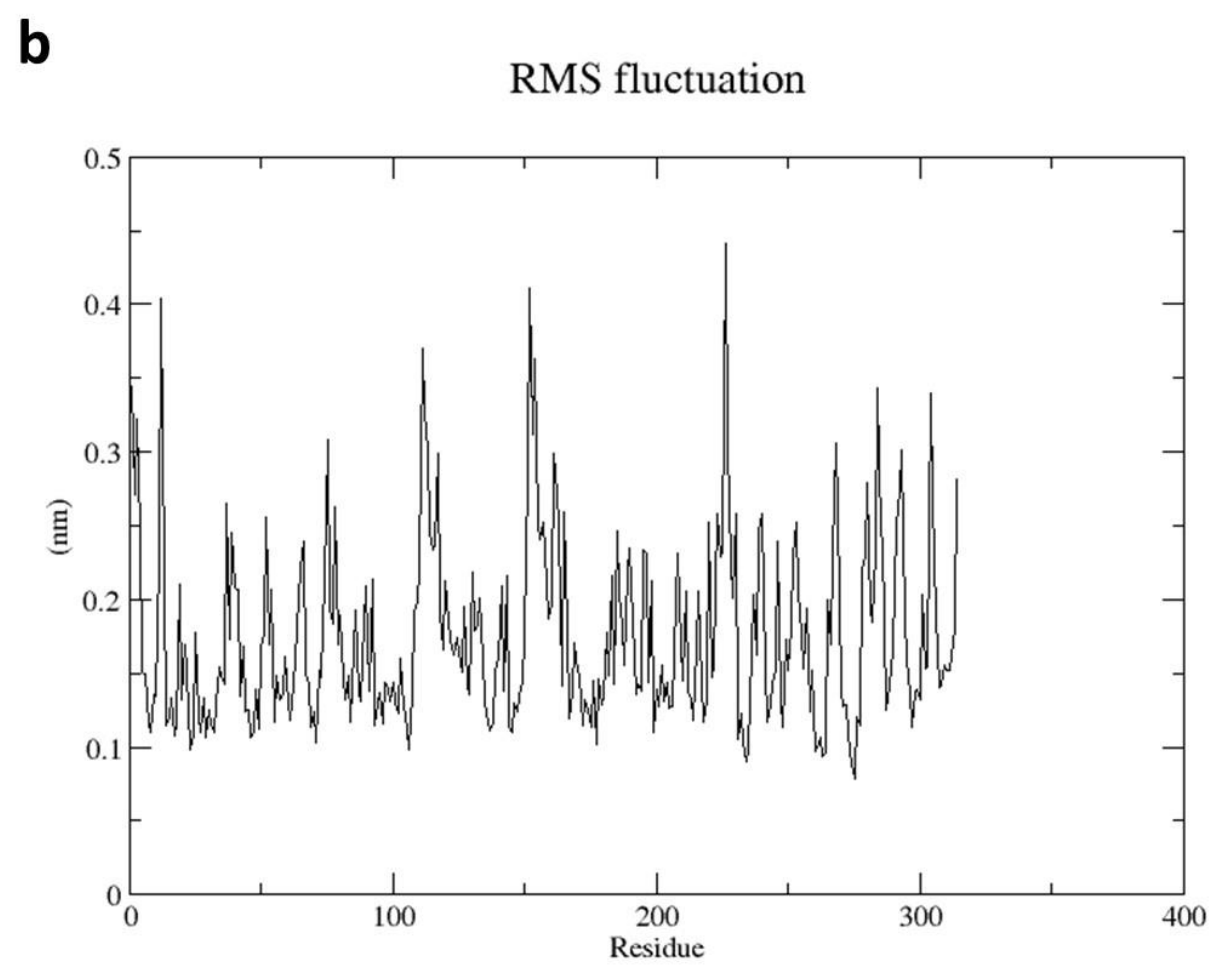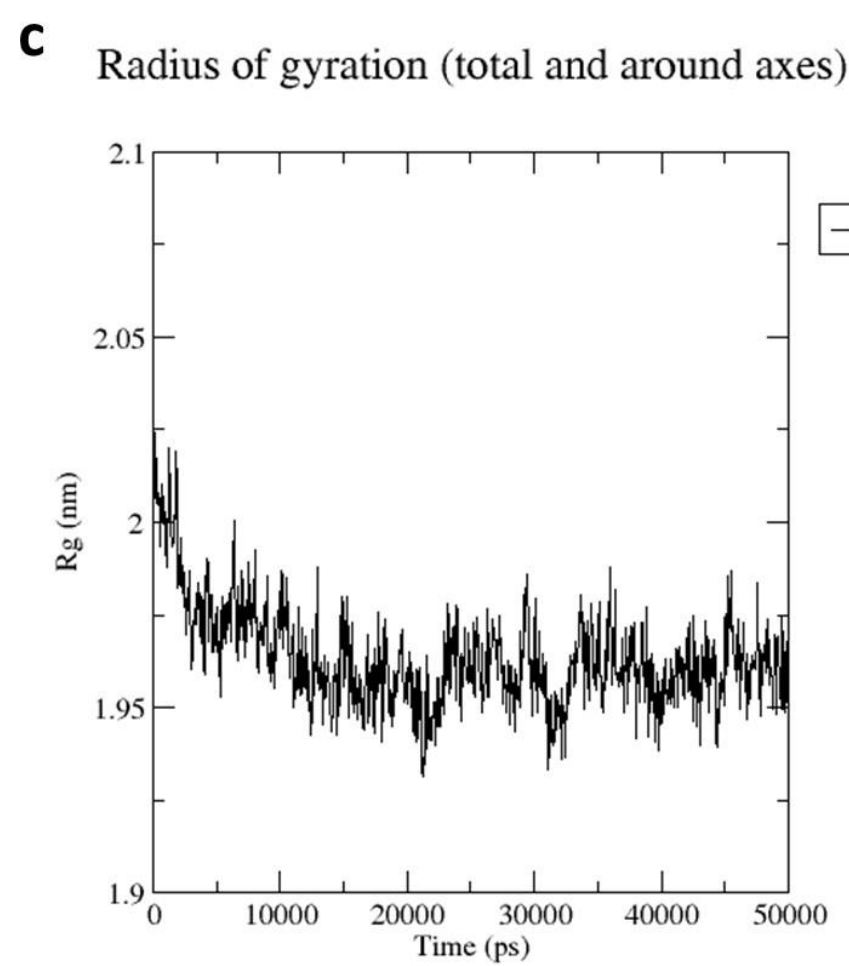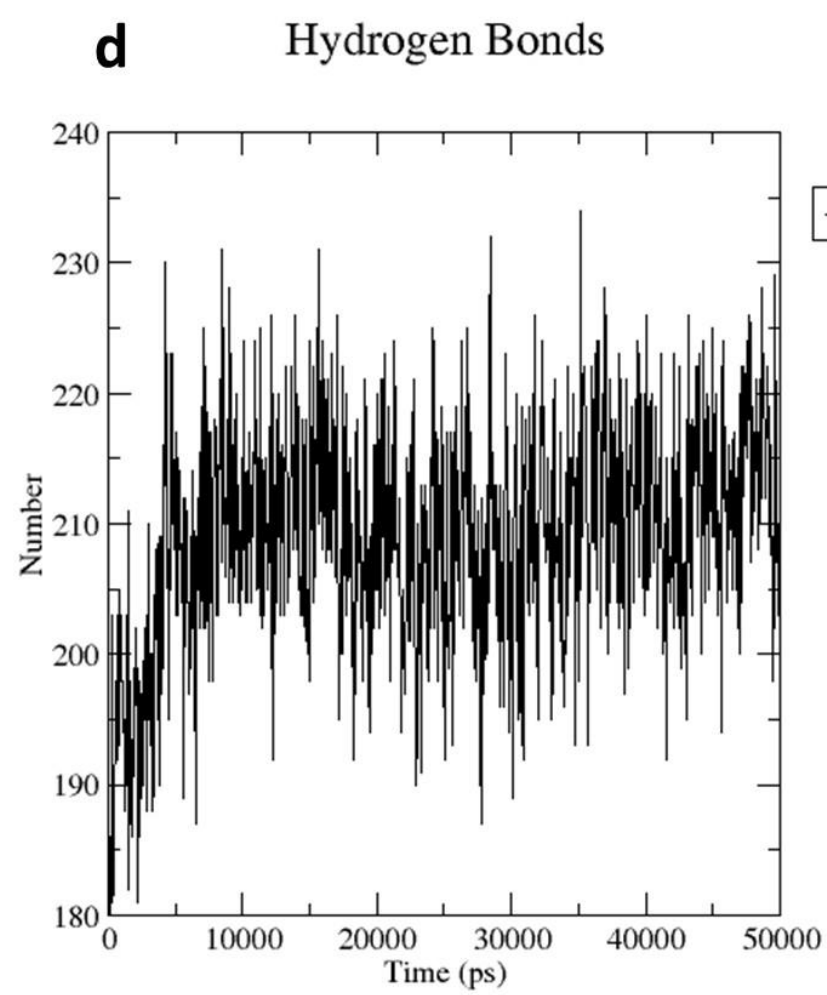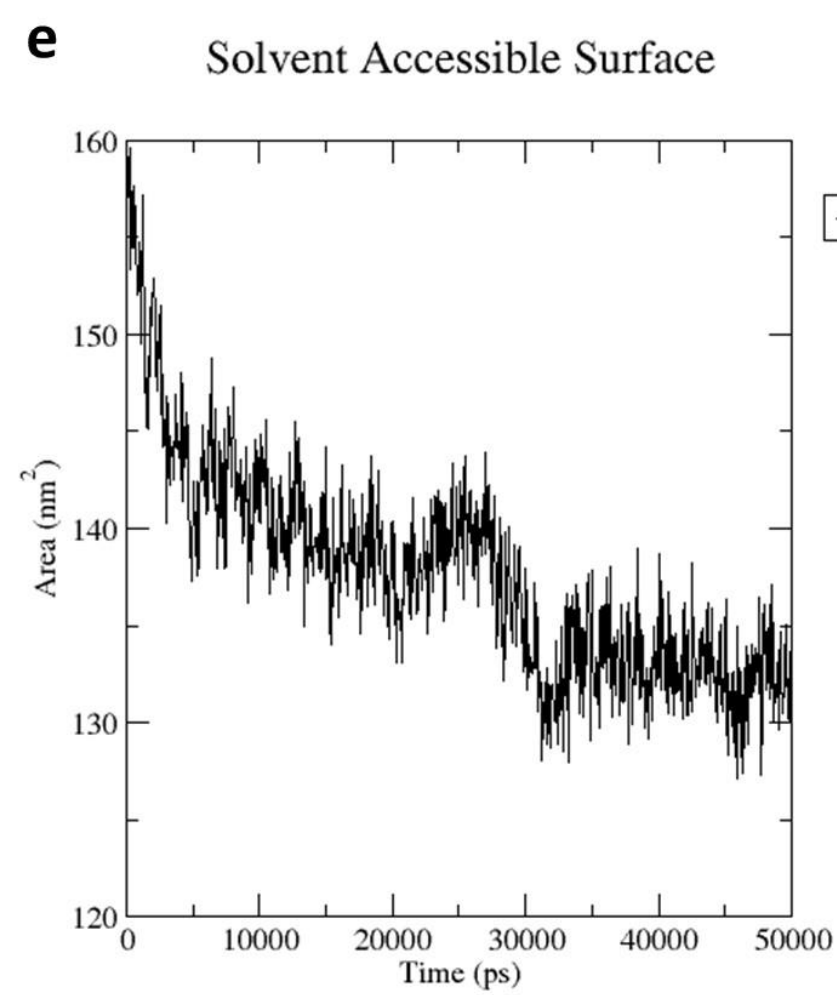

ii

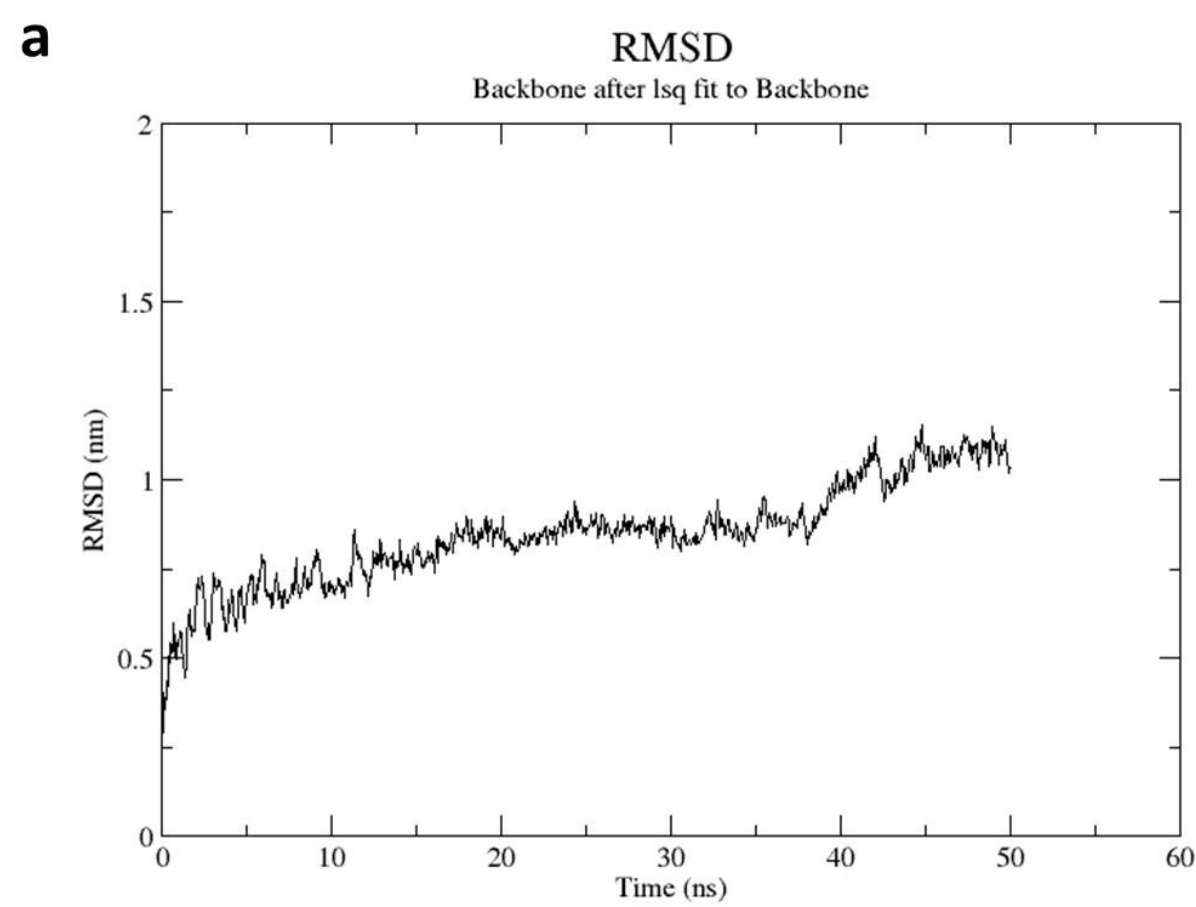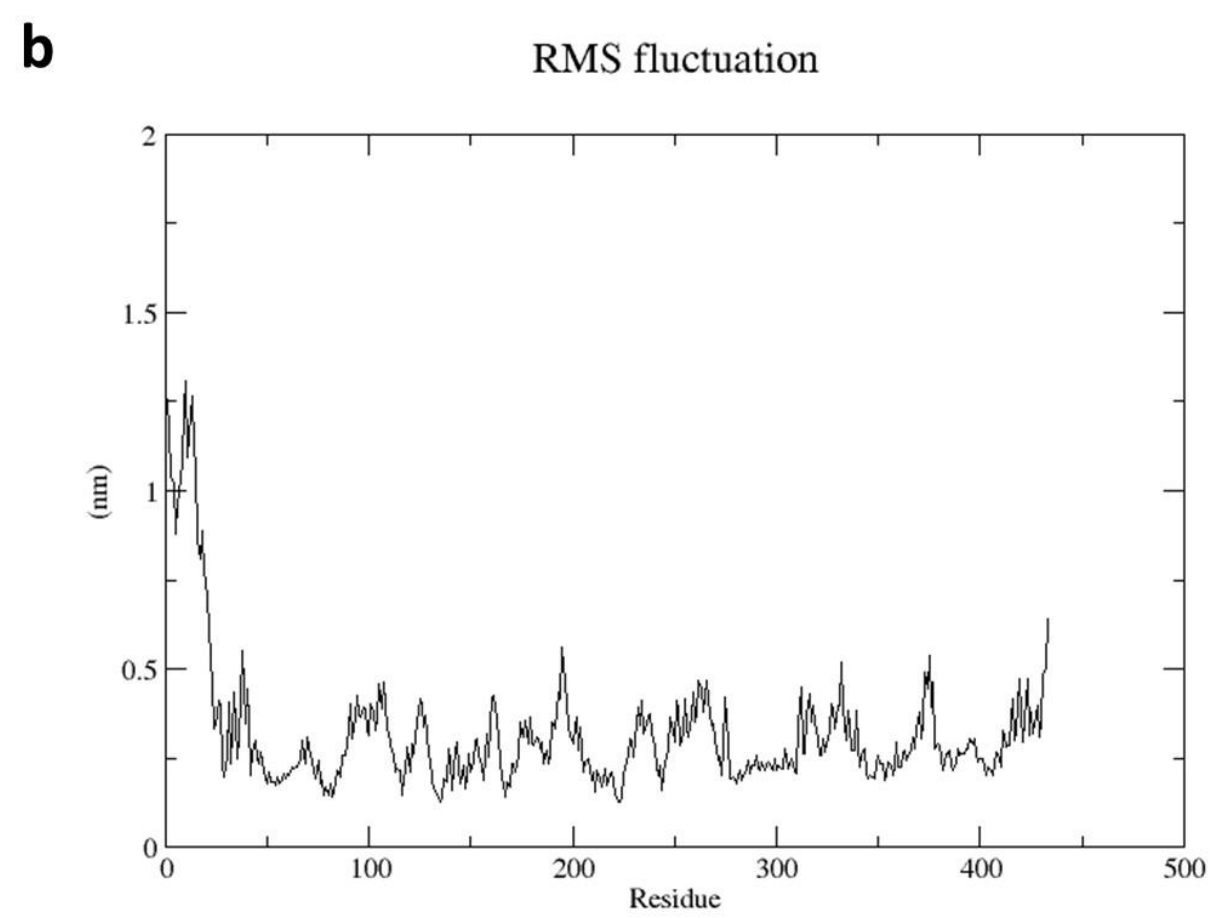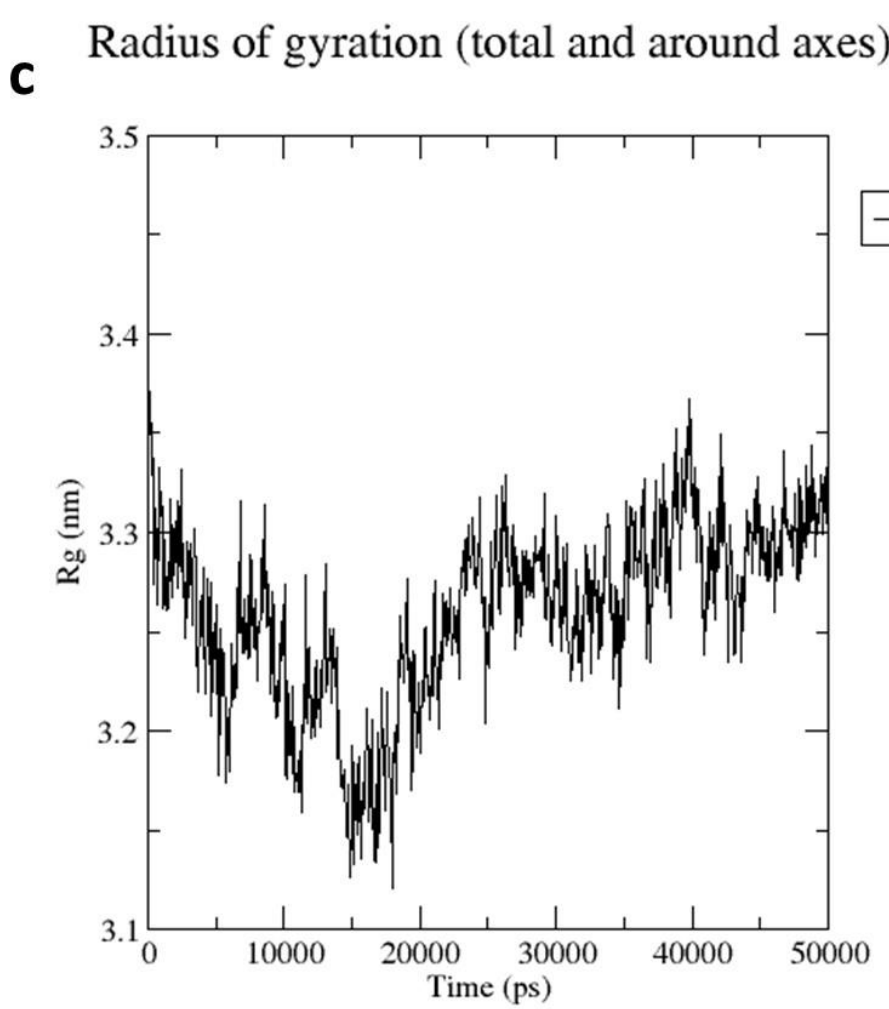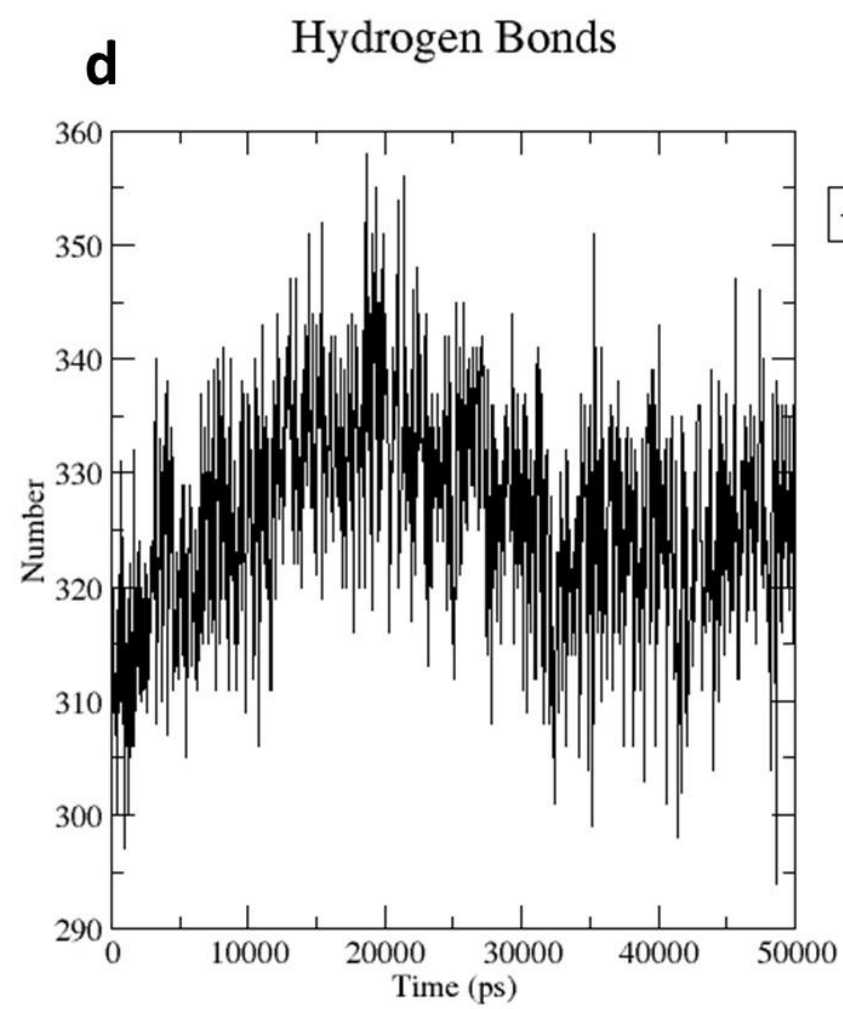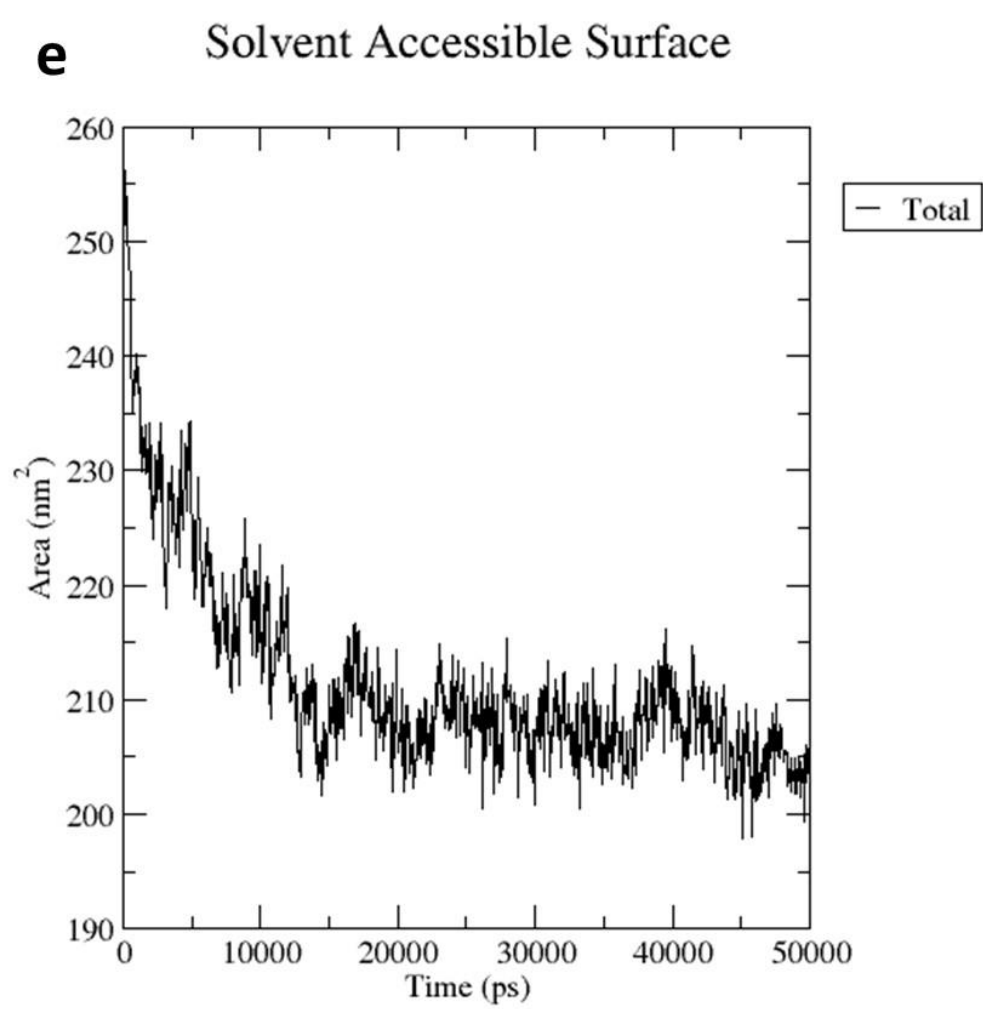

Supplement: Supplementary 3 — Analysis of molecular dynamics simulation for the structures obtained from Robetta (i) NP_249450.1 and (ii) NP_251676.1. (a) The RMSD values; (b) RMSF values; (c) the Radius of gyration (Rg) profile; (d) the hydrogen bond; and (e) SASA descriptors. [file 1787485.f3.pdf]
