# Supplementary material for: Targeting Essential Hypothetical Proteins of Pseudomonas aeruginosa PAO1 for Mining of Novel Therapeutics: An In Silico Approach
Source: Biomed Res Int. 2023 Apr 11;2023:1787485. doi: 10.1155/2023/1787485 (PMC10119676; doi:10.1155/2023/1787485)
Supplement: Supplementary 4 — Table S1: subcellular localization and transmembrane topology. Table S2: list of functions of the proteins found in PPI network. Table S3: properties of secondary structure from SOPMA database. [file 1787485.f4.pdf]

Supplementary Table 1: Subcellular localization and transmembrane topology

Serial No.	RefSeq.	Cellular localization		Transmembrane helices					Signal peptide		
		Psotb	Cello	TMHMM	Phobius	HMMTOP	CCTOP	PROTTER	(SignalP 4.1)	PROTTER	PrediSi
1	NP_252456.1	Cytoplasmic		0	0	0	1	0	No	No	No
2	NP_252782.1	Cytoplasmic		1	1	1	1	1	No	No	No
3	NP_253095.1	Unknown	Cytoplasmic	0	0	1	1	0	No	No	No
4	NP_253252.1	Cytoplasmic Membrane	Inner membrane	13	13	13	14	13	No	Yes	No
5	NP_253326.1	Unknown	Cytoplasmic	0	0	0	1	0	No	No	No
6	NP_253368.1	Unknown	Periplasmic	0	0	0	4	0	No	No	No
7	NP_249450.1	Unknown	Cytoplasmic	0	0	0	1	0	No	No	No
8	NP_250659.1	Unknown	Periplasmic,	0	0	0	1	0	Yes	Yes	Yes
9	NP_250846.1	Cytoplasmic		0	0	0	3	0	No	No	No

10	NP_251676.1	Cytoplasmic Membrane	Inner Membrane	4	5	4	4	5	No	No	Yes
11	NP_252171.1	Cytoplasmic Membrane	Cytoplasmic	0	0	0	1	0	No	No	No
12	NP_252375.1	Cytoplasmic Membrane	Cytoplasmic	0	0	0	0	0	No	No	No
13	NP_253374.1	Cytoplasmic		0	0	0	2	0	No	No	No
14	NP_253434.1	Cytoplasmic		0	0	0	0	0	No	Yes	No
15	NP_253455.1	Unknown	Cytoplasmic	0	0	0	1	0	No	No	No
16	NP_253678.1	Cytoplasmic		0	0	1	1	0	No	No	No
17	NP_253679.1	Cytoplasmic		0	0	0	1	0	No	No	No
18	NP_253685.1	Cytoplasmic		0	0	0	0	0	No	No	No

Supplementary Table 2: List of functions of the proteins found in PPI network

Protein	***Function***
**PA2986 (NP_251676.1)**	

ponA	Penicillin-binding protein 1A
PA1048	Hypothetical protein
ftsA	Cell division protein FtsA
lpxA	Acyl-[acyl-carrier-protein]--UDP-N-acetylglucosamine O-acyltransferase
ispB	Octaprenyl-diphosphate synthase
rpoZ	DNA-directed RNA polymerase subunit omega
lpxD	UDP-3-O-acylglucosamine N-acyltransferase
PA4668	Molecular chaperone LolB
lysS- lysine	tRNA ligase
PA3817	Methyltransferase
PA2983	Translocation protein TolQ
tolR	Translocation protein TolR
PA1041	Hypothetical protein
tolB	Translocation protein TolB
PA4461	ABC transporter ATP-binding protein
secA	Preprotein translocase subunit SecA
yfiB	Hypothetical protein
oprL	Peptidoglycan associated lipoprotein OprL
PA0073	ABC transporter ATP-binding protein
pros	Proline--tRNA ligase
lolA	Outer-membrane lipoprotein carrier protein
secB	Preprotein translocase subunit SecB
PA4594	ABC transporter ATP-binding protein

PA4064	ABC transporter ATP-binding protein
rpsA	30S ribosomal protein S1
PA2989	Hypothetical protein
PA3526	Hypothetical protein
Int	Apolipoprotein N-acyltransferase
rpoB	DNA-directed RNA polymerase subunit beta
tolQ	Translocation protein TolQ
PA2987	Lipoprotein-releasing system ABC transporter ATP-binding protein
pvdT	Pyoverdine biosynthesis protein PvdT
PA0833	Hypothetical protein
ftsJ	Cell division protein FtsJ
exbB1	Transporter ExbB
secD	Preprotein translocase subunit SecD
PA2988	Hypothetical protein
PA4454	Hypothetical protein
lpxK	Tetraacyldisaccharide 4'-kinase
*opr86	*Outer membrane protein Opr86
*PA5568	*Inner membrane protein translocase subunit YidC
*mraY	*Phospho-N-acetylmuramoyl-pentapeptide-transferase
*ComL	*Competence protein ComL
*rpoH	*RNA polymerase sigma factor RpoH
**ostA	**Organic solvent tolerance protein OstA

PA0759 (NP_249450.1)	
soxA	Sarcosine oxidase subunit alpha
gcvT1	Glycine cleavage system aminomethyltransferase T
PA0760	Hypothetical protein
tsf	Elongation factor Ts
rluC	Ribosomal large subunit pseudouridine synthase C
PA3828	Hypothetical protein
PA2736	Hypothetical protein
ftsQ	Cell division protein FtsQ
PA0758	Hypothetical protein
recO	DNA repair protein RecO
PA3142	Hypothetical protein
pyoS5	Pyocin S5
omlA	Outer membrane lipoprotein OmlA
PA2100	Transcriptional regulator
PA0641	Bacteriophage protein
PA1814	Hypothetical protein
iscA	Iron-binding protein IscA
PA1378	Hypothetical protein
lysA	Diaminopimelate decarboxylase
PA2106	Hypothetical protein
sspB	ClpXP protease specificity-enhancing factor
pyrF	Orotidine 5'-phosphate decarboxylase

PA3500	Hypothetical protein
PA2730	Hypothetical protein
PA3801	Hypothetical protein
PA0203	ABC transporter
gcvT2-	Glycine cleavage system protein T2
PA3513- HP	Hypothetical protein
surA-	Chaperone SurA
PA0206-	Spermidine/putrescine ABC transporter ATP-binding protein
prfB-	Peptide chain release factor 1
PA0202-	Amidase
PA2218- HP	Hypothetical protein
PA0665-	Iron-sulfur cluster insertion protein ErpA
PA0715- HP	Hypothetical protein
PA4426- HP	Hypothetical protein
PA3827- HP	Hypothetical protein
*rne	*ribonuclease E
*PA2228- HP	Hypothetical protein
**PA1847	**Fe/S biogenesis protein NfuA
**PA4562 (NP_253252.1)**	
flgC	flagellar basal body rod protein FlgC
groEL	molecular chaperone GroEL
pcnB	poly(A) polymerase
trpS	tryptophan--tRNA ligase

lspA	lipoprotein signal peptidase
murE	UDP-N-acetylmuramoylalanyl-D-glutamate--2,6-diaminopimelate ligase
mutS	DNA mismatch repair protein MutS
arnA	bifunctional UDP-glucuronic acid decarboxylase/UDP-4-amino-4-deoxy-L-arabinose formyltransferase
rpsT	30S ribosomal protein S20<br>rodA- rod shape-determining protein
bioF	8-amino-7-oxononanoate synthase
ftsW	cell division protein FtsW
PA4558	FkbP-type peptidyl-prolyl cis-trans isomerase
dksA	suppressor protein DksA
PA5486	HP
bioB	biotin synthase
murA	UDP-N-acetylglucosamine 1-carboxyvinyltransferase
ftsI	penicillin-binding protein 3
pilQ	type 4 fimbrial biogenesis outer membrane protein PilQ
PA4420	S-adenosyl-methyltransferase MraW
lytB	4-hydroxy-3-methylbut-2-enyl diphosphate reductase
nuoF	NADH dehydrogenase I subunit F
ileS	isoleucine--tRNA ligase
nuoE	NADH-quinone oxidoreductase subunit E
cca	multifunctional tRNA nucleotidyl transferase/2'3'-cyclic phosphodiesterase/2'nucleotidase/phosphatase
glnD	bifunctional uridylyltransferase/uridylyl-removing protein

cysS	cysteine--tRNA ligase
murG	undecaprenyldiphospho-muramoylpentapeptide beta-N-acetylglucosaminyltransferase
dnaE	DNA polymerase III subunit alpha
dnaN	DNA polymerase III subunit beta
dksA	suppressor protein DksA
*nusA	*transcription elongation factor NusA
**PA3685 (NP_252375.1)**	
PA1045	ATP-dependent DNA helicase DinG
PA3683	HP
thiL	thiamine monophosphate kinase
mrcB	penicillin-binding protein 1B
PA4534	HP
gcp	tRNA N6-adenosine threonylcarbamoyltransferase
adk	adenylate kinase
PA1792	UDP-2,3-diacylglucosamine hydrolase
PA3649	zinc metalloprotease
PA3460	acetyltransferase
PA4949	HP
rim	ribosome maturation factor RimM
rimI	peptide n-acetyltransferase RimI
PA3680	ribosomal RNA small subunit methyltransferase J
PA4936	23S rRNA (guanosine(2251)-2'-O)-methyltransferase RlmB
PA3682	HP

PA3681	HP
PA4948	HP
PA0017	Ribosomal RNA small subunit methyltransferase B
Alr	biosynthetic alanine racemase
lpxC	UDP-3-O-[3-hydroxymyristoyl] N-acetylglucosamine deacetylase
PA0022	threonylcarbamoyl-AMP synthase
PA3684	HP
PA1866	HP
dadX	alanine racemase
PA4422	HP
PA4746	HP
PA5335	HP
PA1943	HP
*PA3638	*tRNA(Ile)-lysidine synthase
**PA3767 (NP_252456.1)**	
PA0370	HP
PA1110	HP
PA3981	HP
ribD	riboflavin-specific deaminase/reductase
PA3004	S-methyl-5'-thioinosine phosphorylase
dut	deoxyuridine 5'-triphosphate nucleotidohydrolase
PA1729	HP

PA4627	16S rRNA methyltransferase
PA2631	acetyltransferase
PA3179	ribosomal large subunit pseudouridine synthase B
groES	co-chaperonin GroES
PA1052	HP
upp	uracil phosphoribosyltransferase
PA3968	pseudouridine synthase
Nuh	nonspecific ribonucleoside hydrolase
PA0531	glutamine amidotransferase
ndk	nucleoside diphosphate kinase
PA1742	amidotransferase
cafA	cytoplasmic axial filament protein
pyrR	bifunctional pyrimidine regulatory protein PyrR/uracil phosphoribosyltransferase
PA0733	16S rRNA pseudouridine(516) synthase
PA1885	HP
PA3768	metallo-oxidoreductase
PA3766	aromatic amino acid transporter
*guaA	*GMP synthase
*guaB	*inosine 5'-monophosphate dehydrogenase
**PA4992 (NP_253679.1)**	
PA4990	SMR multidrug efflux transporter

waaA	3-deoxy-D-manno-octulosonic acid transferase
PA3003	HP
PA1478	heme exporter protein CcmD
PA2169	HP
PA5515	HP
PA4167	2,5-diketo-D-gluconate reductase B
hemD	uroporphyrinogen-III synthase
PA4697	HP
PA1648	oxidoreductase
PA1440	HP
PA4485	HP
PA4991	HP
PA4993	HP
PA3008	cell division inhibitor SulA
PA4373	HP
PA0948	HP
PA4828	HP
PA1645	HP
PA4989	transcriptional regulator
PA4372	HP
*PA4441	HP
*PA4098	*short-chain dehydrogenase
**PA3481 (NP_252171.1)**	

hflK	protease subunit HflK
polB	DNA polymerase II
aruH	arginine:pyruvate transaminase AruH
PA4513	oxidoreductase
Bcp	bacterioferritin comigratory protein
PA3980	(dimethylallyl)adenosine tRNA methylthiotransferase
metG	methionine--tRNA ligase
pslD	biofilm formation protein PslD
PA3483	HP
ptpA	phosphotyrosine protein phosphatase
PA2993	HP
mgtE	Mg transporter MgtE
PA5055	HP
pslA	biofilm formation protein PslA
arsC	low molecular weight phosphatase
wbpM	nucleotide sugar epimerase/dehydratase WbpM
hflC	protease subunit HflC
PA3484	HP
PA3485	HP
wzx	O-antigen translocase
PA3480	deoxycytidine triphosphate deaminase

PA4093 (NP_252782.1)	
PA4148	short-chain dehydrogenase
acpP	acyl carrier protein
fabG	3-oxoacyl-[acyl-carrier-protein] reductase FabG
fabH2	3-oxoacyl-ACP synthase III
polA	DNA polymerase I
pqsC	HP
PA3334	acyl carrier protein
PA0214	acyl transferase
PA2705	HP
PA3286	3-oxoacyl-ACP synthase
pchA	salicylate biosynthesis isochorismate synthase
plsX	phosphate acyltransferase
fabD	malonyl CoA-ACP transacylase
pqsB	HP
rhlG	3-oxoacyl-ACP reductase
pchE	dihydroaeruginoic acid synthetase
PA1869	acyl carrier protein
PA4094	transcriptional regulator
pqsD	3-oxoacyl-ACP synthase
**PA4636 (NP_253326.1)**	

dapD	2,3,4,5-tetrahydropyridine-2,6-dicarboxylate N-succinyltransferase
PA0547	transcriptional regulator
PA5468	citrate transporter
PA5271	HP
lptA	lysophosphatidic acid acyltransferase
PA2992	HP
PA3191	two-component sensor
PA5469	HP
algQ	anti-RNA polymerase sigma 70 factor
PA5466	HP
PA5211	HP
pdxB	erythronate-4-phosphate dehydrogenase
PA5567	tRNA modification GTPase TrmE
PA0961	cold-shock protein
PA2797	HP
PA2798	two-component response regulator

No sign is for Protein connected by a single node; The sign (*) is for Protein connected by a single node and the sign (**) for Protein connected between three nodes

Supplementary Table 3: Properties of secondary structure from SOPMA database.

**Parameters**	**NP_249450.1**	**NP_251676.1**
Alpha helix (Hh)	123 is 39.17%	206 is 47.58%
3_{10} helix (Gg)	0 is 0.00%	0 is 0.00%
P_i helix (Ii)	0 is 0.00%	0 is 0.00%
Beta bridge (Bb)	0 is 0.00%	0 is 0.00%
Extended strand (Ee)	60 is 19.11%	83 is 19.17%
Beta turn (Tt)	18 is 5.73%	23 is 5.31%
Bend region (Ss)	0 is 0.00%	0 is 0.00%
Random coil (Cc)	113 is 35.99%	121 is 27.94%
Ambiguous states (?)	0 is 0.00%	0 is 0.00%
Other states	0 is 0.00%	0 is 0.00%
