# Supplementary material for: Targeting Essential Hypothetical Proteins of Pseudomonas aeruginosa PAO1 for Mining of Novel Therapeutics: An In Silico Approach
Source: Biomed Res Int. 2023 Apr 11;2023:1787485. doi: 10.1155/2023/1787485 (PMC10119676; doi:10.1155/2023/1787485)
Supplement: Supplementary 5 — ROC analysis. [file 1787485.f5.zip › Supplementary File 1 (1).pdf]

Accuracy, sensitivity, specificity and ROC area results of 100 proteins functionally known from *Pseudomonas aeruginosa* using the same pipeline used for the HP prediction.

**Software**	**Accuracy (%)**	**Sensitivity (%)**	**Specificity (%)**	**ROC area**
CATH 4.2	100	100	100	1
CDART	100	100	100	1
CDD	100	100	100	1
GO FEAT	100	100	100	1
HHpred	97	100	72.7	0.886
Interpro v 84.0	95	93.7	100	0.968
PANNZER	94	93.3	100	0.967
Pfam v 33.1	91	88.8	100	0.944
PFP-FunD SeqE (PFP)	93	91.4	100	0.957
PFP-FunD SeqE (ESG)	99	98.8	100	0.994
SMART	100	100	100	1
SUPER FAMILY 1.75	100	100	100	1
**Average**	**97.41666667**	**97.16666667**	**97.725**	**0.976333333**
