# Supplementary material for: Targeting Essential Hypothetical Proteins of Pseudomonas aeruginosa PAO1 for Mining of Novel Therapeutics: An In Silico Approach
Source: Biomed Res Int. 2023 Apr 11;2023:1787485. doi: 10.1155/2023/1787485 (PMC10119676; doi:10.1155/2023/1787485)
Supplement: Supplementary 5 — ROC analysis. [file 1787485.f5.zip › Supplementary File 2.pdf]

List of annotated function of 100 proteins with known function from *Pseudomonas aeruginosa* using CATH 4.2, CDART, CDD, GO FEAT, HHpred, Interpro v 84.0, PANNZER, Pfam v 33.1, PFP-FunD SeqE (PFP, ESG) , SMART, SUPER FAMILY 1.75 for ROC analysis.

Serial No.	Entry	Protein name	CATH 4.2	Score	CDART	Score	CDD	Score	GO FEAT	Score	HHpred	
1	Q9I596	Neutral ceramidase	Neutral ceramidase	1(5)	Alkaline ceramidase	1(4)	Neutral/alkaline non-lysosomal ceramidase	1(5)	Neutral ceramidase	1(5)	Neutral ceramidase	1(5)
2	Q9I4D4	NAD(P)H-dependent FMN reductase PA1204	NADPH-dependent FMN reductase	1(5)	Sulfite reductase	0(2)	NADPH-dependent FMN reductase	1(5)	NAD(P)H-dependent FMN reduc	1(5)	NADPH-dependent FMN reductase	1(5)
3	G3XCK0	dTDP-4-dehydrorhamnose reductase	DTDP-4-dehydrorhamnose reductase	1(5)	dTDP-L-rhamnose synthase	0(2)	dTDP-4-dehydrorhamnose reductase	1(5)	dTDP-4-dehydrorhamnose reduct	1(5)	dTDP-4-dehydrorhamnose Reductase	1(5)
4	G3XCK4	Glucose-1-phosphate thymidylyltransferase	Glucose-1-phosphate thymidylyltransferase	1(5)	Glycosyl transferase family protein	1(4)	Glucose-1-phosphate thymidylyltransferase	1(5)	Glucose-1-phosphate thymidylyltı	1(5)	GLUCOSE-1-PHOSPHATE THYMIDYLYLTRAN	1(5)
5	P12019	cAMP/cGMP-dependent 3',5'-cAMP/cGMP phosphodiesterase A	3',5'-cyclic-nucleotide phosphodiesterase	1(5)	cAMP/cGMP-dependent 3',5'-cAMP/cGMI	1(5)	cAMP phosphodiesterases class-II	1(4)	cAMP/cGMP-dependent 3',5'-cA	1(5)	3',5'-cyclic-nucleotide phosphodiesterase 1	1(5)
6	G3XCM4	dTDP-glucose 4,6-dehydratase	dTDP-glucose 4,6-dehydratase	1(5)	dTDP-glucose 4,6-dehydratase	1(5)	dTDP-D-glucose 4,6-dehydratase	1(5)	dTDP-glucose 4,6-dehydratase	1(5)	dTDP-glucose 4,6-dehydratase	1(5)
7	Q9HUU7	L-methionine sulfoximine/L-methionine sulfone acetyltransferase	Phosphinothricin acetyltransferase	1(4)	L-methionine sulfoximine/L-methionine sul	1(5)	L-amino acid N-acyltransferase YncA	0(2)	L-methionine sulfoximine/L-methi	1(5)	Acetyltransferase	1(4)
8	Q9HTW3	Cell division protein ZapA	Cell division protein ZapA	1(5)	Cell division protein ZapA	1(5)	Cell division protein ZapA	1(5)	Cell division protein ZapA	1(5)	Cell division protein ZapA	1(5)
9	Q9HT87	Histidine kinase	PAS domain-containing sensor histidine kinase	1(5)	Histidine kinase	1(5)	Cell wall metabolism sensor histidine kinase '	1(4)	Histidine kinase	1(5)	Putative histidine kinase CovS	1(5)
10	Q55GS4	Probable cyclin-dependent kinase 10	Cyclin-dependent kinase 10	1(5)	Cyclin-dependent kinase 10	1(5)	Cyclin-dependent kinase 10	1(5)	Probable cyclin-dependent kinase	1(5)	Cyclin-Dependent protein Kinase 10	1(5)
11	A0A455VXI1	DNA-directed DNA polymerase	DNA-directed DNA polymerase	1(5)	DNA-directed DNA polymerase	1(5)	DNA-directed DNA polymerase	1(5)	DNA-directed DNA polymerase	1(5)	DNA-directed DNA polymerase	1(5)
12	A0A455W6T9	Thymidylate synthase thyX	Flavin-dependent thymidylate synthase	1(5)	Thymidylate synthase thyX	1(5)	Thymidylate synthase thyX	1(5)	Thymidylate synthase thyX	1(5)	Thymidylate synthase thyX	1(5)
13	A0A455W546	DNA ligase	Nucleic acid-binding proteins	1(3)	DNA ligase	1(5)	DNA ligase	1(5)	DNA ligase	1(5)	DNA ligase	1(5)
14	P95434	Type III needle protein PscF	Type III secretion apparatus needle protein YscF	1(4)	Type III needle protein PscF	1(5)	Type III needle protein PscF	1(5)	Type III needle protein PscF	1(5)	Putative type III secretion protein YscF	1(4)
15	Q9I6S0	Probable transcriptional regulator	LysR family transcriptional regulator	1(5)	Probable transcriptional regulator	1(5)	Probable transcriptional regulator	1(5)	Transcriptional regulator	1(5)	Probable transcriptional regulator; LysR,	1(5)
16	Q9I317	Type III export protein PscE	Type III export protein PscE	1(5)	Type III export protein PscE	1(5)	Type III export protein PscE	1(5)	Type III export protein PscE	1(5)	Type III export protein PscE	1(5)
17	A0A455W465	Lytic enzyme	Lysin A	1(3)	Lytic enzyme	1(5)	Lytic enzyme	1(5)	Lytic enzyme	1(5)	ENLYS_BPMD2 Endolysin A	1(3)
18	A0A455VY99	Phage minor capsid protein	No match	0(2)	Phage minor capsid protein	1(5)	Phage minor capsid protein	1(5)	Minor capsid protein	1(5)	GP7_BPSPP Minor head protein GP7 from phage	1(4)
19	P95435	Type III export protein PscG	Type III export protein PscG	1(5)	Type III export protein PscG	1(5)	Type III export protein PscG	1(5)	Type III export protein PscG	1(5)	TYPE III EXPORT PROTEIN PSCG	1(5)
20	P14756	Elastase	Elastase	1(5)	Elastase	1(5)	Elastase	1(5)	Elastase	1(5)	Elastase	1(5)
21	Q51487	Outer membrane protein OprM	Multidrug efflux outer membrane protein OprN	1(4)	Outer membrane protein OprM	1(5)	Outer membrane protein OprM	1(5)	Outer membrane protein OprM	1(5)	Outer membrane protein oprM	1(5)
22	P55222	cAMP-activated global transcriptional regulator Vfr	cAMP-activated global transcriptional regulator	1(5)	cAMP-activated global transcriptional regu	1(5)	cAMP-activated global transcriptional regulat	1(5)	cAMP-activated global transcript	1(5)	cAMP-activated global transcriptional regulator CI	1(4)
23	P14789	Protease LasA	Protease LasA	1(5)	Protease LasA	1(5)	Protease LasA	1(5)	Protease LasA	1(5)	Protease lasA	1(5)
24	Q9Z4J7	Quinoprotein ethanol dehydrogenase	Quinoprotein alcohol dehydrogenase-like superfa	1(5)	Quinoprotein ethanol dehydrogenase	1(5)	Quinoprotein ethanol dehydrogenase	1(5)	Quinoprotein ethanol dehydrogena	1(5)	QUINOPROTEIN ETHANOL DEHYDROGENAS	1(5)
25	P26276	Phosphomannomutase/phosphoglucomutase	Phosphomannomutase/phosphoglucomutase	1(5)	Phosphomannomutase/phosphoglucomutase	1(5)	Phosphomannomutase/phosphoglucomutase	1(5)	Phosphomannomutase/phosphoglu	1(5)	Phosphomannomutase	1(5)
26	P08308	Ornithine carbamoyltransferase, catabolic	Ornithine carbamoyltransferase	1(5)	Ornithine carbamoyltransferase, catabolic	1(5)	Ornithine carbamoyltransferase, catabolic	1(5)	Ornithine carbamoyltransferase, c	1(5)	ORNITHINE CARBAMOYLTRANSFERASE	1(5)
27	Q9HZ76	UDP-2-acetamido-2-deoxy-3-oxo-D-glucuronate aminotransferase	Aminotransferase, DegT/DnrJ/EryC1/StrS family	1(4)	UDP-2-acetamido-2-deoxy-3-oxo-D-glucu	1(5)	UDP-2-acetamido-2-deoxy-3-oxo-D-glucuron	1(5)	UDP-2-acetamido-2-deoxy-3-oxo	1(5)	Putative UDP-4-amino-4-deoxy-l-arabinose--oxogl	1(3)
28	P22610	Prepilin leader peptidase/N-methyltransferase	Prepilin peptidase	1(4)	Prepilin leader peptidase/N-methyltransfer	1(5)	Prepilin leader peptidase/N-methyltransferase	1(5)	Prepilin leader peptidase/N-methy	1(5)	Peptidase A24B	1(4)
29	G3XCY4	Transcription factor AmrZ	Arc family DNA-binding protein	1(3)	Transcription factor AmrZ	1(5)	Transcription factor AmrZ	1(5)	Transcription factor AmrZ	1(5)	Transcription Factor, Alginate and motility regulato	1(5)
30	P04739	Type IV major pilin protein PilA	Type IV pilin	1(4)	Type IV major pilin protein PilA	1(5)	Type IV major pilin protein PilA	1(5)	Type IV major pilin protein PilA	1(5)	Type IV pilin structural subunit; PilA	1(5)
31	P26876	Triacylglycerol lipase	Lactonizing lipase	1(3)	Triacylglycerol lipase	1(5)	Triacylglycerol lipase	1(5)	Triacylglycerol lipase	1(5)	TRIACYLGLYCEROL LIPASE	1(5)
32	Q51548	L-ornithine N(5)-monooxygenase	L-ornithine N(5)-monooxygenase	1(5)	L-ornithine N(5)-monooxygenase	1(5)	L-ornithine N(5)-monooxygenase	1(5)	L-ornithine N(5)-monooxygenase	1(5)	L-ornithine 5-monooxygenase	1(5)
33	Q9HXU1	Probable transcriptional regulator	TetR family transcriptional regulator	1(5)	Probable transcriptional regulator	1(5)	Probable transcriptional regulator	1(5)	TetR family transcriptional regula	1(5)	Transcriptional regulator	1(5)
34	A0A455W7U5	DNA helicase, phage-associated	Helicase, Snf2 family	1(5)	DNA helicase, phage-associated	1(5)	DNA helicase, phage-associated	1(5)	Putative DNA helicase	1(5)	DNA repair protein RAD5, Helicase	1(4)
35	Q9I638	Probable hydrolase	3-oxoadipate enol-lactone hydrolase	1(5)	Probable hydrolase	1(5)	Probable hydrolase	1(5)	3-oxoadipate enol-lactonase	0(2)	Probable hydrolase	1(5)
36	A0A455VXI3	3'-phosphatase, 5'-polynucleotide kinase, phage-associated	No match	0(2)	3\'-phosphatase, 5\'-polynucleotide kinase,	1(5)	3\'-phosphatase, 5\'-polynucleotide kinase, pha	1(5)	3'-phosphatase, 5'-polynucleotide	1(5)	Polynucleotide kinase; Phosphatase	1(3)
37	Q03023	Serralysin	Serralysin	1(5)	Serralysin	1(5)	Serralysin	1(5)	Serralysin	1(5)	Serralysin	1(5)
38	Q9HZE1	Probable transcriptional regulator	Transcriptional regulator, MarR family	1(5)	Probable transcriptional regulator	1(5)	Probable transcriptional regulator	1(5)	MarR family transcriptional regul	1(5)	Probable transcriptional regulator	1(5)
39	B0FP69	Exotoxin A regulatory protein	No hit	0(2)	Exotoxin A regulatory protein	1(5)	Exotoxin A regulatory protein	1(5)	Exotoxin A regulatory protein	1(5)	EAL phosphodiesterase	0(2)
40	Q9I498	Probable enoyl-CoA hydratase/isomerase	Enoyl-CoA hydratase	1(5)	Probable enoyl-CoA hydratase/isomerase	1(5)	Probable enoyl-CoA hydratase/isomerase	1(5)	Crotonase/enoyl-CoA hydratase f	1(5)	Probable enoyl-CoA hydratase/isomerase	1(5)
41	Q51372	Alginate biosynthesis protein AlgX	Alginate biosynthesis protein AlgX	1(5)	Alginate biosynthesis protein AlgX	1(5)	Alginate biosynthesis protein AlgX	1(5)	Alginate biosynthesis protein Alg	1(5)	Alginate biosynthesis protein AlgX	1(5)
42	P26993	HTH-type transcriptional regulator ExsA	HTH-type transcriptional activator RhaS	1(4)	HTH-type transcriptional regulator ExsA	1(5)	HTH-type transcriptional regulator ExsA	1(5)	HTH-type transcriptional regulato	1(5)	HTH-type transcriptional regulator gadX	1(4)
43	P11759	GDP-mannose 6-dehydrogenase	GDP-mannose 6-dehydrogenase	1(5)	GDP-mannose 6-dehydrogenase	1(5)	GDP-mannose 6-dehydrogenase	1(5)	GDP-mannose 6-dehydrogenase	1(5)	GDP-mannose 6-dehydrogenase	1(5)
44	Q9I194	Acyl-homoserine lactone acylase PvdQ	Acyl-homoserine lactone acylase PvdQ	1(5)	Acyl-homoserine lactone acylase PvdQ	1(5)	Acyl-homoserine lactone acylase PvdQ	1(5)	Acyl-homoserine lactone acylase	1(5)	Acyl-homoserine lactone acylase PvdQ	1(5)
45	Q51507	Isochorismate pyruvate lyase	Isochorismate pyruvate lyase	1(5)	Isochorismate pyruvate lyase	1(5)	Isochorismate pyruvate lyase	1(5)	Isochorismate pyruvate lyase	1(5)	Isomerase, chorismate mutase	1(5)
46	Q00515	Type II secretion system protein H	Type II secretion system protein H	1(5)	Type II secretion system protein H	1(5)	Type II secretion system protein H	1(5)	Type II secretion system protein I	1(5)	Type II secretion system protein H	1(5)
47	Q75QK9	Similar to probable bacteriophage protein	No match	0(2)	Similar to probable bacteriophage protein	1(5)	Similar to probable bacteriophage protein	1(5)	Similar to probable bacteriophage	1(5)	Pyocin tube PA0623; bacteriocin, antimicrobial prc	0(2)
48	G3XCV0	Transcriptional regulator FleQ	Transcriptional regulator FleQ	1(5)	Transcriptional regulator FleQ	1(5)	Transcriptional regulator FleQ	1(5)	Transcriptional regulator FleQ	1(5)	Transcriptional regulator (NtrC family)	1(4)
49	Q8GBY9	Putative oxidoreductase	No match	0(2)	Putative oxidoreductase	1(5)	Putative oxidoreductase	1(5)	Putative oxidoreductase	1(5)	Imidazoleglycerol-phosphate dehydratase	0(2)
50	A0A455VXH5	DNA helicase, phage-associated	ATP-dependent DNA helicase	1(4)	DNA helicase, phage-associated	1(5)	DNA helicase, phage-associated	1(5)	ATP-dependent exonuclease V	0(2)	DNA helicase I	1(5)
51	G3XD23	UDP-N-acetyl-2-amino-2-deoxy-D-glucuronate oxidase	UDP-N-acetyl-2-amino-2-deoxy-D-glucuronate c	1(5)	UDP-N-acetyl-2-amino-2-deoxy-D-glucurc	1(5)	UDP-N-acetyl-2-amino-2-deoxy-D-glucuronat	1(5)	UDP-N-acetyl-2-amino-2-deoxy-]	1(5)	UDP-N-acetylglucosamine 3-dehydrogena	1(3)
52	P11439	Exotoxin A	Exotoxin A	1(5)	Exotoxin A	1(5)	Exotoxin A	1(5)	Exotoxin A	1(5)	Exotoxin A	1(5)
53	A0A455W6U8	Phage DNA-binding protein	No match	0(2)	Phage DNA-binding protein	1(5)	Phage DNA-binding protein	1(5)	Phage DNA-binding protein	1(5)	Nucleotide pyrophosphohydrolase	0(2)
54	P20582	Anthraniloyl-CoA anthraniloyltransferase	Anthraniloyl-CoA anthraniloyltransferase	1(5)	Anthraniloyl-CoA anthraniloyltransferase	1(5)	Anthraniloyl-CoA anthraniloyltransferase	1(5)	Anthraniloyl-CoA anthraniloyltra	1(5)	Anthraniloyl-CoA, Transferase;	1(4)
55	Q06198	RNA polymerase sigma-H factor	RNA polymerase sigma factor	1(5)	RNA polymerase sigma-H factor	1(5)	RNA polymerase sigma-H factor	1(5)	RNA polymerase sigma-H factor	1(5)	RNA polymerase sigma-H factor	1(5)
56	P22608	Type IV pilus assembly ATPase PilB	Type IV pilus assembly ATPase PilB	1(5)	Type IV pilus assembly ATPase PilB	1(5)	Type IV pilus assembly ATPase PilB	1(5)	Type IV pilus assembly ATPase F	1(5)	Type IV-A pilus assembly ATPase PilB	1(5)
57	Q9HXE3	FAD-dependent catabolic D-arginine dehydrogenase DauA	FAD-dependent catabolic D-arginine dehydroger	1(5)	FAD-dependent catabolic D-arginine dehyc	1(5)	FAD-dependent catabolic D-arginine dehydro	1(5)	FAD-dependent catabolic D-argii	1(5)	FAD dependent oxidoreductase	1(3)
58	Q51371	Mannuronan C5-epimerase	Poly (Beta-D-mannuronate) C5 epimerase	1(5)	Mannuronan C5-epimerase	1(5)	Mannuronan C5-epimerase	1(5)	Mannuronan C5-epimerase	1(5)	Poly(beta-D-mannuronate) C5 epimerase	1(4)
59	O33407	Esterase EstA	Esterase EstA	1(5)	Esterase EstA	1(5)	Esterase EstA	1(5)	Esterase EstA	1(5)	Esterase estA	1(5)
60	Q9I3T5	Acetylpolyamine amidohydrolase 1	Acetylpolyamine amidohydrolase 2	1(4)	Acetylpolyamine amidohydrolase 1	1(5)	Acetylpolyamine amidohydrolase 1	1(5)	Acetylpolyamine amidohydrolase	1(5)	Acetylpolyamine aminohydrolase	1(5)
61	O69078	Translational regulator CsrA	Translational regulator CsrA	1(5)	Translational regulator CsrA	1(5)	Translational regulator CsrA	1(5)	Translational regulator CsrA	1(5)	Carbon storage regulator homolog; Csra-like fold	1(5)
62	Q9I596	Neutral ceramidase	Neutral ceramidase	1(5)	Neutral ceramidase	1(5)	Neutral ceramidase	1(5)	Neutral ceramidase	1(5)	Neutral ceramidase	1(5)
63	Q59636	Nucleoside diphosphate kinase	Nucleoside diphosphate kinase	1(5)	Nucleoside diphosphate kinase	1(5)	Nucleoside diphosphate kinase	1(5)	Nucleoside diphosphate kinase	1(5)	Nucleoside diphosphate kinase	1(5)
64	Q9HV27	Cyclic di-GMP phosphodiesterase PA4781	Cyclic di-GMP phosphodiesterase PA4781	1(5)	Cyclic di-GMP phosphodiesterase PA4781	1(5)	Cyclic di-GMP phosphodiesterase PA4781	1(5)	Cyclic di-GMP phosphodiesteras	1(5)	Cyclic di-GMP phosphodiesterase	1(5)
65	Q9I2A0	3-hydroxy-3-isohexenylglutaryl-CoA/hydroxy-methylglutaryl-CoA lyase	Hydroxymethylglutaryl-CoA lyase	1(5)	3-hydroxy-3-isohexenylglutaryl-CoA/hydrc	1(5)	3-hydroxy-3-isohexenylglutaryl-CoA/hydroxy-	1(5)	3-hydroxy-3-isohexenylglutaryl-Cc	1(5)	Hydroxymethylglutaryl-CoA lyase	1(5)
66	Q03456	Ferric uptake regulation protein	Ferric uptake regulation protein	1(5)	Ferric uptake regulation protein	1(5)	Ferric uptake regulation protein	1(5)	Ferric uptake regulation protein	1(5)	Ferric uptake regulation protein	1(5)
67	Q9HZQ8	Aminopeptidase	Aminopeptidase	1(5)	Aminopeptidase	1(5)	Aminopeptidase	1(5)	Aminopeptidase	1(5)	Aminopeptidase	1(5)
68	P20586	p-hydroxybenzoate hydroxylase	p-hydroxybenzoate hydroxylase	1(5)	p-hydroxybenzoate hydroxylase	1(5)	p-hydroxybenzoate hydroxylase	1(5)	p-hydroxybenzoate hydroxylase	1(5)	4-hydroxybenzoate 3-monooxygenase	1(3)
69	Q9ZN70	Exopolyphosphatase	Exopolyphosphatase	1(5)	Exopolyphosphatase	1(5)	Exopolyphosphatase	1(5)	Exopolyphosphatase	1(5)	Exopolyphosphatase	1(5)
70	Q9HTJ1	NAD/NADP-dependent betaine aldehyde dehydrogenase	NAD/NADP-dependent betaine aldehyde dehydr	1(5)	NAD/NADP-dependent betaine aldehyde d	1(5)	NAD/NADP-dependent betaine aldehyde dehy	1(5)	NAD/NADP-dependent betaine al	1(5)	NAD/NADP-dependent betaine aldehyde dehydrog	1(5)
71	Q9HXM1	Histone deacetylase-like amidohydrolase	Histone deacetylase-like amidohydrolase	1(5)	Histone deacetylase-like amidohydrolase	1(5)	Histone deacetylase-like amidohydrolase	1(5)	Histone deacetylase-like amidohy	1(5)	HISTONE DEACETYLASE-LIKE AMIDOHYDR(	1(5)
72	P51691	Arylsulfatase	Arylsulfatase	1(5)	Arylsulfatase	1(5)	Arylsulfatase	1(5)	Arylsulfatase	1(5)	Arylsulfatase	1(5)
73	Q9HVD1	Lipid A deacylase PagL	Lipid A deacylase	1(5)	Lipid A deacylase PagL	1(5)	Lipid A deacylase PagL	1(5)	Lipid A deacylase PagL	1(5)	Outer membrane porin F	0(2)
74	Q9I2Y2	Phosphoserine phosphatase ThrH	Phosphoserine phosphatase ThrH	1(5)	Phosphoserine phosphatase ThrH	1(5)	Phosphoserine phosphatase ThrH	1(5)	Phosphoserine phosphatase ThrH	1(5)	Phosphoserine phosphatase	1(4)
75	Q9I5F3	FMN-dependent NAD(P)H:quinone oxidoreductase 1	FMN-dependent NADH-azoreductase 1	1(4)	FMN-dependent NAD(P)H:quinone oxidor	1(5)	FMN-dependent NAD(P)H:quinone oxidoredu	1(5)	FMN-dependent NAD(P)H:quino	1(5)	FMN-dependent NADH-azoreductase 1	1(4)
76	P24559	Type IV pilus retractation ATPase PilT	Type IV pili twitching motility protein PilT	1(5)	Type IV pilus retractation ATPase PilT	1(5)	Type IV pilus retractation ATPase PilT	1(5)	Type IV pilus retractation ATPas	1(5)	Pilus retraction ATPase PilT	1(5)
77	S0HPF7	Type IV pilus biogenesis factor PilY1	No match	0(2)	Type IV pilus biogenesis factor PilY1	1(5)	Type IV pilus biogenesis factor PilY1	1(5)	Type IV pilus biogenesis factor P	1(5)	Type 4 fimbrial biogenesis protein PilY1	1(5)
78	Q9I1X7	Multifunctional non-homologous end joining protein LigD	Multifunctional non-homologous end joining prot	1(5)	Multifunctional non-homologous end joinin	1(5)	Multifunctional non-homologous end joining p	1(5)	Multifunctional non-homologous (	1(5)	Non-homologous end joining Polymerase	1(5)
79	Q9I700	Beta-alanine--pyruvate aminotransferase	Type I PLP-dependent aspartate aminotransferase	1(3)	Beta-alanine--pyruvate aminotransferase	1(5)	Beta-alanine--pyruvate aminotransferase	1(5)	Beta-alanine--pyruvate aminotran	1(5)	Beta-alanine-pyruvate aminotransferase	1(5)
80	Q9I5I6	NAD-dependent L-serine dehydrogenase	NAD(P)-binding Rossmann-like Domain	1(3)	NAD-dependent L-serine dehydrogenase	1(5)	NAD-dependent L-serine dehydrogenase	1(5)	NAD-dependent L-serine dehydrc	1(5)	NAD-dependent glycerol-3-phosphate dehydrogena	1(4)
81	Q9HUF7	Lipopolysaccharide core heptose(I) kinase RfaP	No match	0(2)	Lipopolysaccharide core heptose(I) kinase	1(5)	Lipopolysaccharide core heptose(I) kinase Rf	1(5)	Lipopolysaccharide core heptose	1(5)	Lipopolysaccharide core heptose(I) kinase RfaP	1(5)
82	Q9HZP8	Enoyl-[acyl-carrier-protein] reductase [NADH]	Enoyl-[acyl-carrier-protein] reductase [NADH]	1(5)	Enoyl-[acyl-carrier-protein] reductase [NA	1(5)	Enoyl-[acyl-carrier-protein] reductase [NAD	1(5)	Enoyl-[acyl-carrier-protein] redu	1(5)	Enoyl-ACP Reductase	1(5)
83	Q02MM2	CRISPR-associated endonuclease Cas6/Csy4	CRISPR-associated endoribonuclease Cas6/Csy4	1(5)	CRISPR-associated endonuclease Cas6/Cs	1(5)	CRISPR-associated endonuclease Cas6/Csy4	1(5)	CRISPR-associated endonuclease	1(5)	type I-F CRISPR-associated endoribonuclease Cas(	1(5)
84	Q9HWC1	Type III pantothenate kinase	Type III pantothenate kinase	1(5)	Type III pantothenate kinase	1(5)	Type III pantothenate kinase	1(5)	Type III pantothenate kinase	1(5)	Type III pantothenate kinase	1(5)
85	Q9HWK6	Lysyl endopeptidase	Lysyl endopeptidase	1(5)	Lysyl endopeptidase	1(5)	Lysyl endopeptidase	1(5)	Lysyl endopeptidase	1(5)	Lysyl endopeptidase	1(5)
86	Q9I6H0	Acetylpolyamine amidohydrolase 2	Acetylpolyamine amidohydrolase 2	1(5)	Acetylpolyamine amidohydrolase 2	1(5)	Acetylpolyamine amidohydrolase 2	1(5)	Acetylpolyamine amidohydrolase	1(5)	Acetylpolyamine aminohydrolase	1(5)
87	P26275	Positive alginate biosynthesis regulatory protein	DNA-binding response regulator AlgR	1(5)	Positive alginate biosynthesis regulatory pr	1(5)	Positive alginate biosynthesis regulatory prote	1(5)	Positive alginate biosynthesis reg	1(5)	Methyl-accepting/DNA response regulator	0(2)
88	Q9HYF1	Polyphosphate:AMP phosphotransferase	Polyphosphate:AMP phosphotransferase	1(5)	Polyphosphate:AMP phosphotransferase	1(5)	Polyphosphate:AMP phosphotransferase	1(5)	Polyphosphate:AMP phosphotran	1(5)	Polyphosphate:AMP phosphotransferase	1(5)
89	Q9I2N0	Fanconi-associated nuclease 1 homolog	Fanconi-associated nuclease	1(4)	Fanconi-associated nuclease 1 homolog	1(5)	Fanconi-associated nuclease 1 homolog	1(5)	Fanconi-associated nuclease 1 ho	1(5)	Fanconi-associated nuclease 1 homolog	1(5)
90	Q8RNT4	Linoleate 9/13-lipoxygenase	Arachidonate 5-lipoxygenase a	1(4)	Linoleate 9/13-lipoxygenase	1(5)	Linoleate 9/13-lipoxygenase	1(5)	Linoleate 9/13-lipoxygenase	1(5)	Arachidonate 5-lipoxygenase	1(4)
91	P0DPC1	Phenazine biosynthesis protein PhzD2	Phenazine biosynthesis protein PhzD	1(5)	Phenazine biosynthesis protein PhzD2	1(5)	Phenazine biosynthesis protein PhzD2	1(5)	Phenazine biosynthesis protein Pl	1(5)	phenazine biosynthesis protein phzD	1(5)

92	P48636	Cytokinin riboside 5'-monophosphate phosphoribohydrolase	Cytokinin riboside 5'-monophosphate phosphorib	1(5)	Cytokinin riboside 5\'-monophosphate phos	1(5)	Cytokinin riboside 5\'-monophosphate phospho	1(5)	Cytokinin riboside 5'-monophosp	1(5)	Putative cytokinin riboside 5'-monophosphate phosp	1(5)
93	Q59643	Delta-aminolevulinic acid dehydratase	Delta-aminolevulinic acid dehydratase	1(5)	Delta-aminolevulinic acid dehydratase	1(5)	Delta-aminolevulinic acid dehydratase	1(5)	Delta-aminolevulinic acid dehydr	1(5)	Delta-aminolevulinic acid dehydratase	1(5)
94	P24474	Nitrite reductase	Nitrite reductase	1(5)	Nitrite reductase	1(5)	Nitrite reductase	1(5)	Nitrite reductase	1(5)	Nitrite reductase	1(5)
95	P0DTF7	Cyclic AMP-AMP-AMP synthase	No match	0(2)	Cyclic AMP-AMP-AMP synthase	1(5)	Cyclic AMP-AMP-AMP synthase	1(5)	Nucleotidyltransferase	0(2)	Cyclic AMP-GMP synthase	1(5)
96	Q9I6M7	Type VI secretion system spike protein VgrG2b	Type VI secretion protein VgrG	1(5)	Type VI secretion system spike protein Vgr	1(5)	Type VI secretion system spike protein VgrG2	1(5)	Type VI secretion system spike pr	1(5)	VGRG1, VALINE-GLYCINE REPEAT PROTEIN	0(2)
97	Q9HTH8	L-carnitine dehydrogenase	L-carnitine dehydrogenase	1(5)	L-carnitine dehydrogenase	1(5)	L-carnitine dehydrogenase	1(5)	L-carnitine dehydrogenase	1(5)	3-hydroxybutyryl-coA dehydrogenase	1(3)
98	Q9HU21	dTDP-4-dehydrorhamnose 3,5-epimerase	dTDP-4-dehydrorhamnose 3,5-epimerase	1(5)	dTDP-4-dehydrorhamnose 3,5-epimerase	1(5)	dTDP-4-dehydrorhamnose 3,5-epimerase	1(5)	dTDP-4-dehydrorhamnose 3,5-ep	1(5)	DTDP-4-DEHYDRORHAMNOSE 3,5-EPIMERAS	1(5)
99	P14532	Cytochrome c551 peroxidase	Cytochrome c peroxidase	1(5)	Cytochrome c551 peroxidase	1(5)	Cytochrome c551 peroxidase	1(5)	Cytochrome c551 peroxidase	1(5)	Cytochrome c551 peroxidase	1(5)
100	P54292	Regulatory protein RhlR	LuxR family transcriptional regulator	0(2)	Regulatory protein RhlR	1(5)	Regulatory protein RhlR	1(5)	Regulatory protein RhlR	1(5)	LuxR family transcriptional regulator	0(2)

Interpro v 84.0	Score	PANNZER	Score	Pfam v 33.1	Score	PFP-FunD SeqE (PFP)	Score	PFP-FunD SeqE (ESG)	Score	SMAR	Score	SUPER FAMILY 1.75	Score
Neutral/alkaline nonlysosomal ce	1(5)	Flagellar biosynthesis anti-sigma factor FlgM	0(2)	Neutral/alkaline non-lysosomal ceramidas	1(5)	ceramide catabolic process	1(4)	ceramidase activity	1(4)	neutral/alkaline ceramidases	1(5)	Fibronectin type III	0(2)
oxidoreductase activity	1(3)	Chromate reductase	0(2)	NADPH-dependent FMN reductase	1(5)	FMN binding	1(4)	FMN reductase (NADPH) activity	1(5)	FMN-dependent NADPH-azoreductas	1(5)	Flavoproteins	0(2)
dTDP-4-dehydrorhamnose reduct	1(5)	dTDP-4-dehydrorhamnose reductase	1(5)	RmlD substrate binding domain	1(3)	NADP binding	1(2)	dTDP-4-dehydrorhamnose reductase activity	1(5)	dTDP-4-dehydrorhamnose reductase	1(5)	NAD(P)-binding Rossmann-fold domains	0(2)
Glucose-1-phosphate thymidylylt	1(5)	Glucose-1-phosphate thymidylyltransferase	1(5)	Nucleotidyl transferase	1(4)	glucose-1-phosphate adenylyltransferase a	1(4)	glucose-1-phosphate thymidylyltransferase activity	1(5)	Glucose-1-phosphate thymidylyltrans	1(5)	Nucleotide-diphospho-sugar transferases	1(3)
Cyclic-AMP phosphodiesterase, c	1(5)	cAMP/cGMP-dependent 3',5'-cAMP/cGMP phosp	1(5)	cAMP phosphodiesterases class-II	1(5)	cAMP/cGMP-dependent 3',5'-cAMP/cGM	1(5)	3',5'-cyclic-AMP phosphodiesterase activity	1(5)	3',5'-cyclic-AMP phosphodiesterase a	1(5)	Metallo-hydrolase/oxidoreductase	0(2)
dTDP-glucose 4,6-dehydratase	1(5)	dTDP-glucose 4,6-dehydratase	1(5)	GDP-mannose 4,6 dehydratase	1(4)	dTDP-glucose 4,6-dehydratase	1(5)	dTDP-glucose 4,6-dehydratase activity	1(5)	dTDP-glucose 4,6-dehydratase activit	1(5)	NAD(P)-binding Rossmann-fold domains	0(2)
N-acetyltransferase activity	1(4)	Phosphinothricin N-acetyltransferase	1(3)	Acetyltransferase (GNAT) domain	1(4)	acetyltransferase activity	1(3)	N-acetyltransferase activity	1(4)	L-methionine sulfoximine/L-methionin	1(5)	Acyl-CoA N-acyltransferases (Nat)	1(5)
Cell division protein ZapA-like	1(5)	Cell division protein ZapA	1(5)	Cell division protein ZapA	1(5)	ATP binding	0(2)	division septum assembly	1(4)	Cell division protein ZapA	1(5)	Cell division protein ZapA-like	1(5)
phosphorelay sensor kinase activ	1(4)	Alginate biosynthesis sensor protein KinB	1(3)	Sensor domain of alginate biosynthesis sensor pro	1(4)	protein histidine kinase activity	1(5)	phosphorelay sensor kinase activity	1(4)	His Kinase A (phosphoacceptor)	1(5)	ATPase domain of HSP90 chaperone/DNA to	1(5)
protein kinase activity	1(4)	Cell division protein kinase 10	1(2)	Protein kinase domain	1(4)	cyclin-dependent protein kinase activity	1(5)	cyclin-dependent protein serine/threonine kinase activity	1(5)	Probable cyclin-dependent kinase 10	1(5)	Protein kinase-like (PK-like)	1(4)
DNA polymerase III, alpha subuni	1(5)	DNA polymerase III alpha subunit	1(5)	Bacterial DNA polymerase III alpha NTPase domai	1(5)	DNA-directed DNA polymerase activity	1(5)	DNA-directed DNA polymerase activity	1(5)	DNA polymerase III alpha subunit	1(5)	No hit found	0(2)
Thymidylate synthase ThyX	1(5)	Thymidylate synthase thyX	1(5)	Thymidylate synthase complementing protein	1(5)	thymidylate synthase activity	1(4)	thymidylate synthase (FAD) activity	1(5)	thymidylate synthase (FAD) activity	1(5)	Thymidylate synthase-complementing protei	1(5)
DNA ligase (ATP) activity	1(5)	PBCV-1 DNA ligase	1(4)	DNA ligase OB-like domain	1(5)	DNA ligase activity	1(5)	DNA ligase (ATP) activity	1(5)	DNA ligase (ATP) activity	1(5)	DNA ligase/mRNA capping enzyme, catalytic	1(5)
Type III secretion, needle-protein	1(5)	Type III export protein PscF	1(5)	Type III secretion needle MxiH, YscF, SsaG, EprI, Ps	1(5)	type III protein secretion system complex	1(4)	type III protein secretion system complex	1(4)	Type III export protein PscF	1(5)	MxiH-like	0(2)
DNA-binding transcription factor	1(5)	Transcriptional regulator	1(5)	LysR substrate binding domain	1(4)	regulation of transcription, DNA-template	1(5)	DNA-binding transcription factor activity	1(5)	Cys regulon transcriptional activator C	1(4)	Periplasmic binding protein-like II	0(2)
Type III secretion system, secreti	1(5)	Type III export protein PscE	1(5)	Type III secretion system, cytoplasmic E componer	1(5)	protein secretion by the type III secretion syst	1(4)	protein secretion by the type III secretion system	1(4)	Type III export protein PscE	1(5)	No hit found	0(2)
chitinase activity	1(4)	Lytic enzyme	1(5)	Chitinase class I	1(4)	chitinase activity	1(4)	chitin catabolic process	1(4)	chitinase activity	1(3)	Lysozyme-like	1(3)
No hit found	0(2)	Phage minor capsid protein	1(5)	Phage Mu protein F like protein	1(5)	viral capsid	1(4)	viral capsid	1(4)	phage-related proteins	1(4)	No hit found	0(2)
Type III secretion system YscG	1(5)	Type III export protein PscG	1(5)	Bacterial type II secretion system chaperone prote	1(5)	protein secretion by the type III secretion s	1(3)	protein secretion by the type III secretion system	1(3)	Type III export protein PscG	1(5)	No hit found	0(2)
metalloendopeptidase activity	1(4)	Organic solvent tolerant elastase	1(3)	Thermolysin metallopeptidase, alpha-helical doma	1(4)	metalloendopeptidase activity	1(4)	metalloendopeptidase activity	1(4)	Elastase	1(5)	Metalloproteases ("zincins"), catalytic domai	0(2)
Outer membrane efflux protein	1(5)	Major intrinsic multiple antibiotic resistance efflu	0(2)	Outer membrane efflux protein	1(5)	cell outer membrane	1(5)	cell outer membrane	1(5)	Outer membrane protein OprM	1(5)	Outer membrane efflux proteins (OEP)	1(5)
DNA-binding transcription factor	1(4)	CRP/FNR family transcriptional regulator, cyclic A	1(5)	Cyclic nucleotide-binding domain	1(4)	cAMP-dependent protein kinase regulator	1(3)	DNA-binding transcription factor activity	1(3)	Cyclic AMP receptor-like protein	1(4)	cAMP-binding domain-like	1(4)
Peptidase M23A, B-lytic metalloe	1(3)	Protease LasA	1(5)	Peptidase family M23	1(3)	proteolysis	1(4)	proteolysis	1(4)	Protease LasA	1(5)	Duplicated hybrid motif	0(2)
PQQ-dependent dehydrogenase,	1(4)	Quinoprotein ethanol dehydrogenase	1(5)	PQQ enzyme repeat	1(4)	alcohol dehydrogenase (cytochrome c(L))	1(4)	alcohol dehydrogenase (cytochrome c) activity	1(4)	Quinoprotein alcohol dehydrogenase (	1(5)	Quinoprotein alcohol dehydrogenase-like	1(5)
Alpha-D-phosphohexomutase su	1(5)	Phosphoglucomutase	1(5)	Phosphoglucomutase/phosphomannomutase, alpl	1(5)	Phosphomannomutase/phosphoglucomutas	1(5)	Phosphomannomutase/phosphoglucomutase	1(5)	Phosphomannomutase/phosphoglucom	1(5)	Phosphoglucomutase, first 3 domains	1(5)
Aspartate/ornithine carbamoyltra	1(5)	Ornithine carbamoyltransferase	1(5)	Aspartate/ornithine carbamoyltransferase, Asp/Or	1(5)	ornithine carbamoyltransferase activity	1(5)	ornithine carbamoyltransferase activity	1(5)	Ornithine carbamoyltransferase, catab	1(5)	Aspartate/ornithine carbamoyltransferase	1(5)
DegT/DnrJ/EryC1/StrS aminotran	1(4)	UDP-2-acetamido-2-dideoxy-d-ribo-hex-3-uluror	1(5)	DegT/DnrJ/EryC1/StrS aminotransferase family	1(4)	glycine hydroxymethyltransferase activity	1(2)	UDP-4-amino-4-deoxy-L-arabinose aminotransferase	1(4)	UDP-2-acetamido-2-deoxy-3-oxo-D-	1(4)	PLP-dependent transferases	0(2)
Peptidase A24A, prepilin type IV,	1(4)	Type 4 prepilin-like proteins leader peptide-proce	1(5)	Bacterial Peptidase A24 N-terminal domain	1(4)	methyltransferase activity	1(4)	N-methyltransferase activity	1(5)	Type 4 prepilin-like proteins leader p	1(5)	No hit found	0(2)
DNA binding	1(4)	Alginate biosynthesis transcriptional activator Alg	1(2)	Arc-like DNA binding domain	1(40	positive regulation of transcription, DNA-	1(4)	DNA-binding transcription factor activity	1(4)	Arc-like DNA binding domain protei	1(3)	Ribbon-helix-helix	0(2)
Fimbrial protein pilin	1(3)	Type IV pilus assembly protein PilA	1(5)	Pilin (bacterial filament)	1(4)	pilus	1(3)	type IV pilus-dependent motility	1(5)	Fimbrial protein	1(3)	Pili subunits	1(4)
No hit found	0(2)	Triacylglycerol acylhydrolase	1(5)	alpha/beta hydrolase fold	1(4)	triglyceride lipase activity	1(4)	triglyceride lipase activity	1(4)	Triacylglycerol lipase	1(5)	alpha/beta-Hydrolases	0(2)
L-lysine 6-monooxygenase/L-orn	1(2)	L-ornithine 5-monooxygenase, PvdA of pyoverdir	1(5)	L-lysine 6-monooxygenase (NADPH-requiring)	1(20	N,N-dimethylaniline monooxygenase activ	1(3)	N,N-dimethylaniline monooxygenase activity	1(3)	L-ornithine N(5)-monooxygenase	1(5)	FAD/NAD(P)-binding domain	0(2)
DNA binding	1(3)	HTH-type transcriptional regulator betI	1(3)	Bacterial regulatory proteins, tetR family	1(3)	DNA-binding transcription factor activity	1(4)	DNA-binding transcription factor activity	1(4)	TetR family transcriptional regulator	1(5)	Homeodomain-like	0(2)
nucleosome-dependent ATPase a	1(2)	DNA helicase, phage-associated	1(5)	Helicase conserved C-terminal domain	1(4)	helicase activity	1(4)	helicase activity	1(4)	Putative helicase	1(5)	P-loop containing nucleoside triphosphate hy	0(2)
3-oxoadipate enol-lactonase 1/2	0(2)	3-oxoadipate enol-lactonase 1	1(2)	alpha/beta hydrolase fold	1(2)	hydrolase activity	1(5)	carboxylic ester hydrolase activity	1(4)	3-oxoadipate enol-lactonase	1(3)	alpha/beta-Hydrolases	1(5)
No hit found	0(2)	3'-phosphatase, 5'-polynucleotide kinase	1(5)	HAD superfamily, subfamily IIIB (Acid phosphatase	1(2)	kinase binding	1(2)	protein kinase binding	1(2)	No result	0(2)	HAD-like	0(2)
Peptidase M10B	0(2)	Alkaline metalloendoprotease	0(2)	Metallo-peptidase family M12B Reprolysin-like	0(2)	metalloendopeptidase activity	0(2)	metalloendopeptidase activity	0(2)	Serralysin	1(5)	beta-Roll	0(2)
DNA-binding transcription factor	1(3)	Transcriptional regulator	1(5)	MarR family	0(2)	DNA-binding transcription factor activity	1(4)	DNA-binding transcription factor activity	1(4)	Homoprotocatechuate degradation op	1(3)	"Winged helix" DNA-binding domain	1(3)
No hit found	0(2)	Exotoxin	1(5)	EAL domain	0(2)	nucleic acid binding	0(2)	positive regulation of transcription, DNA-templated	0(2)	Exotoxin A regulatory protein	1(5)	EAL domain-like	0(2)
Enoyl-CoA hydratase/isomerase	1(5)	Short-chain-enoyl-CoA hydratase	1(5)	Enoyl-CoA hydratase/isomerase	1(5)	enoyl-CoA hydratase activity	1(4)	enoyl-CoA hydratase activity	1(5)	Enoyl-CoA hydratase/isomerase	1(5)	ClpP/crotonase	0(2)
alginic acid biosynthetic process	1(5)	Alginate biosynthesis protein AlgX	1(5)	SGNH hydrolase-like domain, acetyltransferase Alg	0(2)	alginic acid biosynthetic process	1(5)	alginic acid biosynthetic process	1(5)	Alginate biosynthesis protein AlgX	1(5)	SGNH hydrolase	0(2)
FTranscription regulator HTH, Ara	1(3)	Transcriptional activator protein ExsA/virf	1(5)	Helix-turn-helix domain	0(2)	DNA-binding transcription factor activity	1(4)	DNA-binding transcription factor activity	1(4)	Exoenzyme S synthesis regulatory pro	1(5)	Homeodomain-like	0(2)
UDP-glucose/GDP-mannose dehy	1(5)	GDP-mannose 6-dehydrogenase	1(5)	UDP-glucose/GDP-mannose dehydrogenase family	1(5)	UDP-glucose 6-dehydrogenase activity	1(3)	GDP-mannose 6-dehydrogenase activity	1(5)	GDP-mannose 6-dehydrogenase	1(5)	6-phosphogluconate dehydrogenase C-termi	1(4)
Penicillin/GL-7-ACA/AHL/aculeac	0(2)	Acyl-homoserine lactone acylase subunit beta	1(5)	Penicillin amidase	0(2)	glutaryl-7-aminocephalosporanic-acid acy	1(2)	hydrolase activity, acting on carbon-nitrogen (but not pep	0(2)	Acyl-homoserine lactone acylase Pvd	1(5)	N-terminal nucleophile aminohydrolases (Nt	0(2)
Salicylate biosynthesis protein Pc	0(2)	Salicylate biosynthesis protein pchB	0(2)	Chorismate mutase type II	1(3)	carbon-oxygen lyase activity	0(2)	isochorismate pyruvate lyase activity	1(5)	Isochorismate pyruvate lyase	1(5)	Chorismate mutase II	0(2)
Type II secretion system protein (	1(5)	Type II secretion system protein GspH	1(5)	Type II transport protein GspH	1(5)	protein secretion by the type II secretion s	1(4)	protein secretion by the type II secretion system	1(4)	Type II secretion system protein H	1(5)	Pili subunits	0(2)
Tail tube protein	1(4)	Phage tail tube protein,phage major tail tube pro	1(3)	Phage tail tube protein FII	1(4)	viral genome ejection through host cell en	1(3)	viral genome ejection through host cell envelope, contrac	1(4)	Similar to probable bacteriophage pr	1(5)	No hit found	0(2)
transcription factor binding	1(5)	Sigma-54 specific transcriptional regulator, flagel	1(2)	Sigma-54 interaction domain, Flagellar regulatory	1(2)	transcription factor binding	1(4)	transcription factor binding	1(4)	Nitrogen assimilation regulatory prote	1(3)	P-loop containing nucleoside triphosphate hy	0(2)
No hit found	0(2)	Putative oxidoreductase	1(5)	No hit found	0(2)	imidazoleglycerol-phosphate dehydratase	0(2)	imidazoleglycerol-phosphate dehydratase activity	0(2)	Putative oxidoreductase	1(5)	No hit found	0(2)
No hit found	0(2)	ATP-dependent exonuclease V	1(2)	AAA domain	0(2)	helicase activity	1(4)	5'-3' DNA helicase activity	1(4)	ATP-dependent exonuclease V	0(2)	P-loop containing nucleoside triphosphate hy	0(2)
nucleotide binding	0(2)	Oxidoreductase	0(2)	Oxidoreductase family, NAD-binding Rossmann fol	0(2)	oxidoreductase activity	0(2)	trans-1,2-dihydrobenzene-1,2-diol dehydrogenase activit	0(2)	UDP-N-acetyl-2-amino-2-deoxy-D-gl	1(5)	NAD(P)-binding Rossmann-fold domains	0(2)
NAD+-diphthamide ADP-ribosyltr	0(2)	Exotoxin A	1(5)	Exotoxin A binding	1(5)	serine-type endopeptidase activity	0(2)	toxin activity	1(4)	Exotoxin A	1(5)	Exotoxin A, middle domain	1(5)
No hit found	0(2)	Phage DNA-binding protein	1(5)	MazG-like family	0(2)	DNA binding	1(3)	rRNA (guanine-N7-)-methyltransferase activity	0(2)	No result	0(2)	all-alpha NTP pyrophosphatases	0(2)
3-oxoacyl-[acyl-carrier-protein] s	0(2)	PQB biosynthetic 3-oxoacyl-[acyl-carrier-protein	0(2)	3-Oxoacyl-[acyl-carrier-protein (ACP)] synthase III	0(2)	transferase activity, transferring acyl grou	1(2)	3-oxoacyl-[acyl-carrier-protein] synthase activity	0(2)	Anthraniloyl-CoA anthraniloyltransfe	1(5)	Thiolase-like	0(2)
RNA polymerase sigma-70 like	1(5)	ECF RNA polymerase sigma-E factor	1(3)	Sigma-70, region 4	1(4)	transcription initiation from bacterial-type	1(3)	sigma factor activity	1(4)	RNA polymerase sigma-H factor	1(5)	Sigma2 domain of RNA polymerase sigma fa	1(5)
ATPase, type IV, pilus assembly, P	1(5)	Type IV pilus biogenesis protein PilB	1(5)	Type II/IV secretion system protein	1(4)	ATP binding	0(2)	type IV pilus biogenesis	1(4)	Type 4 fimbrial assembly protein PilI	1(5)	P-loop containing nucleoside triphosphate hy	0(2)
oxidoreductase activity	1(2)	D-arginine dehydrogenase	1(5)	FAD dependent oxidoreductase	1(5)	flavin adenine dinucleotide binding	0(2)	D-amino-acid dehydrogenase activity	1(4)	FAD-dependent catabolic D-arginine	1(5)	FAD/NAD(P)-binding domain	1(3)
No hit found	0(2)	Alginate-c5-mannuronan-epimerase	1(4)	Periplasmic copper-binding protein (NosD)	0(2)	isomerase activity	0(2)	isomerase activity	0(2)	Mannuronan C5-epimerase	1(5)	Pectin lyase-like	0(2)
Lipase, autotransporter EstA	0(2)	Phospholipase/lecithinase/hemolysin	0(2)	Autotransporter beta-domain	0(2)	hydrolase activity, acting on ester bonds	1(3)	hydrolase activity, acting on ester bonds	1(3)	Esterase EstA	1(5)	Autotransporter	0(2)
Histone deacetylase family	0(2)	Acetylpolyamine amidohydrolase	1(5)	Histone deacetylase domain	0(2)	deacetylase activity	1(2)	NAD-dependent histone deacetylase activity (H3-K14 sp	0(2)	Acetylpolyamine amidohydrolase 1	1(5)	Arginase/deacetylase	0(2)
Translational regulator CsrA	1(5)	Translational regulator CsrA	1(5)	Global regulator protein family	1(4)	regulation of translational initiation	1(4)	mRNA 5'-UTR binding	1(3)	Translational regulator CsrA	1(5)	CsrA-like	1(5)
Neutral/alkaline nonlysosomal ce	1(5)	Flagellar biosynthesis anti-sigma factor FlgM	0(2)	Neutral/alkaline non-lysosomal ceramidase, N-ter	1(5)	ceramide catabolic process	1(4)	ceramidase activity	1(4)	Neutral ceramidase	1(5)	No hit found	0(2)
Nucleoside diphosphate kinase	1(5)	Nucleoside diphosphate kinase	1(5)	Nucleoside diphosphate kinase	1(5)	nucleoside diphosphate kinase activity	1(5)	nucleoside diphosphate kinase activity	1(5)	Nucleoside diphosphate kinase	1(5)	Nucleoside diphosphate kinase, NDK	1(5)
phosphorelay signal transduction	1(2)	Two-component system response regulator	0(2)	Response regulator receiver domain	0(2)	phosphorelay response regulator activity	1(3)	cyclic-guanylate-specific phosphodiesterase activity	1(3)	Cyclic di-GMP phosphodiesterase PA	1(5)	CheY-like	0(2)
HMG-CoA lyase	1(5)	Hydroxymethylglutaryl-CoA lyase YngG	1(4)	HMGL-like	1(5)	transferase activity, transferring acyl grou	1(3)	2-isopropylmalate synthase activity	0(2)	3-hydroxy-3-isohexenylglutaryl-CoA/	1(5)	Aldolase	0(2)
Ferric-uptake regulator	1(5)	Ferric iron uptake transcriptional regulator	1(5)	Ferric uptake regulator family	1(5)	metal ion binding	1(3)	ferrous iron binding	1(4)	Ferric uptake regulation protein	1(5)	"Winged helix" DNA-binding domain	0(2)
Peptidase M28, SGAP-like	1(5)	Predicted aminopeptidase	1(5)	Peptidase family M28	1(5)	aminopeptidase activity	1(5)	aminopeptidase activity	1(5)	Aminopeptidase	1(5)	Zn-dependent exopeptidases	0(2)
4-hydroxybenzoate 3-monooxyg	1(4)	p-hydroxybenzoate hydroxylase	1(5)	FAD binding domain	0(2)	flavin adenine dinucleotide binding	0(2)	FAD binding	0(2)	p-hydroxybenzoate hydroxylase	1(5)	FAD/NAD(P)-binding domain	0(2)
Pyrophosphatase, GppA/Ppx-type	1(5)	Exopolyphosphatase / guanosine-5'-triphosphate	1(5)	Ppx/GppA phosphatase family	1(5)	exopolyphosphatase activity	1(5)	exopolyphosphatase activity	1(5)	Exopolyphosphatase	1(5)	Actin-like ATPase domain	0(2)
Betaine aldehyde dehydrogenase	1(5)	Betaine-aldehyde dehydrogenase	1(5)	Aldehyde dehydrogenase family	1(4)	glutamate-5-semialdehyde dehydrogenase	1(4)	betaine-aldehyde dehydrogenase activity	1(4)	NAD/NADP-dependent betaine aldeh	1(5)	ALDH-like	1(4)
Histone deacetylase family	1(5)	Class II histone deacetylase	1(5)	Histone deacetylase domain	1(5)	histone deacetylase activity	1(5)	NAD-dependent histone deacetylase activity (H3-K14 sp	1(5)	Histone deacetylase-like amidohydrol	1(5)	Arginase/deacetylase	1(4)
sulfuric ester hydrolase activity	1(3)	Arylsulfatase	1(5)	Sulfatase	1(3)	arylsulfatase activity	1(5)	arylsulfatase activity	1(5)	Arylsulfatase	1(5)	Alkaline phosphatase-like	0(2)
Lipid A 3-O-deacylase-related	1(3)	Lipid A deacylase PagL	1(5)	Lipid A 3-O-deacylase (PagL)	1(5)	lipid A metabolic process	1(4)	lipid A metabolic process	1(4)	Lipid A deacylase PagL	1(5)	OMPA-like	0(2)
Phosphoserine phosphatase/hom	1(3)	Bifunctional phosphoserine phosphatase/homos	1(5)	haloacid dehalogenase-like hydrolase	0(2)	phosphoserine phosphatase activity	1(5)	phosphoserine phosphatase activity	1(5)	Phosphoserine phosphatase ThrH	1(5)	HAD-like	0(2)
NADH:quinone oxidoreductase, F	1(3)	FMN-dependent NADH-azoreductase	1(5)	Flavodoxin-like fold	1(2)	NAD(P)H dehydrogenase (quinone) activi	1(4)	oxidoreductase activity, acting on NAD(P)H, NAD(P) as	1(4)	FMN-dependent NADH-azoreductase	1(4)	Flavoproteins	0(2)
Pilus retraction protein PilT/PilU	1(5)	Type IV pilus twitching motility protein PilT	1(5)	Type II/IV secretion system protein	1(5)	ATPase activity	1(3)	type IV pilus biogenesis	1(4)	Twitching mobility protein	1(3)	P-loop containing nucleoside triphosphate hy	0(2)
No hit found	0(2)	Type IV fimbrial biogenesis protein PilY1	1(5)	Neisseria PilC beta-propeller domain	0(2)	metal ion binding	0(2)	type IV pilus biogenesis	1(5)	Type IV pilus biogenesis factor PilY1	1(5)	Quinoprotein alcohol dehydrogenase-like	0(2)
DNA ligase (ATP) activity	1(3)	ATP-dependent DNA ligase	1(3)	DNA polymerase Ligase (LigD)	1(4)	DNA ligation involved in DNA repair	1(3)	DNA ligase (ATP) activity	1(4)	Multifunctional non-homologous end j	1(5)	DNA ligase/mRNA capping enzyme, catalytic	1(3)
Aminotransferase class-III	1(5)	Beta alanine--pyruvate transaminase	1(5)	Aminotransferase class-III	1(5)	intramolecular transferase activity, transfe	1(3)	transaminase activity	1(4)	Beta-alanine--pyruvate aminotransfer	1(5)	PLP-dependent transferases	1(4)
3-hydroxyisobutyrate dehydroge	1(3)	NAD-dependent L-serine dehydrogenase	1(5)	NAD binding domain of 6-phosphogluconate dehy	1(5)	glycerol-3-phosphate dehydrogenase [NA	1(3)	NADP binding	1(3)	NAD-dependent L-serine dehydrogen	1(5)	NAD(P)-binding Rossmann-fold domains	1(3)
Lipopolysaccharide core heptose	1(5)	Lipopolysaccharide core heptose kinase RfaP	1(5)	Lipopolysaccharide kinase (Kdo/WaaP) family	1(5)	lipopolysaccharide core region metal	1(4)	lipopolysaccharide core region biosynthetic process	1(4)	Lipopolysaccharide core heptose(I) k	1(5)	Protein kinase-like (PK-like)	1(3)
Trans-2-enoyl-CoA reductase	1(3)	Enoyl-[acyl-carrier-protein] reductase [NADH]	1(5)	Trans-2-enoyl-CoA reductase catalytic region	1(3)	enoyl-[acyl-carrier-protein] reductase (NA	1(5)	enoyl-[acyl-carrier-protein] reductase (NADH) activity	1(5)	Enoyl-[acyl-carrier-protein] reductas	1(5)	No hit found	0(2)
CRISPR-associated endoribonucle	1(5)	CRISPR-associated endonuclease Csy4	1(5)	CRISPR-associated protein (Cas_Csy4)	1(5)	maintenance of CRISPR repeat elements	1(4)	maintenance of CRISPR repeat elements	1(4)	CRISPR-associated endonuclease Ca	1(5)	No hit found	0(2)
Type III pantothenate kinase	1(5)	Type III pantothenate kinase	1(5)	Type III pantothenate kinase	1(5)	pantothenate kinase activity	1(4)	pantothenate kinase activity	1(4)	Type III pantothenate kinase	1(5)	Actin-like ATPase domain	1(3)
No hit found	0(2)	Endoproteinase Arg-C	1(3)	No hit found	0(2)	serine-type endopeptidase activity	1(4)	serine-type endopeptidase activity	1(4)	No result	0(2)	Trypsin-like serine proteases	0(2)
Histone deacetylase family	1(2)	Acetylpolyamine amidohydrolase	1(5)	Histone deacetylase domain	1(2)	deacetylase activity	0(2)	NAD-dependent histone deacetylase activity (H3-K14 sp	0(2)	Acetylpolyamine amidohydrolase 2	1(5)	Arginase/deacetylase	0(2)
Transcriptional regulatory protei	0(2)	Alginate biosynthesis regulatory protein AlgR	1(5)	Response regulator receiver domain	1(2)	phosphorelay response regulator activity	0(2)	alginic acid biosynthetic process	1(4)	Positive alginate biosynthesis regulat	1(5)	CheY-like	0(2)
Polyphosphate:AMP phosphotrar	1(5)	Polyphosphate-dependent AMP kinase	1(5)	Polyphosphate kinase 2 (PPK2)	1(5)	polyphosphate kinase activity	1(4)	polyphosphate:AMP phosphotransferase activity	1(5)	Polyphosphate:AMP phosphotransfer	1(5)	P-loop containing nucleoside triphosphate hy	1(3)
Fanconi-associated nuclease 1-lik	1(5)	Fanconi-associated nuclease	1(5)	Fanconi anemia-associated nuclease SAP domain	1(5)	flap-structured DNA binding	0(2)	nuclease activity	1(4)	Fanconi-associated nuclease 1 homol	1(5)	No hit found	0(2)
Lipoxygenase	1(5)	Arachidonate 15-lipoxygenase	1(2)	Lipoxygenase	1(5)	linoleate 13S-lipoxygenase activity	1(5)	linoleate 13S-lipoxygenase activity	1(5)	Linoleate 9/13-lipoxygenase	1(5)	Lipoxigenase	1(5)
Isochorismatase	1(4)	Phenazine biosynthesis protein PhzD	1(5)	Isochorismatase family	1(4)	hydrolase activity, acting on carbon-nitrog	0(2)	phenazine biosynthetic process	1(4)	Phenazine biosynthesis protein PhzD1	1(5)	Isochorismatase-like hydrolases	0(2)

Cytokinin riboside 5'-monophosp	1(5)	Cytokinin riboside 5'-monophosphate phosphorib	1(5)	Possible lysine decarboxylase	1(3)	hydrolase activity, hydrolyzing N-glycosyl	1(2)	hydrolase activity, hydrolyzing N-glycosyl compounds	1(4)	Putative cytokinin riboside 5'-monoph	1(5)	MCP/YpsA-like	0(2)
Delta-aminolevulinic acid dehydra	1(5)	Delta-aminolevulinic acid dehydratase	1(5)	Delta-aminolevulinic acid dehydratase	1(5)	porphobilinogen synthase activity	0(2)	protoporphyrinogen IX biosynthetic process	0(2)	Delta-aminolevulinic acid dehydratas	1(5)	Aldolase	0(2)
electron transfer activity	0(2)	Nitrite reductase (NO-forming) / hydroxylamine r	1(5)	Cytochrome D1 heme domain	0(2)	heme binding	0(2)	nitrite reductase (NO-forming) activity	1(5)	Nitrite reductase	1(5)	C-terminal (heme d1) domain of cytochrome	1(4)
nucleotidyltransferase activity	1(3)	DNA polymerase beta domain-containing protein	1(3)	Second Messenger Oligonucleotide or Dinucleotide	1(3)	organic cyclic compound binding	1(3)	metal ion binding	0(2)	DNA polymerase subunit beta	0(2)	Nucleotidyltransferase	0(2)
Type VI secretion system, RhsGE-	1(3)	Actin cross-linking toxin VgrG1	0(2)	Phage late control gene D protein (GPD)	1(2)	metal ion binding	0(2)	ATP binding	0(2)	Uncharacterized protein	0(2)	Phage tail proteins	0(2)
3-hydroxyacyl-CoA dehydrogenas	1(5)	L-carnitine dehydrogenase	1(5)	3-hydroxyacyl-CoA dehydrogenase, NAD binding d	1(2)	glycerol-3-phosphate dehydrogenase [NA	1(3)	carnitine 3-dehydrogenase activity	1(5)	L-carnitine dehydrogenase	1(5)	NAD(P)-binding Rossmann-fold domains	0(2)
dTDP-4-dehydrorhamnose 3,5-ep	1(5)	dTDP-4-dehydrorhamnose 3,5-epimerase	1(5)	dTDP-4-dehydrorhamnose 3,5-epimerase	1(5)	dTDP-4-dehydrorhamnose 3,5-epimerase	1(5)	dTDP-4-dehydrorhamnose 3,5-epimerase activity	1(5)	dTDP-4-dehydrorhamnose 3,5-epimer	1(5)	RmlC-like cupins	0(2)
Di-c-type haem protein, MauG/cy	1(5)	Cytochrome-c peroxidase	1(4)	Cytochrome c	1(5)	cytochrome-c peroxidase activity	1(5)	cytochrome-c peroxidase activity	1(5)	Cytochrome c551 peroxidase	1(5)	Cytochrome c	1(4)
regulation of transcription, DNA-	1(4)	Transcriptional regulator RhlR	1(5)	Bacterial regulatory proteins, luxR family	1(4)	regulation of RNA metabolic process	1(4)	transcription regulatory region sequence-specific DNA b	1(4)	Regulatory protein RhlR	1(5)	Pheromone-binding domain of LuxR-like quo	1(4)
